# Base-promoted cascade recyclization of allomaltol derivatives containing an amide fragment into substituted 3-(1-hydroxyethylidene)tetronic acids

**DOI:** 10.3762/bjoc.20.217

**Published:** 2024-10-14

**Authors:** Andrey Nikolaevich Komogortsev, Constantine Vyacheslavovich Milyutin, Boris Valerievich Lichitsky

**Affiliations:** 1 N.D. Zelinsky Institute of Organic Chemistry, Russian Academy of Sciences, Leninsky Pr., 47, Moscow, 119991, Russian Federation

**Keywords:** allomaltol, base-promoted recyclization, 1,1′-carbonyldiimidazole, 3-hydroxypyran-4-ones, tetronic acid

## Abstract

For the first time, recyclization of allomaltol derivatives with an amide fragment in the side chain were investigated. It was shown that the studied process leads to substituted tetronic acids bearing a pyrrolidinone moiety. The application of 1,1′-carbonyldiimidazole and DBU is necessary for implementation of the considered reaction. Based on the performed research a general method for the synthesis of a wide range of (3*E*,5*E*)-3-(1-hydroxyethylidene)-5-(5-oxopyrrolidin-2-ylidene)furan-2,4(3*H*,5*H*)-diones was elaborated. The advantages of the presented protocol are easily available starting compounds and simple isolation of the target products without chromatographic purification. The synthetic utility of the prepared tetronic acids was demonstrated by further transformations at the hydroxyethylidene fragment. The structures of one obtained tetronic acid and one product of derivatization were confirmed by X-ray analysis.

## Introduction

3-Hydroxypyran-4-one derivatives are an important class of heterocyclic compounds widely represented in various naturally occurring sources [1–4]. Products of this type demonstrate a broad range of biological activity [5–13]. For example, 2-hydroxymethyl-5-hydroxypyran-4-one (kojic acid) is a well-known skin whitening agent extensively used in the cosmetic, medicine and food industries. The action of this compound is based on inhibition of tyrosinase activity, which helps to protect against ultraviolet radiation [14–16]. It should be noted that kojic acid is easily produced from carbohydrate sources in multi-tonnage quantity. Another interesting representative of this class is allomaltol readily available from kojic acid. Due to significant accessibility of the aforementioned 3-hydroxypyran-4-ones these products are widely used in organic synthesis [17–23].

**Scheme 1:**
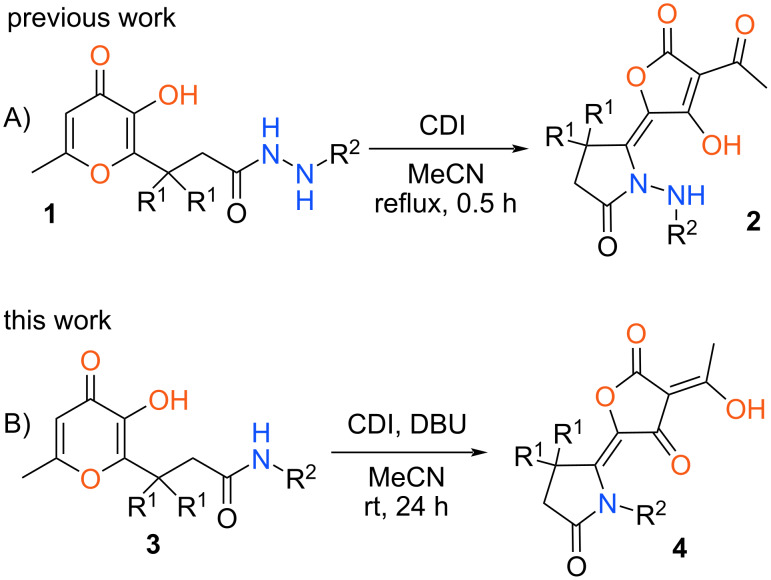
Synthesis of tetronic acid derivatives.

Among the diverse chemical transformations of allomaltol derivatives the recyclizations of the pyranone ring are of great interest. As a rule, such reactions are realized under action of nitrogen-containing nucleophiles and open access to a large array of substituted pyridin-4-ones. Various conditions and reagents employed for these transformations are presented in numerous papers [24–29]. Besides that, approaches to the synthesis of pyridazinones and pyrazoles based on the interaction of 3-hydroxypyran-4-ones with hydrazine are known [30–31]. At the same time all considered recyclizations proceed under action of additional components and the intramolecular variant of this reaction is not described in the literature. It can be supposed that in the case of a substrate containing an allomaltol unit and a nucleophilic fragment in the side chain various transformations are possible. For example, a pyranone ring can be opened and recyclized into the novel heterocyclic system. Indeed, previously we have shown that various allomaltols **1** containing a hydrazide moiety are converted into substituted 3-acetyltetronic acids **2** under action of 1,1-carbonyldiimidazole (CDI) [32] (Scheme 1A). We assumed that the aforementioned synthetic strategy can be extended to other 3-hydroxypyran-4-one derivatives with a nitrogen functional group in the side chain.

Ongoing our research in this area in the present communication we have studied the recyclization of amide-containing allomaltol derivatives **3** under action of CDI (Scheme 1B). In this case, the interaction also leads to substituted tetronic acids **4**. Wherein, in contrast to related hydrazide derivatives **1** the considered process can be realized only in the presence of a strong base (DBU).

## Results and Discussion

**Scheme 2:**
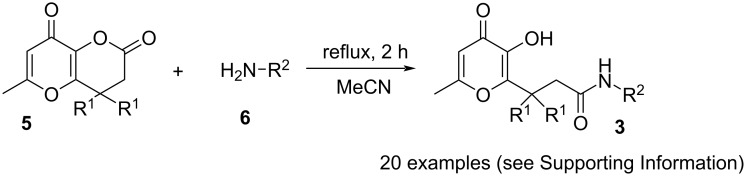
Synthesis of amide derivatives **3**.

The starting allomaltols **3** bearing an amide fragment in the side chain were prepared by reaction of dihydropyranones **5** with appropriate amines **6** applying the previously described method [33–34] (Scheme 2).

At first, amide **3a** was selected as a model object for the present investigation. Initially, we tried to realize the supposed recyclization under conditions suggested earlier for the similar hydrazide derivatives (3 equiv of CDI, MeCN, reflux, 0.5 h). In this case the starting compound **3a** didn’t undergo any transformations and was isolated in unchanged form. It should be mentioned that the key step of the studied recyclization is an intramolecular nucleophilic addition of a nitrogen atom at the double bond of the pyranone ring. Comparing the chemical properties of the considered amides and hydrazides it can be assumed that the nucleophilic activity of the amide nitrogen atom is insufficient for this transformation. In this regard, we hypothesized that application of basic reagent can facilitate this process by generating an anion from the amide moiety. For this purpose, we tested the investigated process using various bases under conditions described earlier for corresponding hydrazides. So, among the employed basic agents (Et_3_N, DABCO, DBU, DMAP, K_2_CO_3_, AcONa) only in the case of DBU the expected recyclization was observed and the target product **4a** was obtained in 15% yield. In order to enhance the yield of compound **4a** we tried to perform this reaction using DBU under various conditions and the results are presented in Table 1.

**Table 1:** Optimization of the reaction conditions^a^.

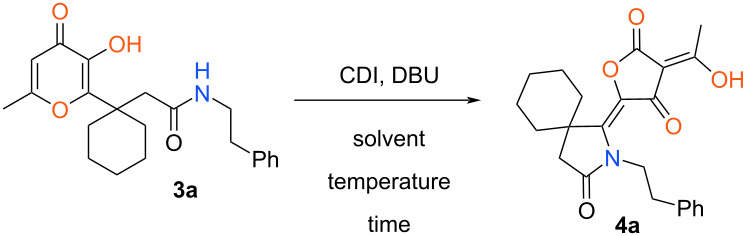				

Entry	Solvent	Temperature	Time, h	Yield, %

1	MeCN	reflux	0.5	15
2	MeCN	reflux	3	22
4	MeCN	reflux	6	18
5	MeCN	rt	3	13
6	MeCN	rt	12	44
7	MeCN	rt	24	62
8	MeCN	rt	48	61
9	dioxane	rt	24	54
10	toluene	rt	24	49
11	CH_2_Cl_2_	rt	24	51

^a^Reaction conditions: **3a** (0.37 g, 1 mmol), CDI (0.49 g, 3 mmol), DBU (0.17 g, 1.1 mmol), solvent (7 mL).

At first, we increased the time of reflux from 0.5 h to 3 h (Table 1, entry 2). As a result, a slight improvement of the yield of product **4a** was achieved. Note that the further elongation of the process time led to decrease of the yield due to partial tarring of the reaction mixture (Table 1, entry 3). Next, we assumed that the investigated reaction can be performed at room temperature. However, stirring under these conditions for 3 h in MeCN didn’t enhance the yield of compound **4a** (Table 1, entry 4). Nevertheless, in this case the increase in process time allowed us to significantly improve the yield of tetronic acid **4a** (Table 1, entry 5). Wherein, the optimal time of the studied recyclization was 24 h (Table 1, entry 6) and further prolongation didn’t influence on the yield (Table 1, entry 7). Then, we tested the investigated reaction in various solvents at room temperature for 24 h. It should be noted that all solvents used had no advantage over MeCN (Table 1, entries 8–11). Thus, the best results for considered recyclization were achieved at stirring of amide **4a** in the presence of CDI and DBU in MeCN at room temperature for 24 h.

**Scheme 3:**
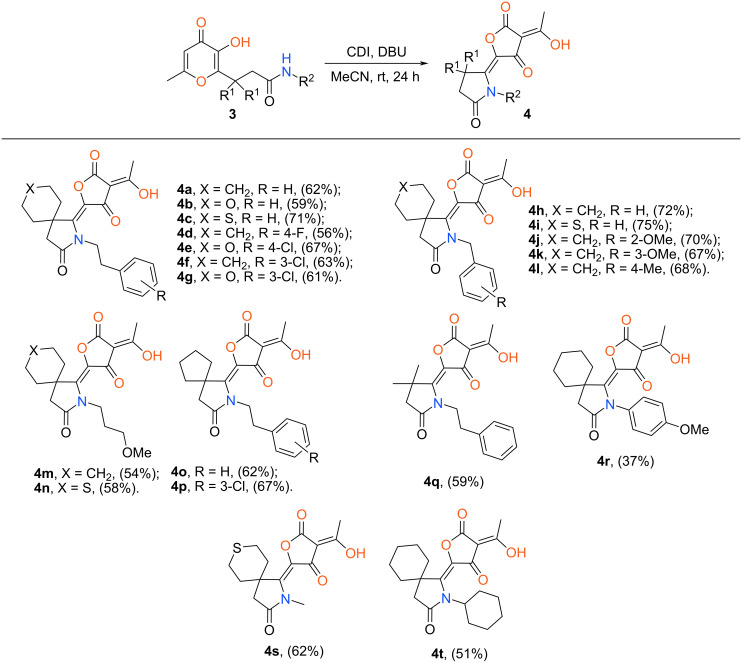
Synthesis of target tetronic acids **4**^a^. ^a^Reaction conditions: **3a** (1 mmol), CDI (0.49 g, 3 mmol), DBU (0.17 g, 1.1 mmol), MeCN (7 mL).

The aforementioned optimal conditions were utilized for the synthesis of a wide range of tetronic acids bearing a pyrrolidinone unit (Scheme 3). The presented protocol is of general nature and allows us to prepare the final products with various aliphatic substituents at the ring nitrogen atom. Besides that, we managed to perform the investigated reaction using the corresponding aniline derivative **4r**. It should be mentioned that in this case the considered recyclization proceeded in lower yield comparing with other amides and was accompanied by formation of various unidentified byproducts.

**Figure 1:**
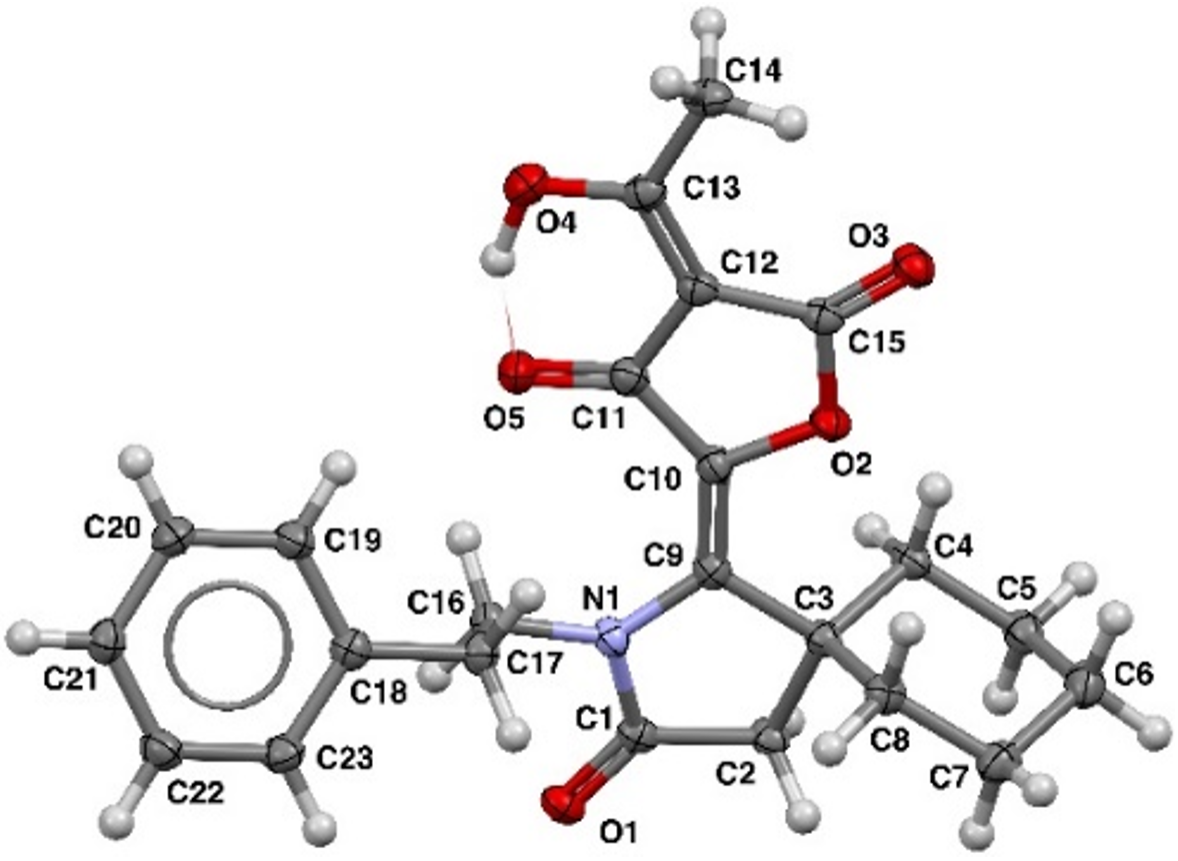
The X-ray crystal structure of compound **4a** (CCDC 2352876).

The obtained tetronic acids **4** are solid crystalline compounds, whose structure was proved by ^1^H, ^13^C NMR spectroscopy and high-resolution mass spectrometry. The ^1^H NMR spectra of the synthesized products contain characteristic signals of protons of the methyl fragment in the region δ = 2.5–2.4 ppm and methylene unit at δ = 2.7–2.5 ppm. The remaining signals are also in good agreement with the presented structure. Besides that, compound **4** has been analyzed using X-ray diffraction (Figure 1). It is important to emphasize that based on X-ray data the structure of synthesized products is different from previously described 3-acetyltetronic acids **2** obtained from the corresponding hydrazides **1**. As well as in the case of compounds **2** for the amide derivatives **4** the double bond between pyrrolidinone and furanone parts has *E*-configuration. Only for sterically hindered compound **4t** with a cyclohexylamine moiety the final product contains 20% of *Z*-isomer. At the same time, the considered products are differed by disposition of double and hydrogen bonds in the tetronic acid moiety. So, in the case of hydrazide derivatives the additional nitrogen atom participates in the formation of an intramolecular hydrogen bond and the double bond is located in the furanone ring. In contrast to the aforementioned example the considered compounds **4** have an exocyclic enol fragment and the intramolecular hydrogen bond is connected to the carbonyl oxygen atom. Thus, the absence of additional nitrogen atoms in compounds **4** leads to the fact that the other tautomeric form becomes more favorable.

**Scheme 4:**
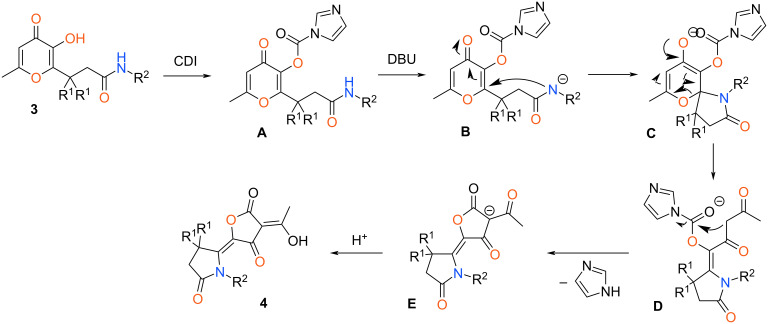
Proposed reaction mechanism for the formation of products **4**.

A proposed mechanism of the investigated recyclization is presented in Scheme 4. Initially, imidazolide **A** is formed via condensation of the starting amide **3** with CDI. Then, intermediate **A** deprotonates to the anion **B** under action of DBU. Subsequent intramolecular cyclization at the double bond of pyranone fragment leads to the formation of spiro compound **C**. Next, opening of the dihydropyranone ring results in pyrrolidin-2-one **D** which further transformed to the salt of 3-acyltetronic acid **E**. Finally, neutralization of intermediate **E** by HCl_conc_ leads to target product **4**.

**Scheme 5:**
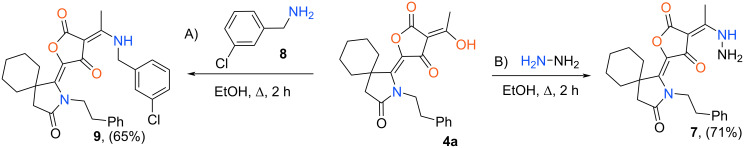
Synthesis of derivatization products **7** and **9**.

**Figure 2:**
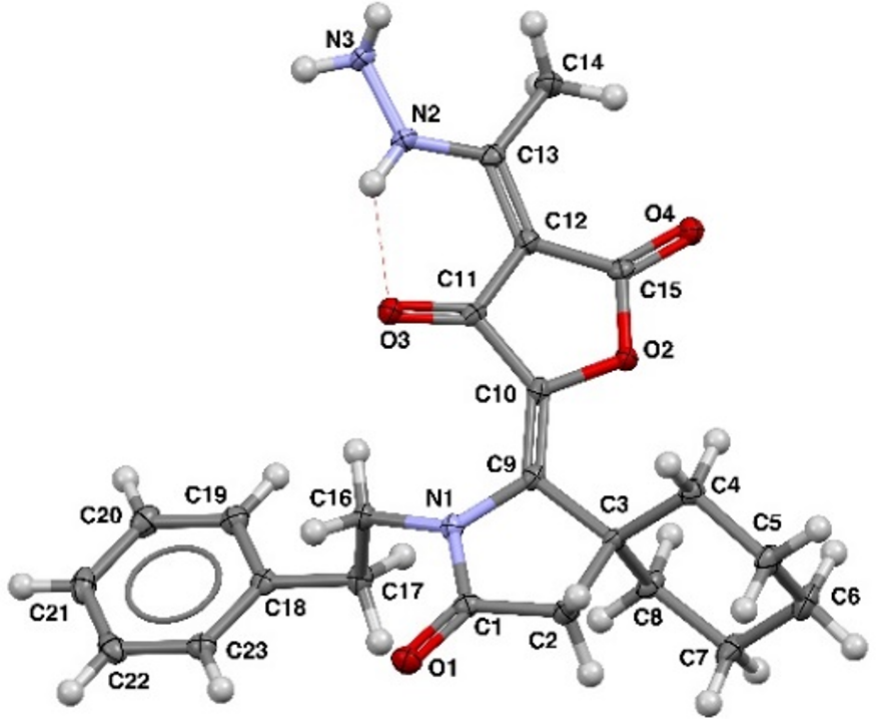
The X-ray crystal structure of compound **7** (CCDC 2352878).

It is interesting to note that chemical properties of the synthesized furanones differ from previously described 3-acetyltetronic acids **2** obtained from the corresponding hydrazides **1**. As it was shown in a prior paper compounds **2** are easily deacetylated under reflux in MeOH for 8 h. At the same time the compounds **4** described in the present communication are more stable and under these conditions the loss of the acetyl group was not observed. It can be supposed that described above difference in configuration of the enol fragments and hydrogen bonds leads to the alteration in chemical behavior. Based on the aforementioned stability of tetronic acids **4** toward action of MeOH we supposed that various derivatives at the hydroxyethylidene group can be synthesized. For example, interaction of compound **4a** with hydrazine in refluxing EtOH allowed us to obtain substituted enehydrazine **7** (Scheme 5a). The similar condensation with amine **8** led to enamine derivative **9** (Scheme 5b). Based on the data of X-ray analysis compound **7** has the same configuration of double and hydrogen bonds as in the case of starting tetronic acids **4** (Figure 2).

## Conclusion

In summary, we have investigated the recyclization of allomaltol derivatives with an amide fragment in the side chain. It was shown that 3-(1-hydroxyethylidene)tetronic acid derivatives containing a pyrrolidinone moiety are formed as a result of the studied reaction. The considered process was performed in the presence of 1,1′-carbonyldiimidazole and DBU using MeCN as a solvent. The set of various (3*E*,5*E*)-3-(1-hydroxyethylidene)-5-(5-oxopyrrolidin-2-ylidene)furan-2,4(3*H*,5*H*)-diones was synthesized employing the developed approach. The readily accessible starting materials and simple purification procedure are the advantages of the considered method. The synthetic utility of obtained tetronic acids was demonstrated by preparation of enamine and enehydrazine derivatives. The structures of one target tetronic acid and synthesized enehydrazine were established by X-ray analysis.

## Experimental

Unless otherwise stated, all starting chemicals were commercially available and were used as received. The starting compounds **1** were prepared by a procedure described in the literature [33–34]. NMR spectra were recorded with Bruker AM 300 (300 MHz) in DMSO-*d*_6_. Chemical shifts (ppm) are given relative to solvent signals (DMSO-*d*_6_: 2.50 ppm (^1^H NMR) and 39.52 ppm (^13^C NMR)). High-resolution mass spectra (HRMS) were obtained on a Bruker micrOTOF II instrument using electrospray ionization (ESI). The melting points were determined on a Kofler hot stage apparatus. A magnetic stirrer IKA C-MAG HS 7 was used for the reactions that require heating.

### General experimental procedure for the synthesis of tetronic acids **4**

A mixture of corresponding amide **3** (1 mmol) and 1,1-carbonyldiimidazole (0.49 g, 3 mmol) was stirred in acetonitrile (7 ml) for 5 min at room temperature, then DBU (0.17 g, 1.1 mmol) was added and resulting solution was kept overnight. After complete the conversion H_2_O (50 mL) and HCl_conc_ (0.7 g) were added to reaction mixture. The precipitated product was filtered off and washed with H_2_O (3 × 5 mL) and Et_2_O (3 × 5 mL).

(3*E*,5*E*)-3-(1-Hydroxyethylidene)-5-(3-oxo-2-phenethyl-2-azaspiro[4.5]decan-1-ylidene)furan-2,4(3*H*,5*H*)-dione (**4a**). Pale yellow powder; yield 62% (0.25 g); mp 121–123 °C; ^1^H NMR (300 MHz, DMSO-*d*_6_) δ 7.31–7.13 (m, 5H), 4.34 (t, *J* = 7.5 Hz, 2H), 2.74 (t, *J* = 7.6 Hz, 2H), 2.56–2.48 (m, 2H in DMSO), 2.46 (s, 3H), 2.14 (t, *J* = 11.8 Hz, 2H), 1.64–1.54 (m, 3H), 1.41–1.09 (m, 5H); ^13^C NMR (75 MHz, DMSO-*d*_6_) δ 178.2, 175.5, 164.8, 138.1, 128.9, 128.3, 126.4, 98.7, 44.3, 44.0, 39.4, 32.6, 32.1, 24.7, 23.9, 21.9; HRMS (ESI-TOF) *m/z*: [M + H]^+^ calcld for C_23_H_26_NO_5_, 396.1805; found, 396.1801.

Analytical data of further synthesized compounds can be found in Supporting Information File 1.

### Experimental procedure for the synthesis of compound **7**

The mixture of tetronic acid **4a** (0.37 g, 1 mmol) and hydrazine hydrate (0.1 g, 2 mmol) in EtOH (5 mL) was refluxed for 2 h. The resulting precipitate was filtered off and washed with EtOH (3 × 5 mL).

**(3*E*,5*E*)-3-(1-Hydrazinylethylidene)-5-(3-oxo-2-phenethyl-2-azaspiro[4.5]decan-1-ylidene)furan-2,4(3*H*,5*H*)-dione (7).** White powder; yield 71% (0.29 g); mp 199–201 °C; ^1^H NMR (300 MHz, DMSO-*d*_6_) δ 12.11 (br. s, 1H), 7.30–7.12 (m, 5H), 5.72 (s, 2H), 4.47 (t, *J* = 7.5 Hz, 2H), 2.68 (t, *J* = 7.7 Hz, 2H), 2.56–2.48 (m, 2H in DMSO), 2.46 (s, 3H), 2.15 (t, *J* = 12.9 Hz, 2H), 1.63–1.53 (m, 2H), 1.40–1.11 (m, 5H); ^13^C NMR (75 MHz, DMSO-*d*_6_) δ 179.8, 175.3, 164.8, 140.4, 138.4, 129.0, 128.4, 128.3, 126.3, 88.98, 43.7, 43.5, 39.7, 32.8, 32.3, 24.9, 22.0, 12.8; HRMS (ESI-TOF) *m/z*: [M + H]^+^ calcld for C_23_H_28_N_3_O_4_, 410.2074; found, 410.2092.

### Experimental procedure for the synthesis of compound **9**

The mixture of tetronic acid **4a** (0.39 g, 1 mmol) and 3-chlorobenzylamine (0.16 g, 1.1 mmol) in EtOH (5 mL) was refluxed for 2 h. The resulting precipitate was filtered off and washed with EtOH (3 × 5 mL).

**(3*E*,5*E*)-3-(1-((3-Chlorobenzyl)amino)ethylidene)-5-(3-oxo-2-phenethyl-2-azaspiro[4.5]decan-1-ylidene)furan-2,4(3*H*,5*H*)-dione (9).** White powder; yield 65% (0.34 g); mp 158–160 °C. ^1^H NMR (300 MHz, DMSO-*d*_6_) δ 11.59 (s, 1H), 7.53–7.41 (m, 3H), 7.41–7.35 (m, 2H), 7.23–7.10 (m, 4H), 4.82 (d, *J* = 6.1 Hz, 2H), 4.42 (t, *J* = 7.6 Hz, 2H), 2.67 (t, *J* = 7.8 Hz, 2H), 2.57 (s, 3H), 2.54 (s, 2H), 2.16 (t, *J* = 12.9 Hz, 2H), 1.69–1.53 (m, 3H), 1.42–1.08 (m, 5H); ^13^C NMR (75 MHz, DMSO-*d*_6_) δ 175.7, 171.6, 139.4, 138.7, 138.7, 136.8, 136.6, 136.0, 134.0, 131.3, 129.4, 128.6, 128.4, 128.1, 127.9, 127.8, 126.7, 46.4, 44.3, 44.0, 33.3, 32.6, 25.2, 22.4, 14.8; HRMS (ESI-TOF) *m/z*: [M + H]^+^ calcld for C_30_H_32_ClN_2_O_4_, 519.2045; found, 519.2051.

## Supporting Information

File 1 General information, copies of NMR spectra, X-ray crystallographic data and refinement details.

File 2 Analytical data of all compounds **4**, **7** and **9**.

## Data Availability

All data that supports the findings of this study is available in the published article and/or the supporting information to this article.
