# Supplementary material for: Base-promoted cascade recyclization of allomaltol derivatives containing an amide fragment into substituted 3-(1-hydroxyethylidene)tetronic acids
Source: Beilstein J Org Chem. 2024 Oct 14;20:2585–91. doi: 10.3762/bjoc.20.217 (PMC11496715; doi:10.3762/bjoc.20.217)
Supplement: File 1 — General information, copies of NMR spectra, X-ray crystallographic data and refinement details. [file Beilstein_J_Org_Chem-20-2585-s001.pdf]

BEILSTEIN JOURNAL OF ORGANIC CHEMISTRY

Supporting Information

for

Base-promoted cascade recyclization of allomaltol derivatives containing an amide fragment into substituted 3-(1-hydroxyethylidene)tetronic acids

Andrey N. Komogortsev, Constantine V. Milyutin and Boris V. Lichitsky

Beilstein J. Org. Chem. **2024,** *20,* 2585–2591. doi:10.3762/bjoc.20.217

General information, copies of NMR spectra, X-ray crystallographic data and refinement details

License and Terms: This is a supporting information file under the terms of the Creative Commons Attribution License (https://creativecommons.org/licenses/by/4.0). Please note that the reuse, redistribution and reproduction in particular requires that the author(s) and source are credited and that individual graphics may be subject to special legal provisions.
The license is subject to the *Beilstein Journal of Organic Chemistry* terms and conditions: (https://www.beilstein-journals.org/bjoc/terms)

Table of contents

1.General information S2

2.NMR ^{1}H and ^{13}C spectra for compounds 4 ....S4

3.NMR ^{1}H and ^{13}C spectra for compounds 7 and 9..S24

4.X-ray crystallographic data and refinement details S26

1. General information

General information. Unless otherwise stated, all starting chemicals were commercially available and were used as received. The starting compounds **3** were prepared to a procedure described in the literature [1,2]. NMR spectra were recorded with Bruker AM 300 (300 MHz) in DMSO-d_6. Chemical shifts (ppm) are given relative to solvent signals (DMSO-d_6: 2.50 ppm (^{1}H NMR) and 39.52 ppm (^{13}C NMR). High-resolution mass spectra (HRMS) were obtained on a Bruker micrOTOF II instrument using electrospray ionization (ESI). The melting points were determined on a Kofler hot stage. Magnetic stirrer IKA C-MAG HS 7 was used for the reactions that require heating.

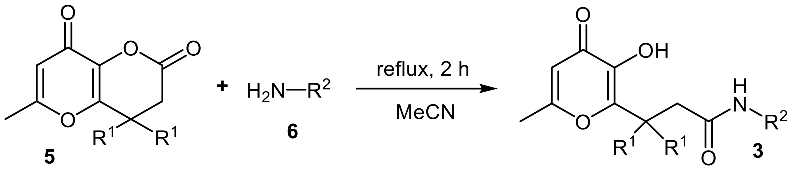

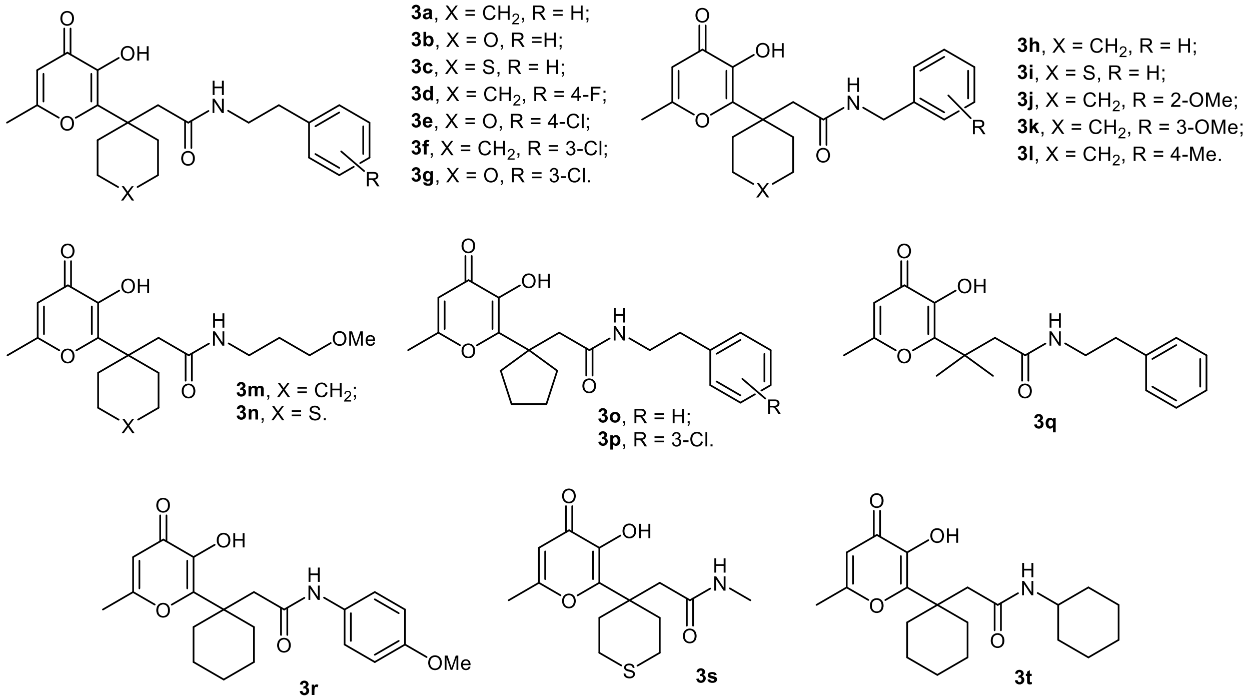

The starting compounds 3 were prepared by previously elaborated method [1,2].

[1] Komogortsev, A. N.; Lichitsky, B. V; Tretyakov, A. D.; Dudinov, A. A.; Krayushkin, M. M. *Chem. Heterocycl. Compd.* **2019**, *55*, 818

[2] Milyutin, C. V; Galimova, R. D.; Komogortsev, A. N.; Lichitskii, B. V; Melekhina, V. G.; Migulin, V. A.; Fakhrutdinov, A. N.; Minyaev, M. E. *Org. Biomol. Chem*. **2021**, *19*, 9975.

General experimental procedure for the synthesis of tetronic acids 4.

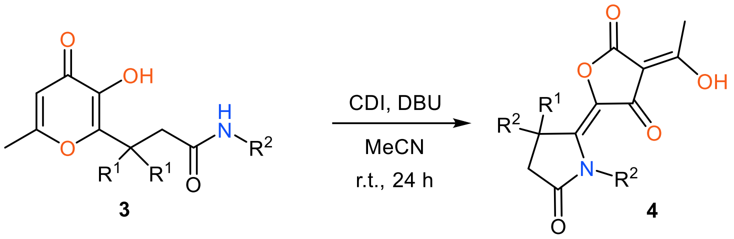

A mixture of corresponding amide **3** (1 mmol) and 1,1-carbonyldiimidazole (0.49 g, 3 mmol) was stirred in acetonitrile (7 ml) for 5 min at room temperature, then DBU (0.17 g, 1.1 mmol) was added and resulting solution was kept overnight. After complete the conversion H_2O (50 ml) and $HCl_{conc.}$ (0.7 g) were added to reaction mixture. The precipitated product was filtered off and washed with H_2O (3 × 5 ml) and Et_2O (3 × 5 ml).

Experimental procedure for the synthesis of compound 7.

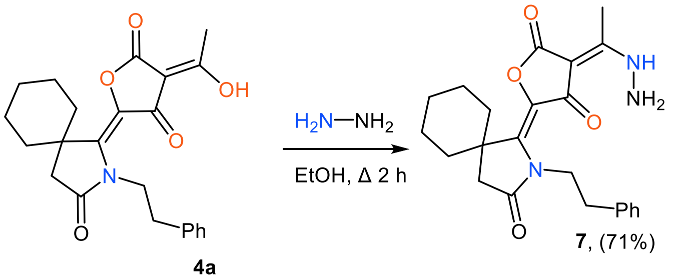

The mixture of tetronic acid **4a** (0.37 g, 1 mmol) and hydrazine hydrate (0.1 g, 2 mmol) in EtOH (5 ml) was refluxed for 2 h. The resulting precipitate was filtered off and washed with EtOH (3 × 5 ml).

Experimental procedure for the synthesis of compound 9.

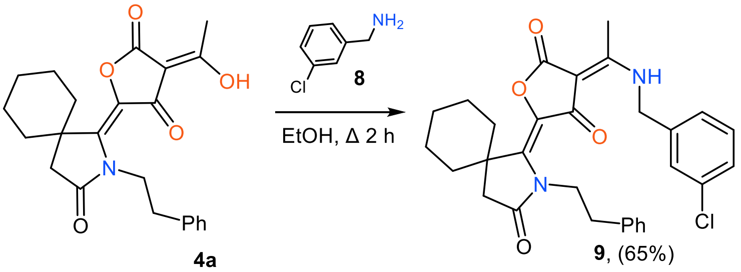

The mixture of tetronic acid **4a** (0.39 g, 1 mmol) and 3-chlorobenzylamine (0.16 g, 1.1 mmol) in EtOH (5 ml) was refluxed for 2 h. The resulting precipitate was filtered off and washed with EtOH (3 × 5 ml).

2. NMR ^{1}H and ^{13}C spectra for compounds 4

^{1}H NMR spectrum (300 MHz) of **4a** in DMSO-d_6

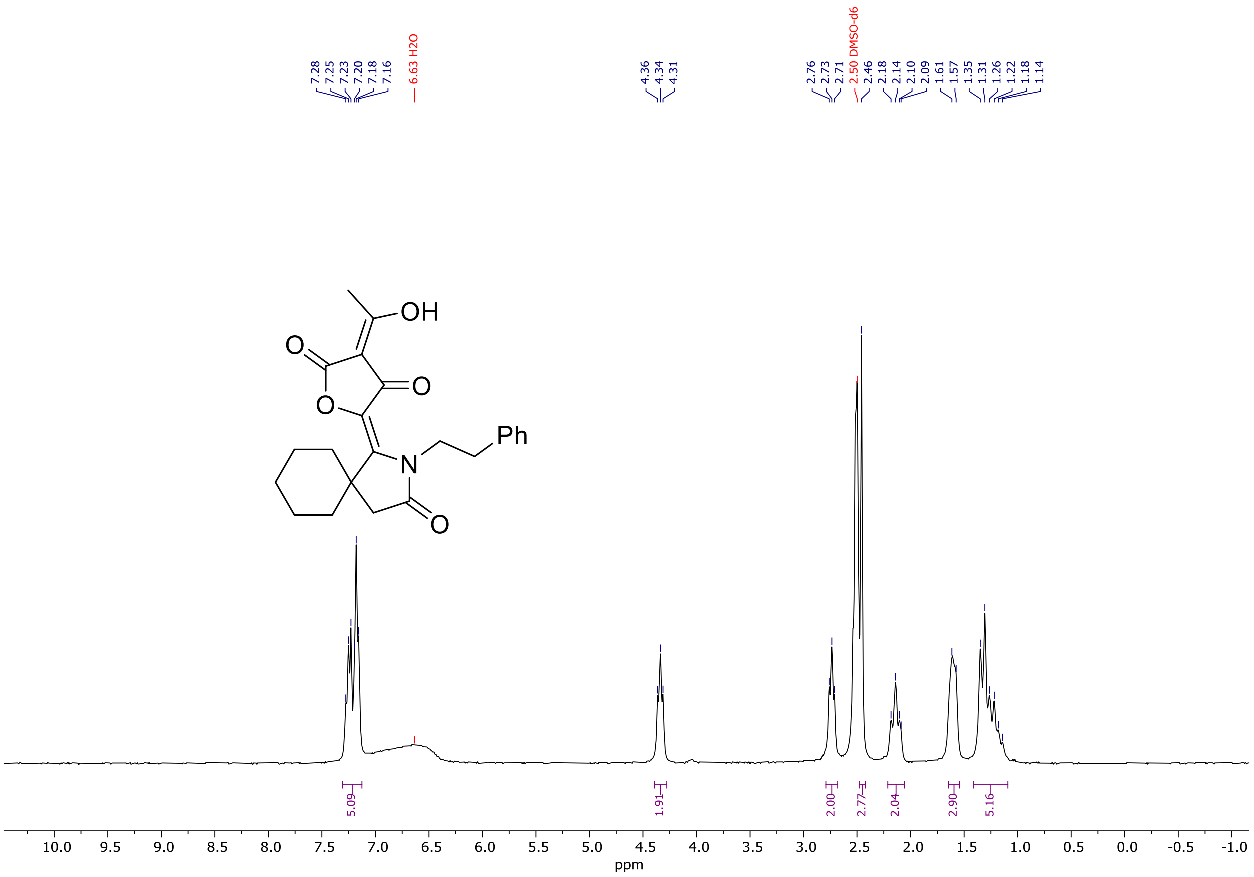

^{13}C {^{1}H} NMR spectrum (75 MHz) of **4a** in DMSO-d_6

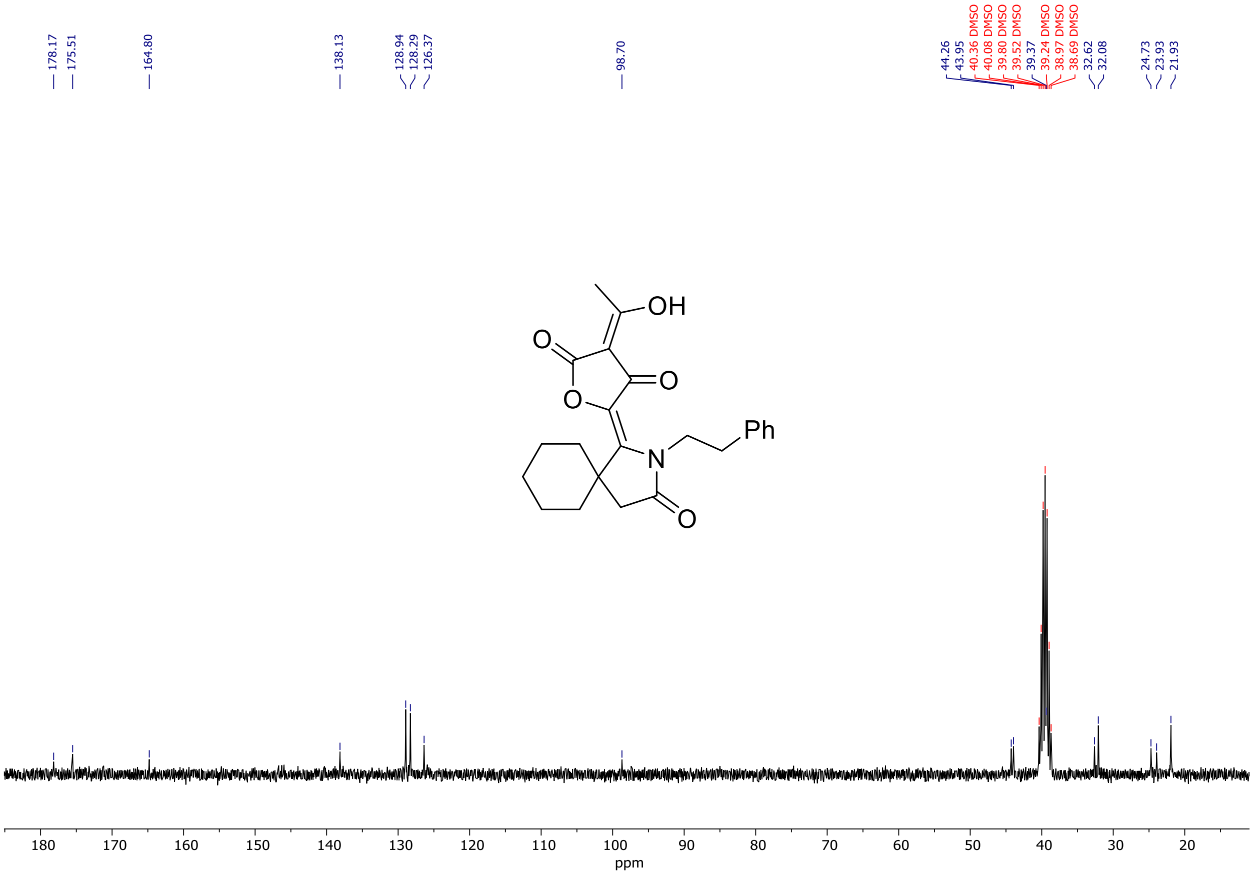

^{1}H NMR spectrum (300 MHz) of **4b** in DMSO-d_6

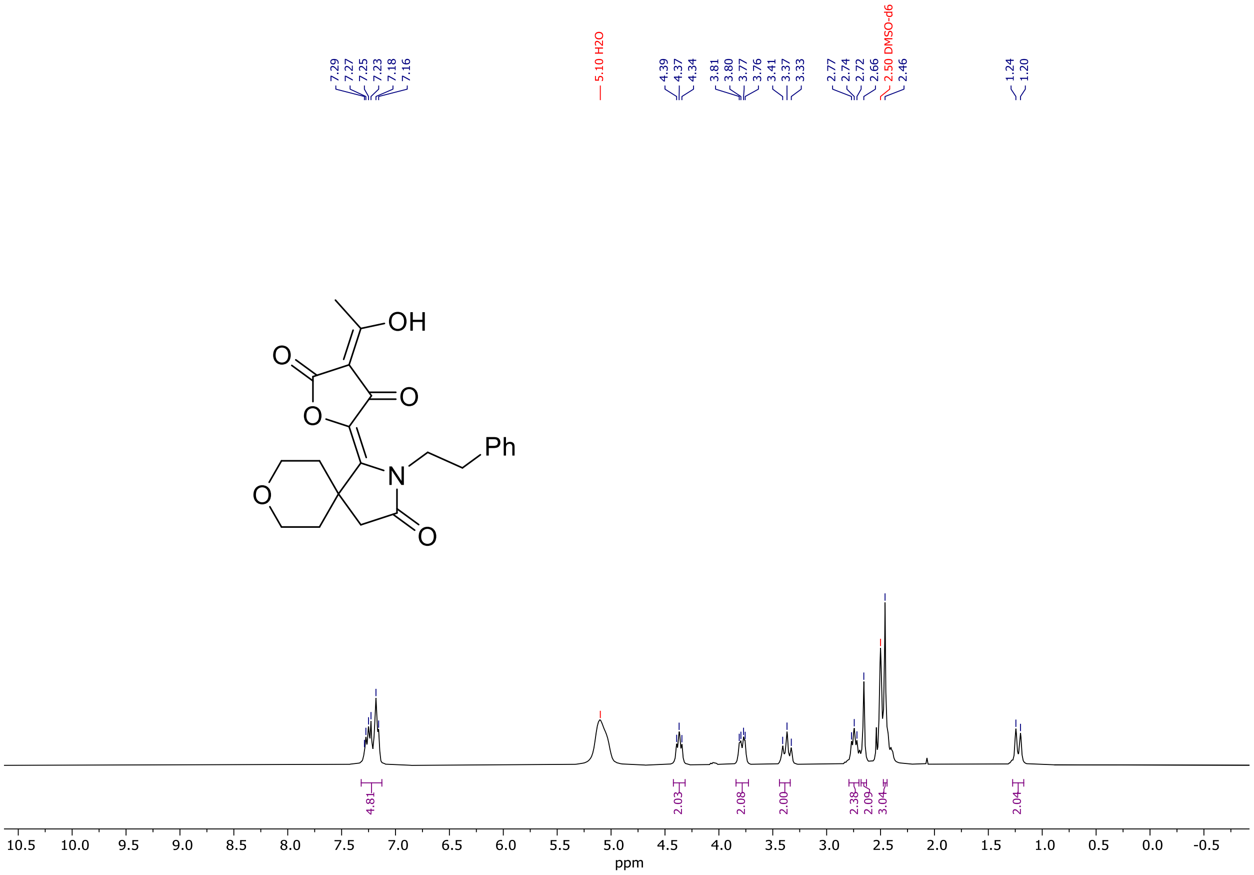

^{13}C {^{1}H} NMR spectrum (75 MHz) of **4b** in DMSO-d_6

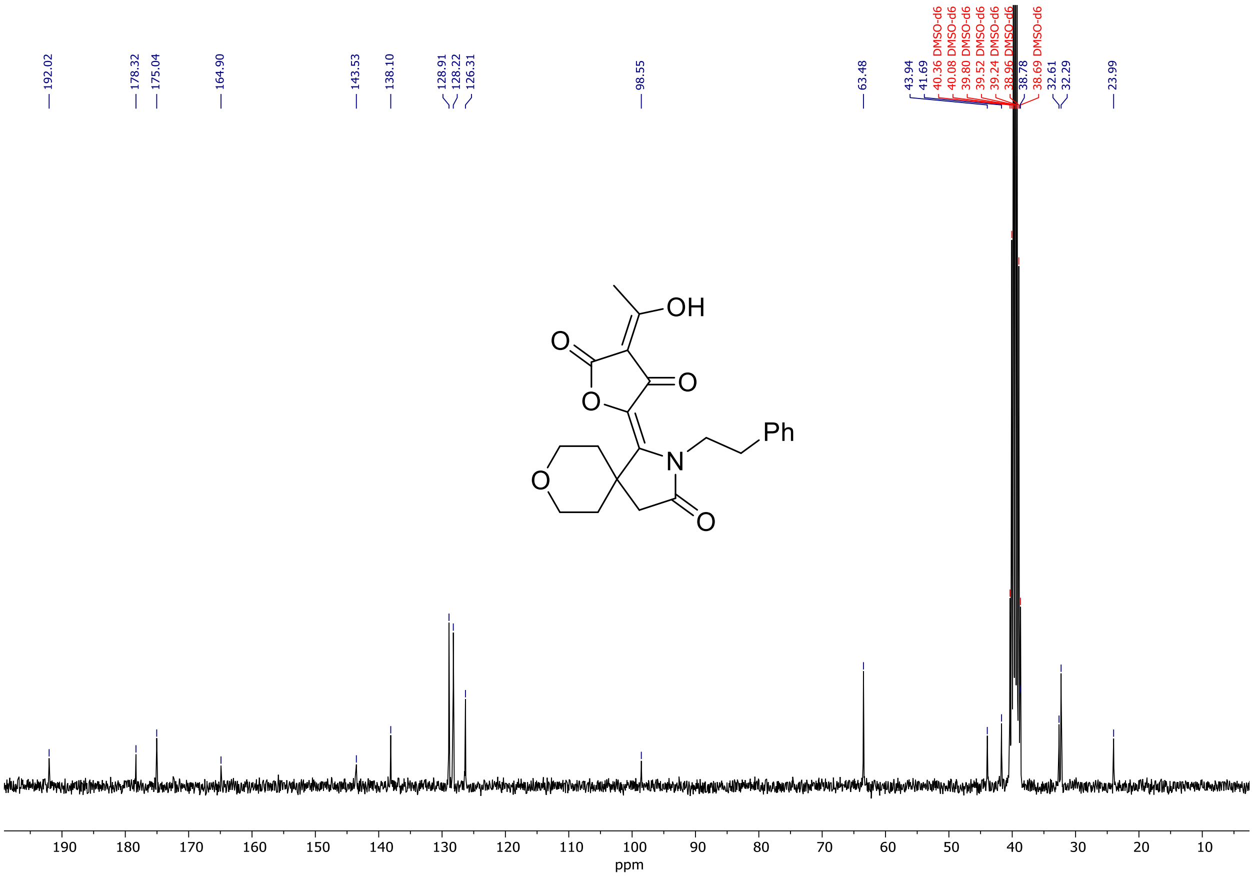

^{1}H NMR spectrum (300 MHz) of **4c** in DMSO-d_6

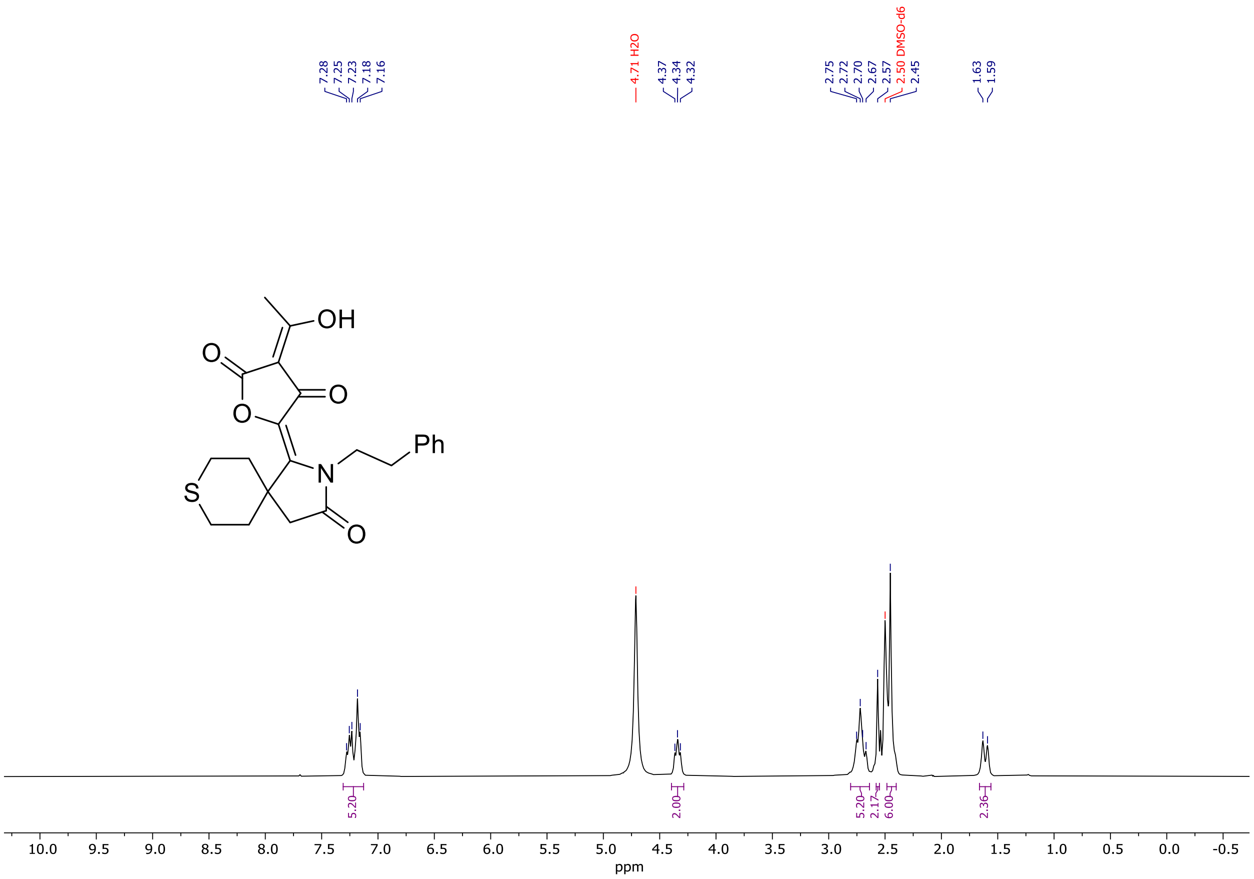

^{13}C {^{1}H} NMR spectrum (75 MHz) of **4c** in DMSO-d_6

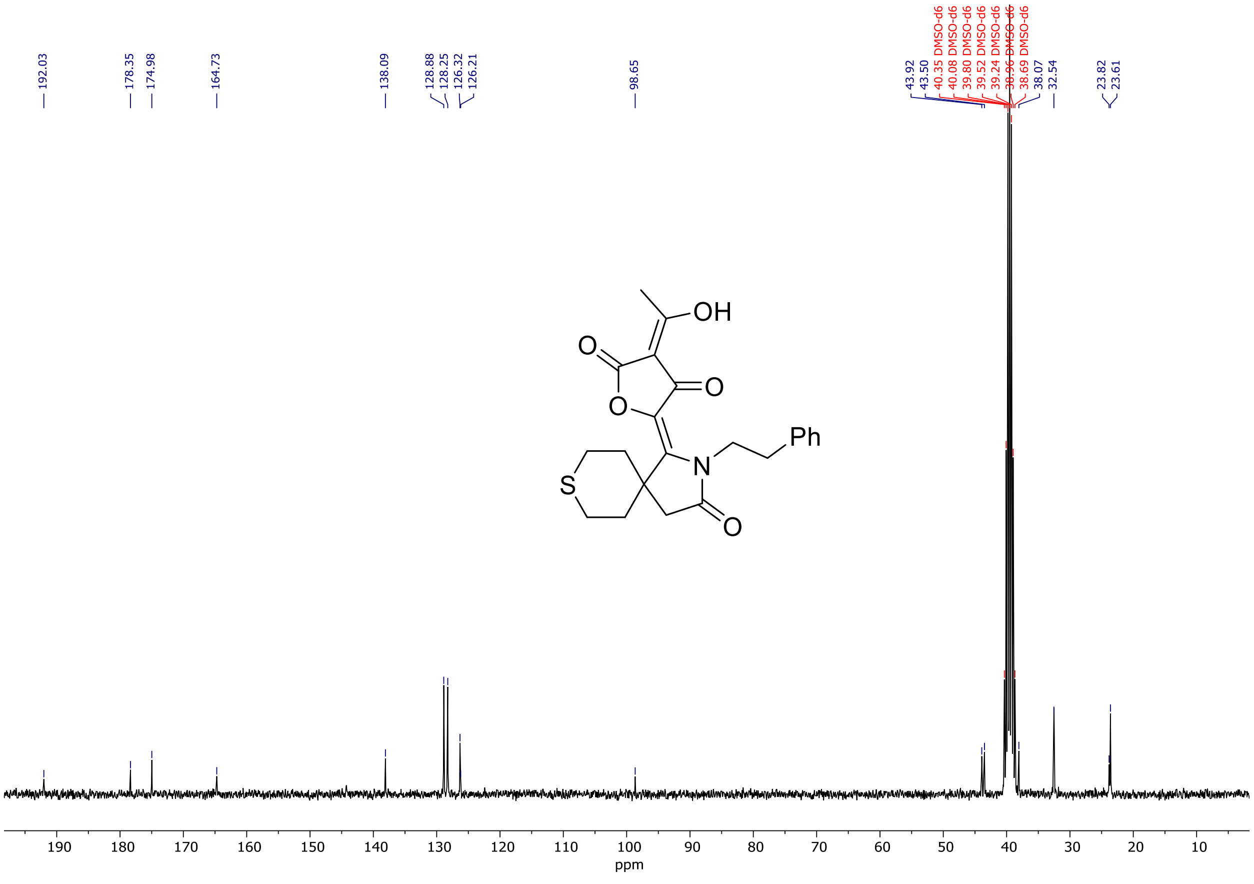

^{1}H NMR spectrum (300 MHz) of **4d** in DMSO-d_6

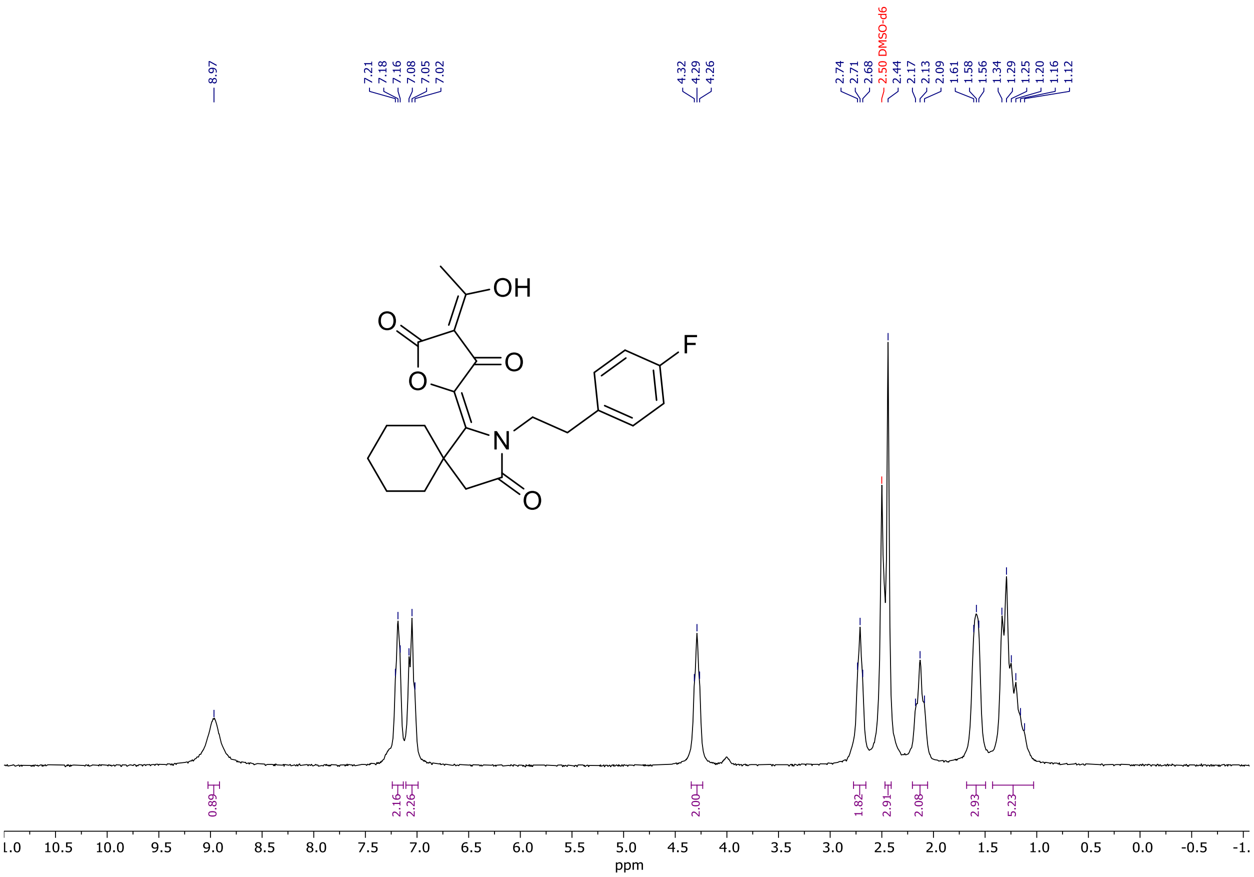

^{13}C {^{1}H} NMR spectrum (75 MHz) of **4d** in DMSO-d_6

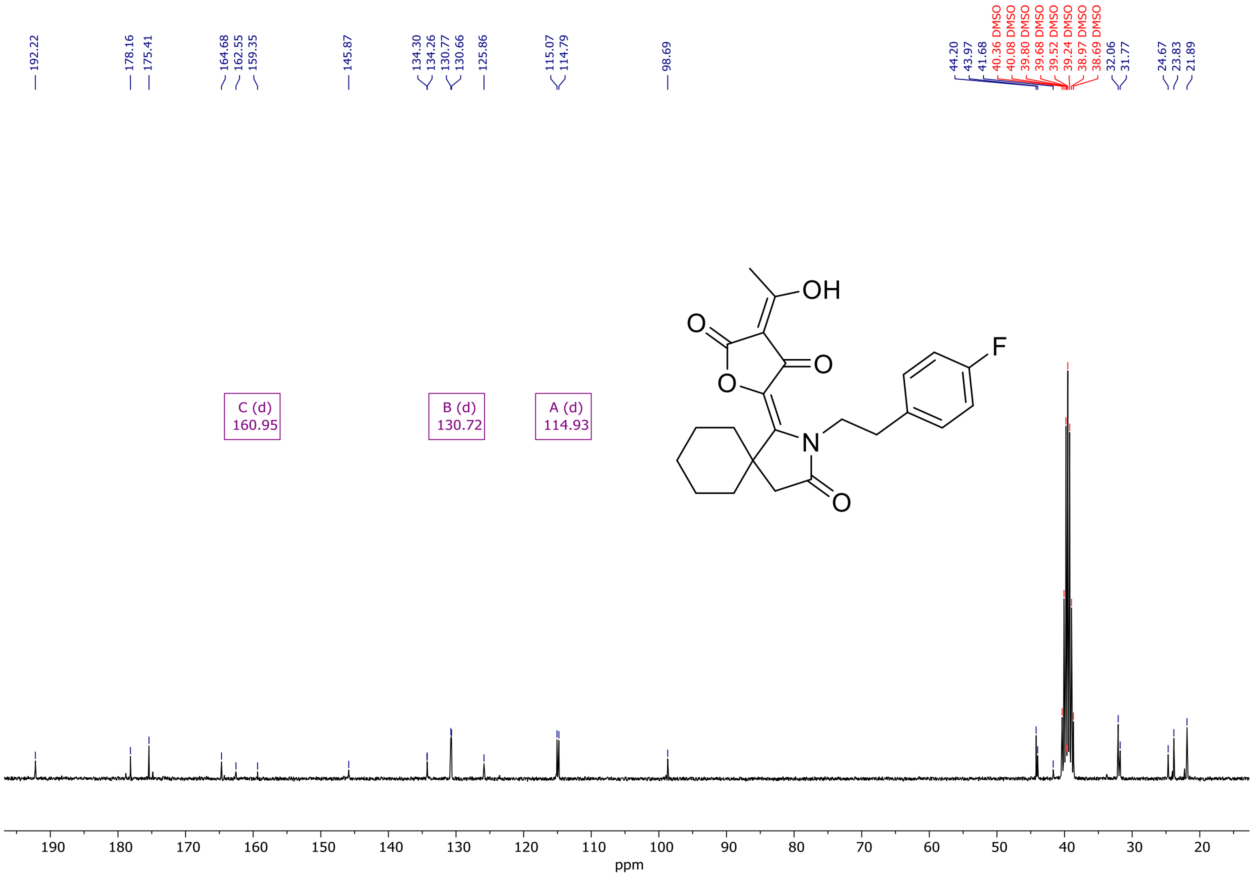

^{1}H NMR spectrum (300 MHz) of **4e** in DMSO-d_6

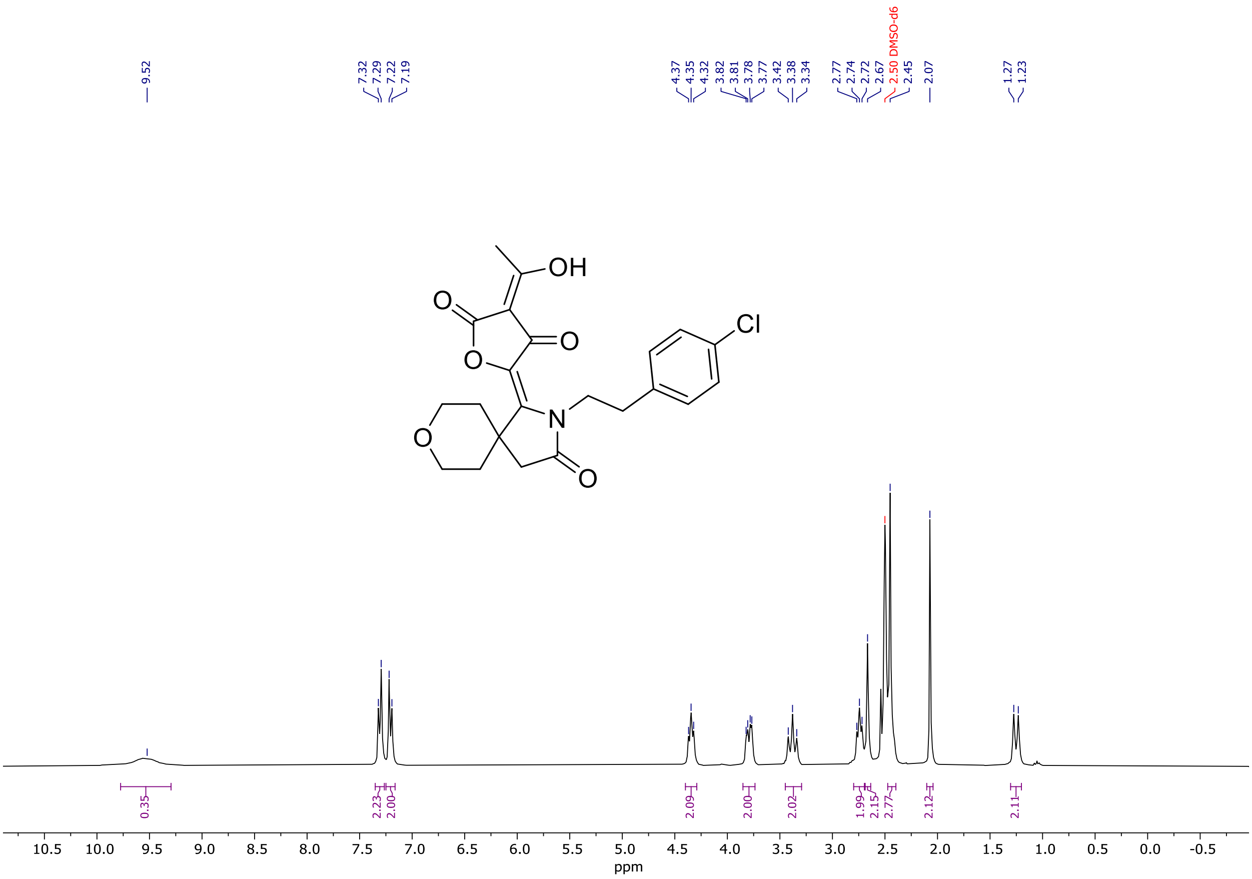

^{13}C $\{^{1}H\}$ NMR spectrum (75 MHz) of **4e** in DMSO-d_6

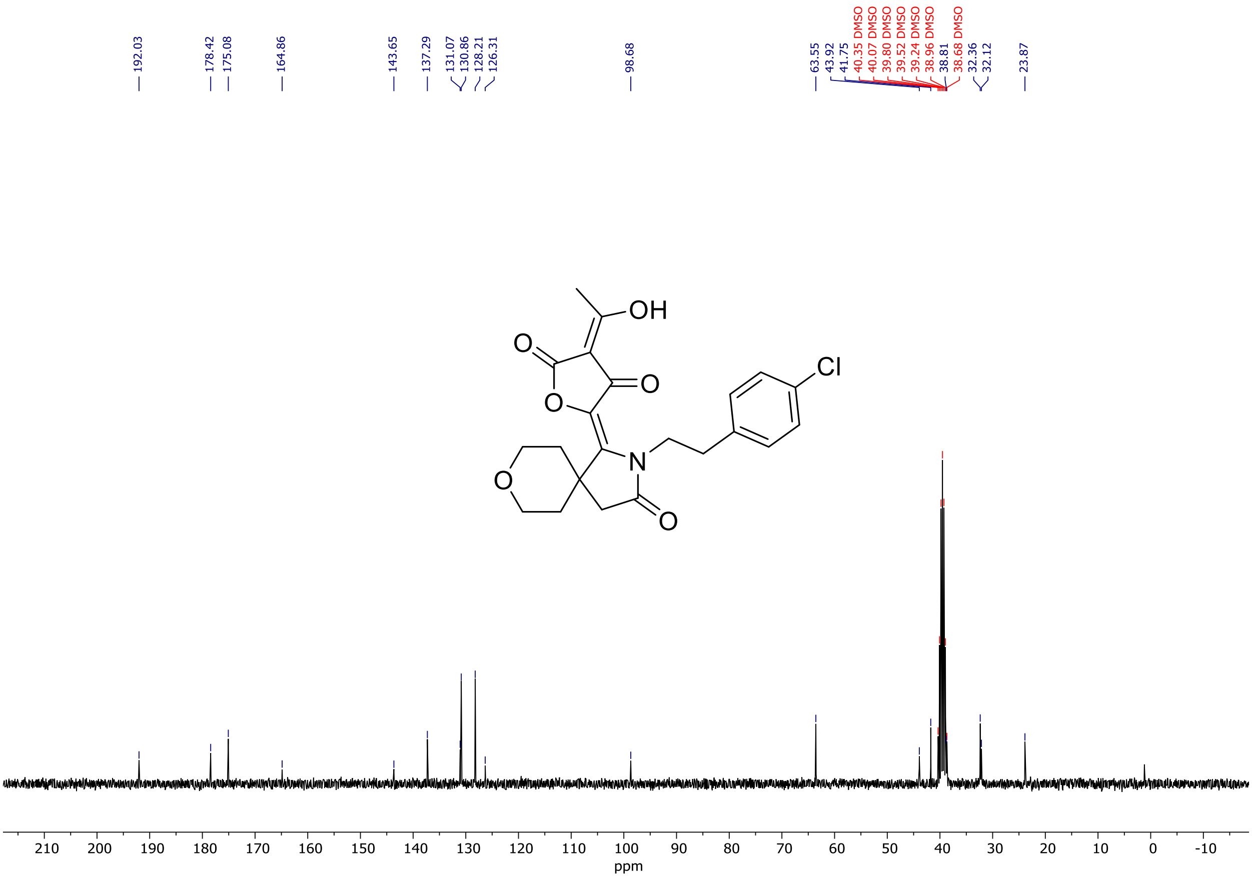

^{1}H NMR spectrum (300 MHz) of **4f** in DMSO-d_6

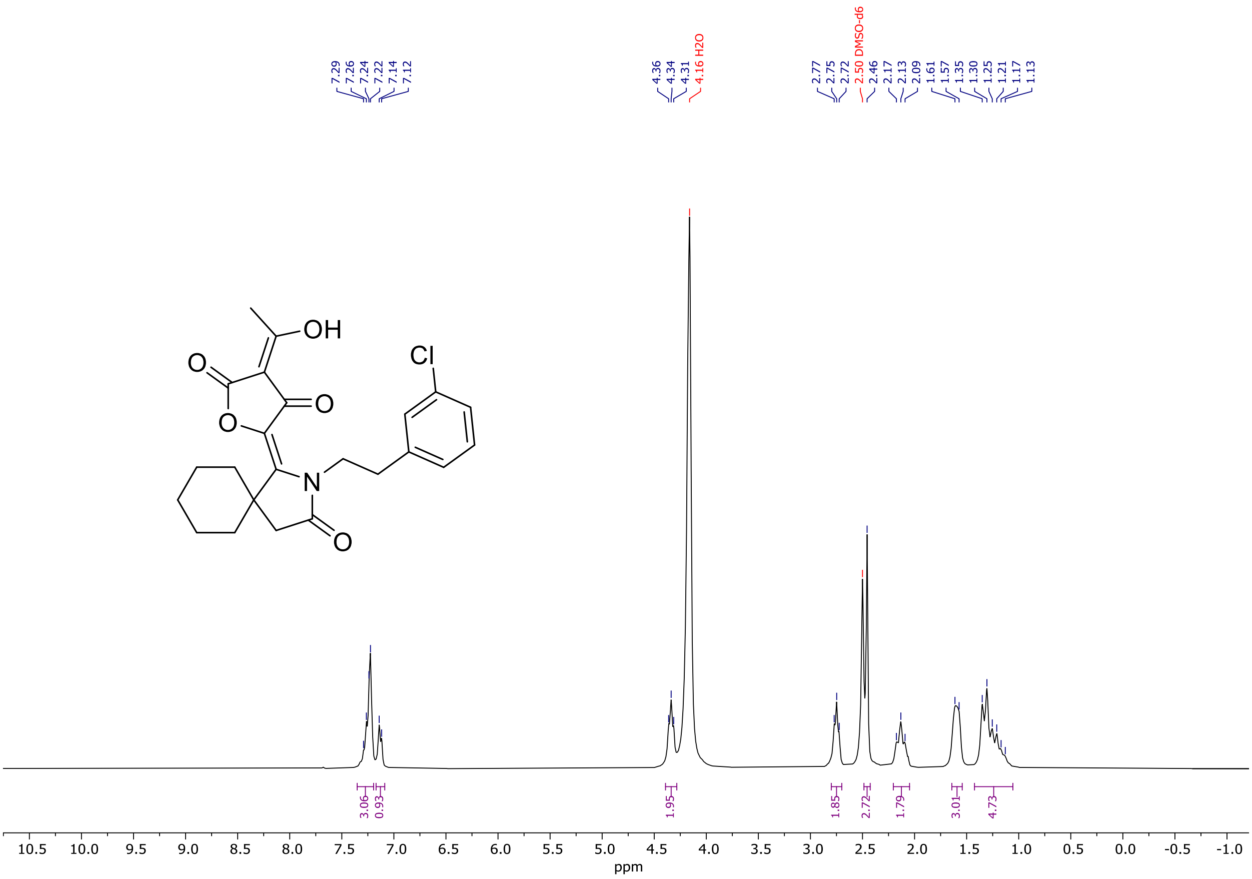

^{13}C {^{1}H} NMR spectrum (75 MHz) of **4f** in DMSO-d_6

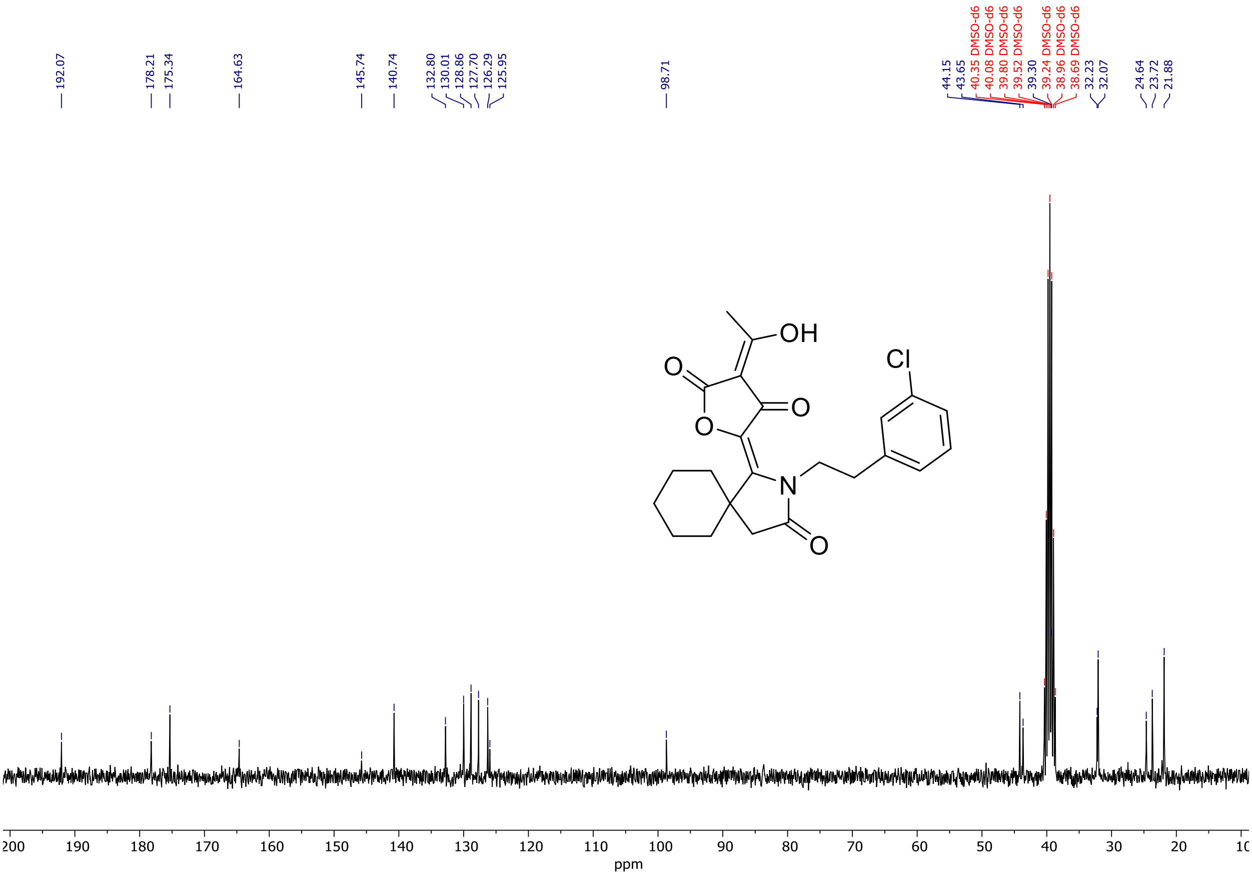

^{1}H NMR spectrum (300 MHz) of **4g** in DMSO-d_6

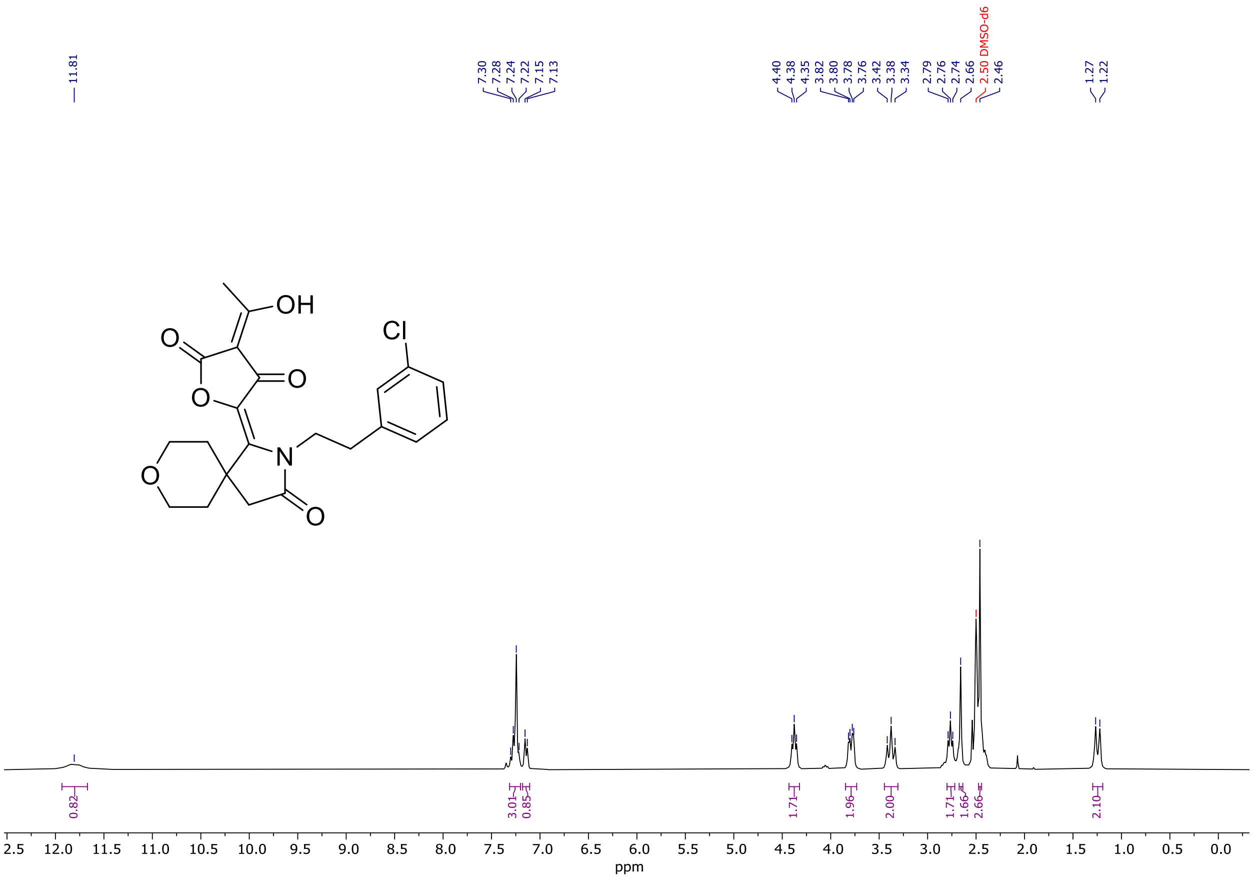

^{13}C {^{1}H} NMR spectrum (75 MHz) of **4g** in DMSO-d_6

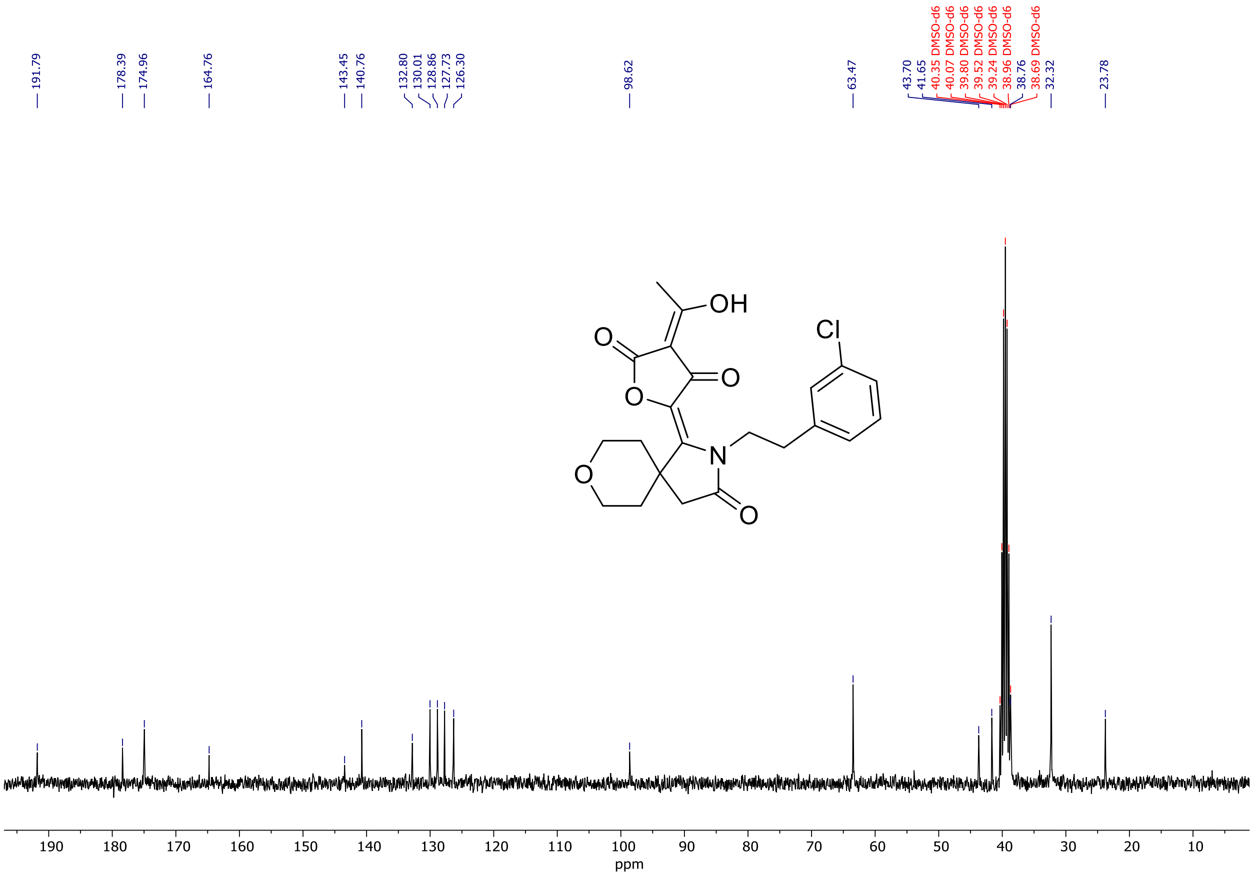

^{1}H NMR spectrum (300 MHz) of **4h** in DMSO-d_6

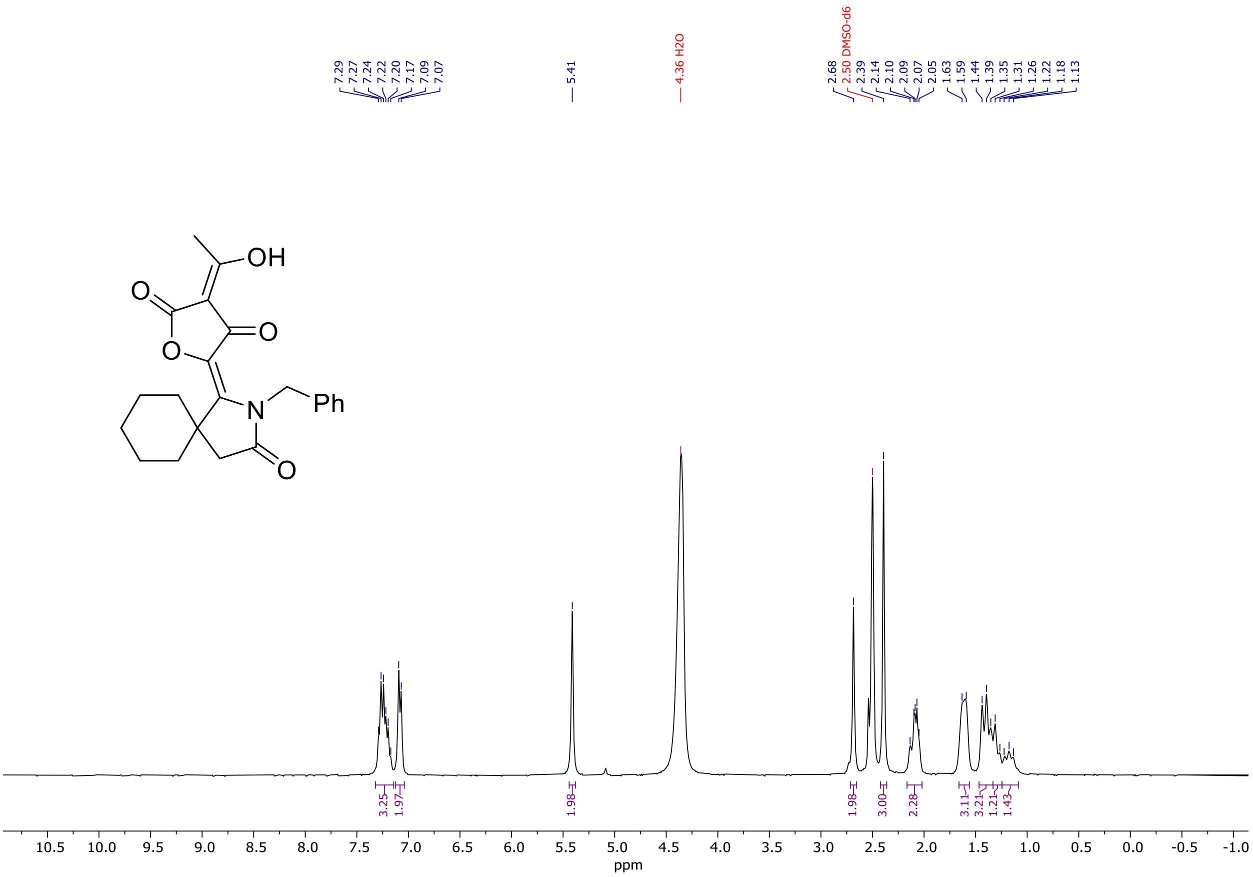

^{13}C {^{1}H} NMR spectrum (75 MHz) of **4h** in DMSO-d_6

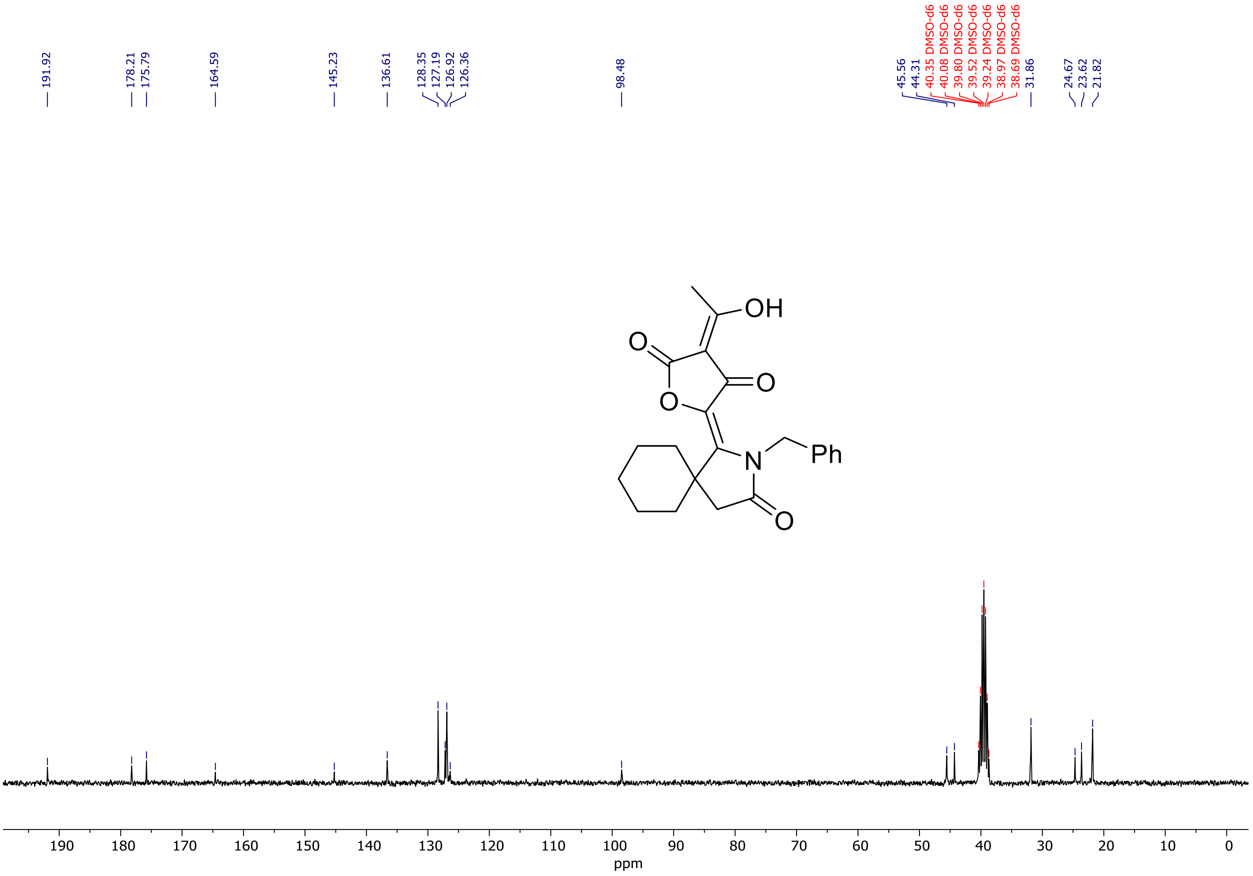

^{1}H NMR spectrum (300 MHz) of **4i** in DMSO-d_6

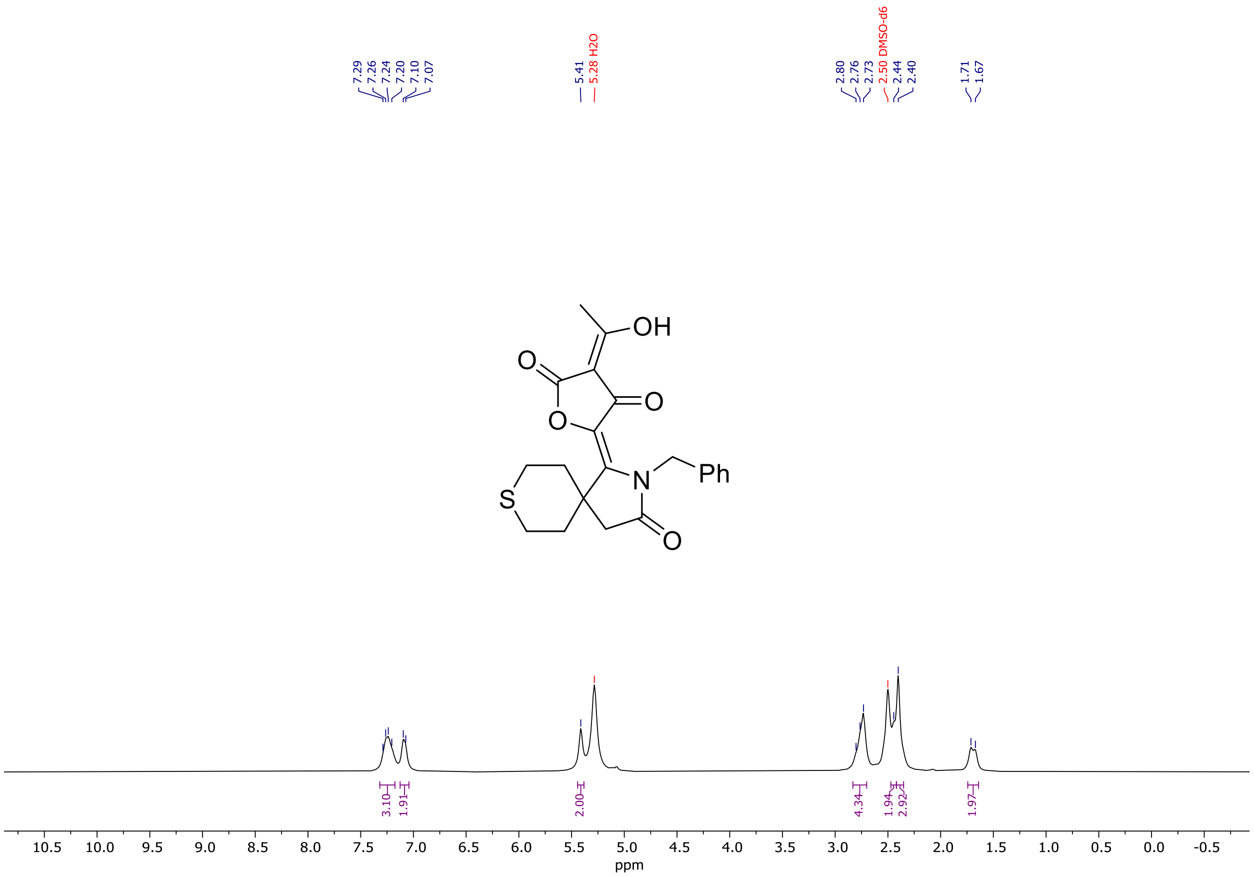

^{13}C {^{1}H} NMR spectrum (75 MHz) of **4i** in DMSO-d_6

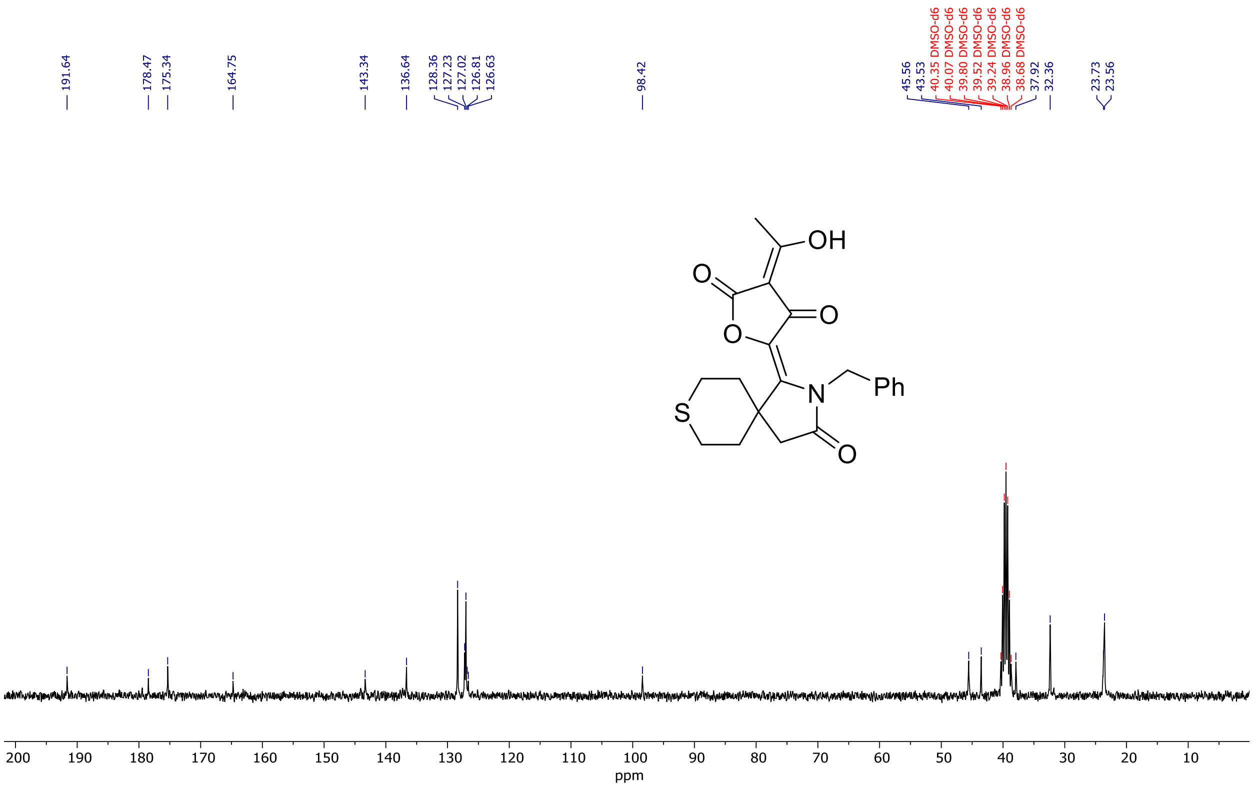

^{1}H NMR spectrum (300 MHz) of **4j** in DMSO-d_6

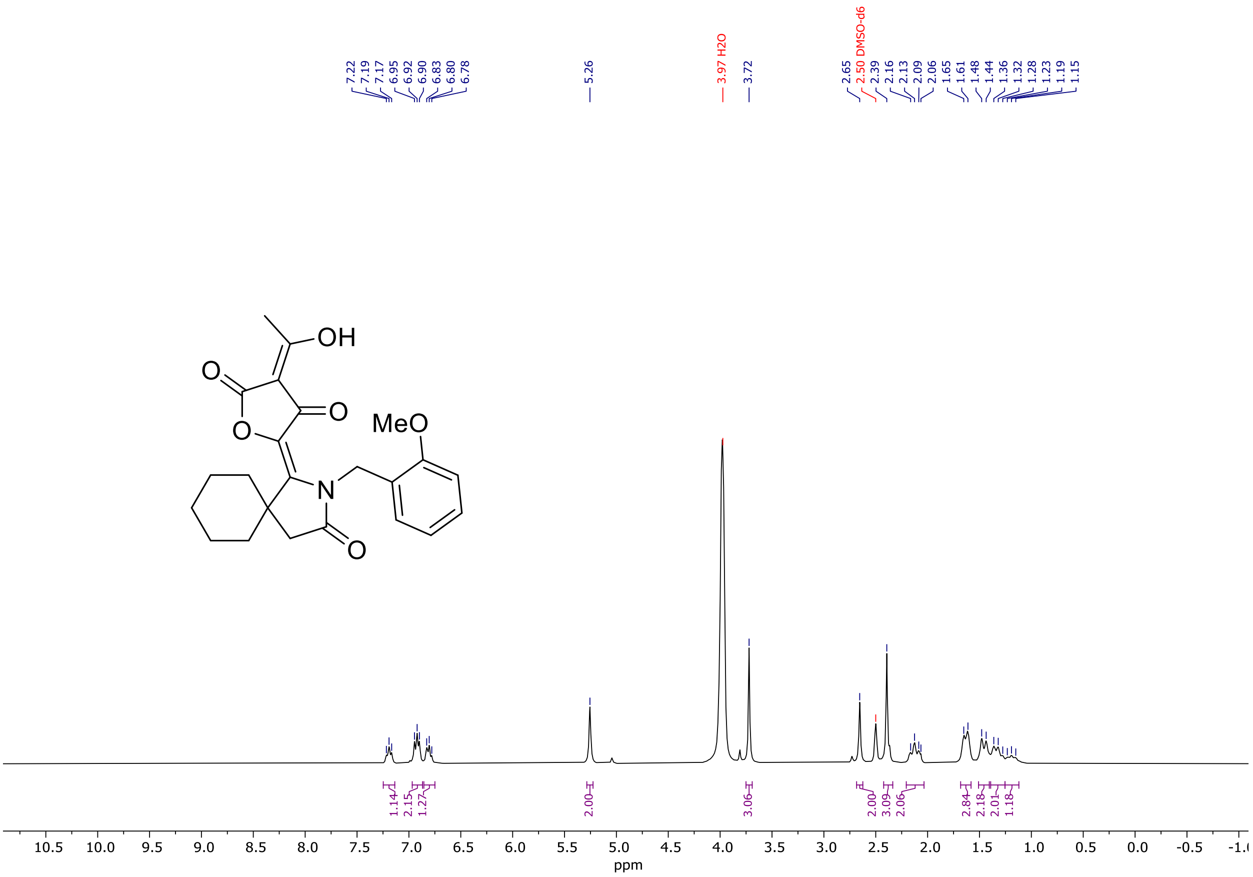

^{13}C {^{1}H} NMR spectrum (75 MHz) of **4j** in DMSO-d_6

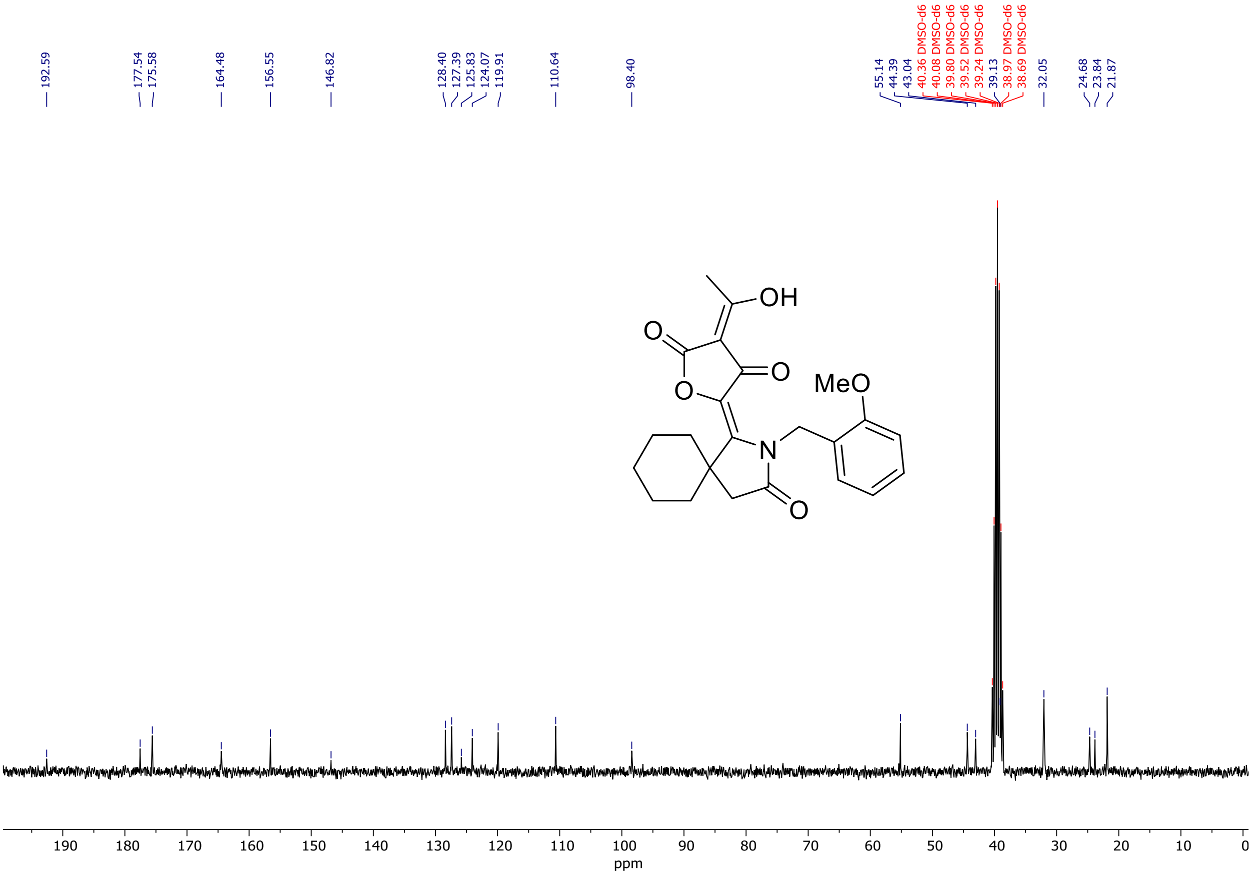

^{1}H NMR spectrum (300 MHz) of **4k** in DMSO-d_6

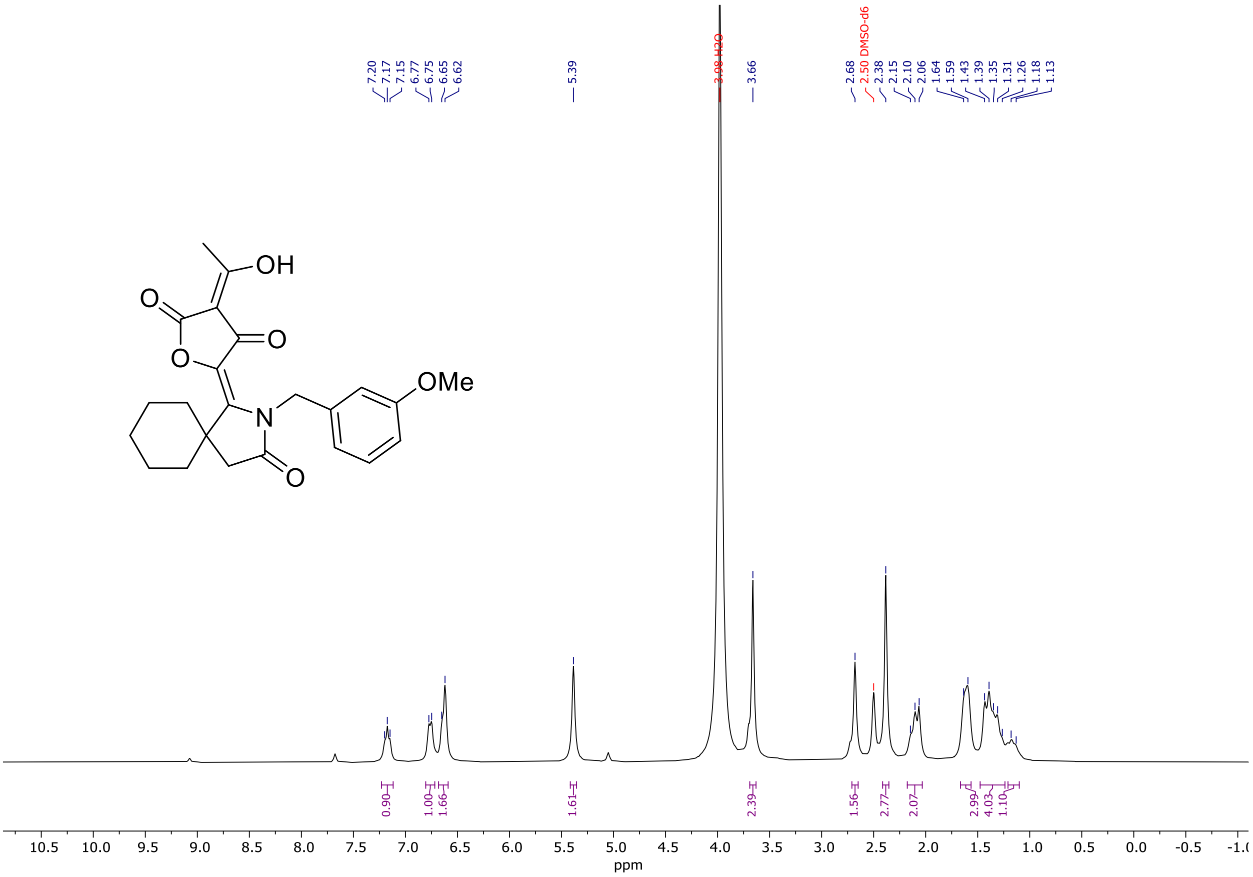

^{13}C {^{1}H} NMR spectrum (75 MHz) of **4k** in DMSO-d_6

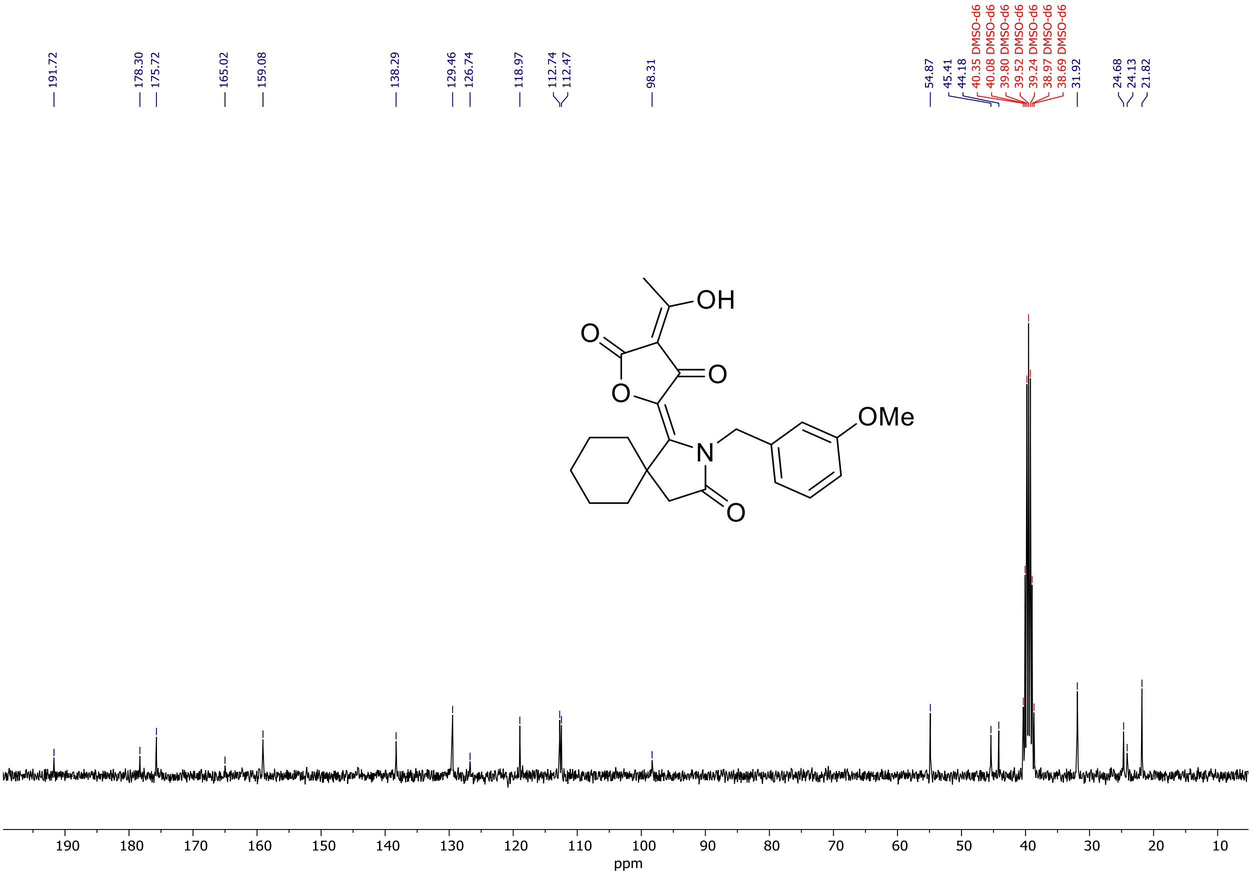

^{1}H NMR spectrum (300 MHz) of **4l** in DMSO-d_6

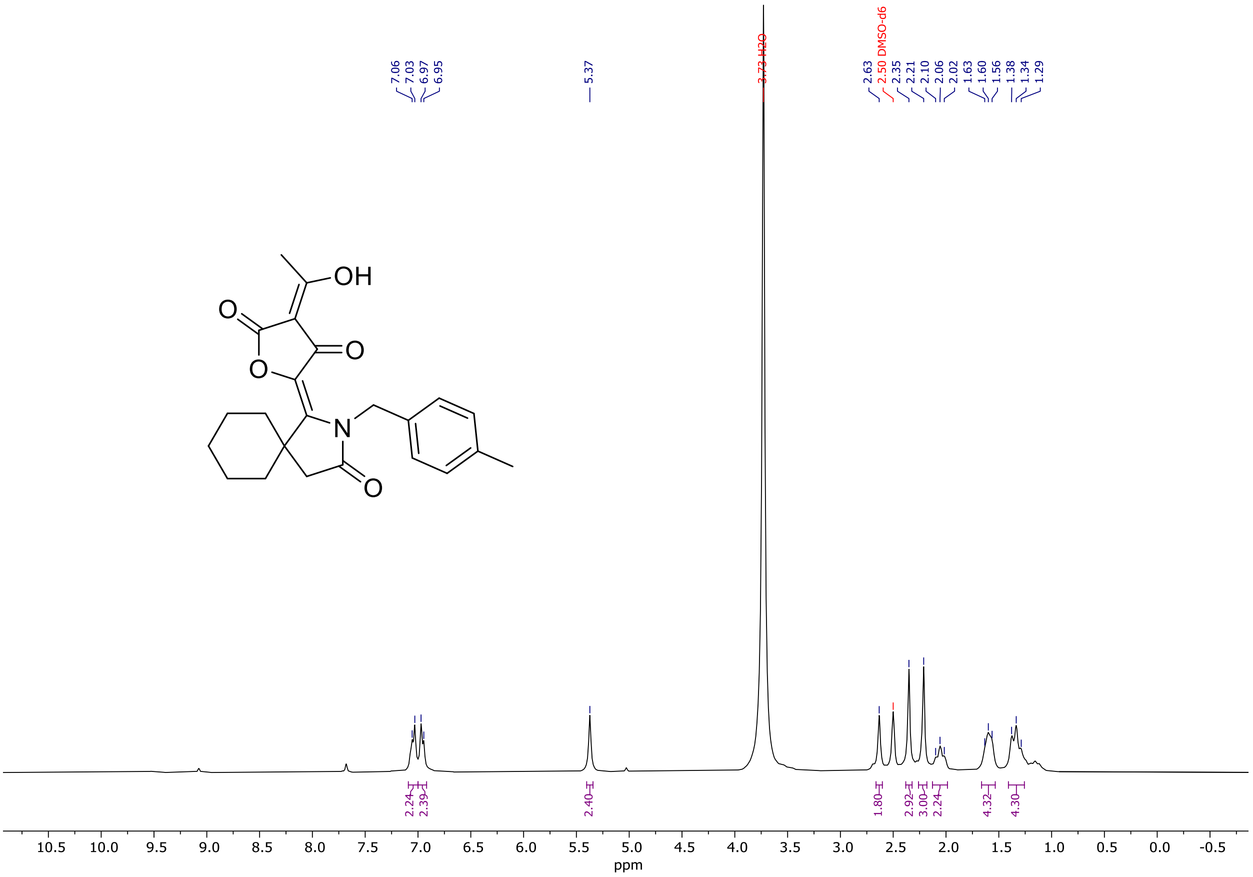

^{13}C {^{1}H} NMR spectrum (75 MHz) of **4l** in DMSO-d_6

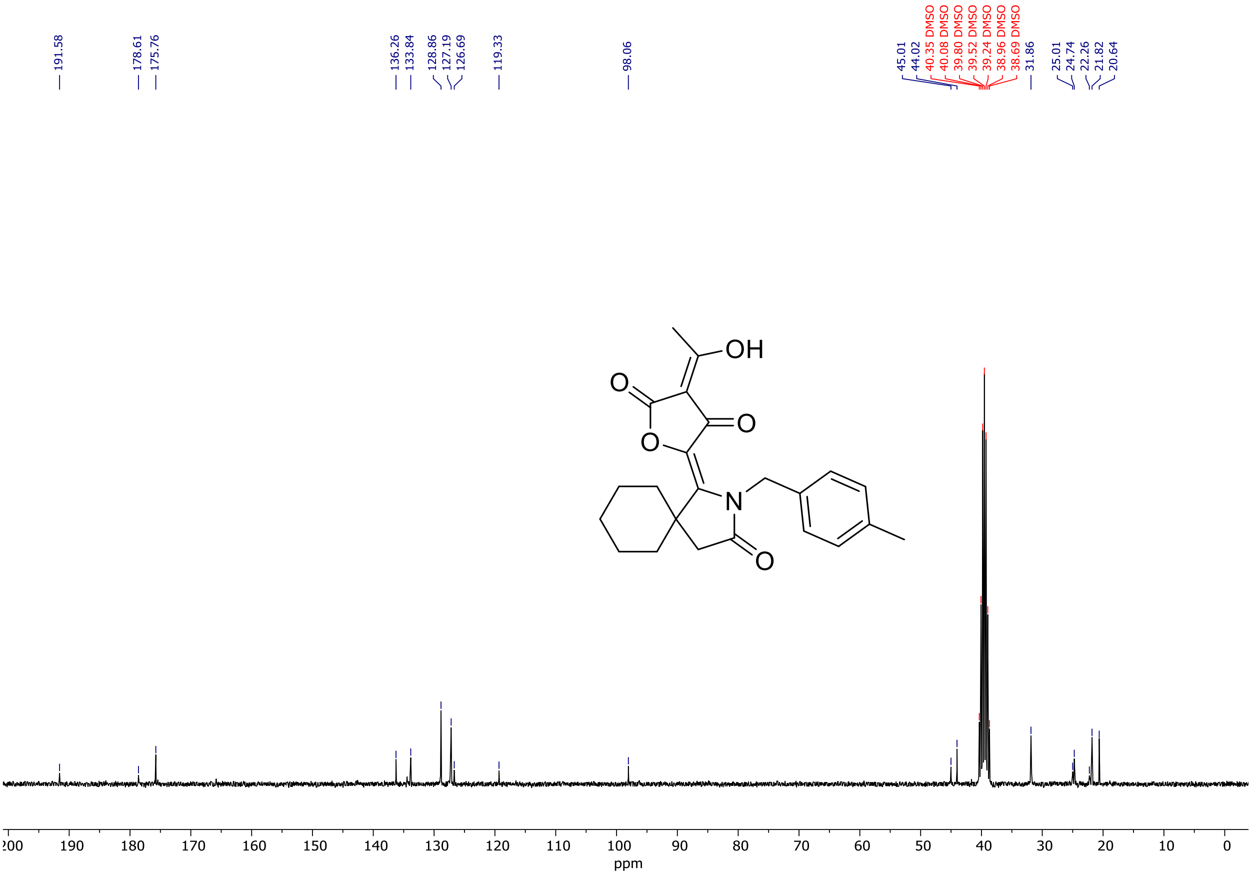

^{1}H NMR spectrum (300 MHz) of **4m** in DMSO-d_6

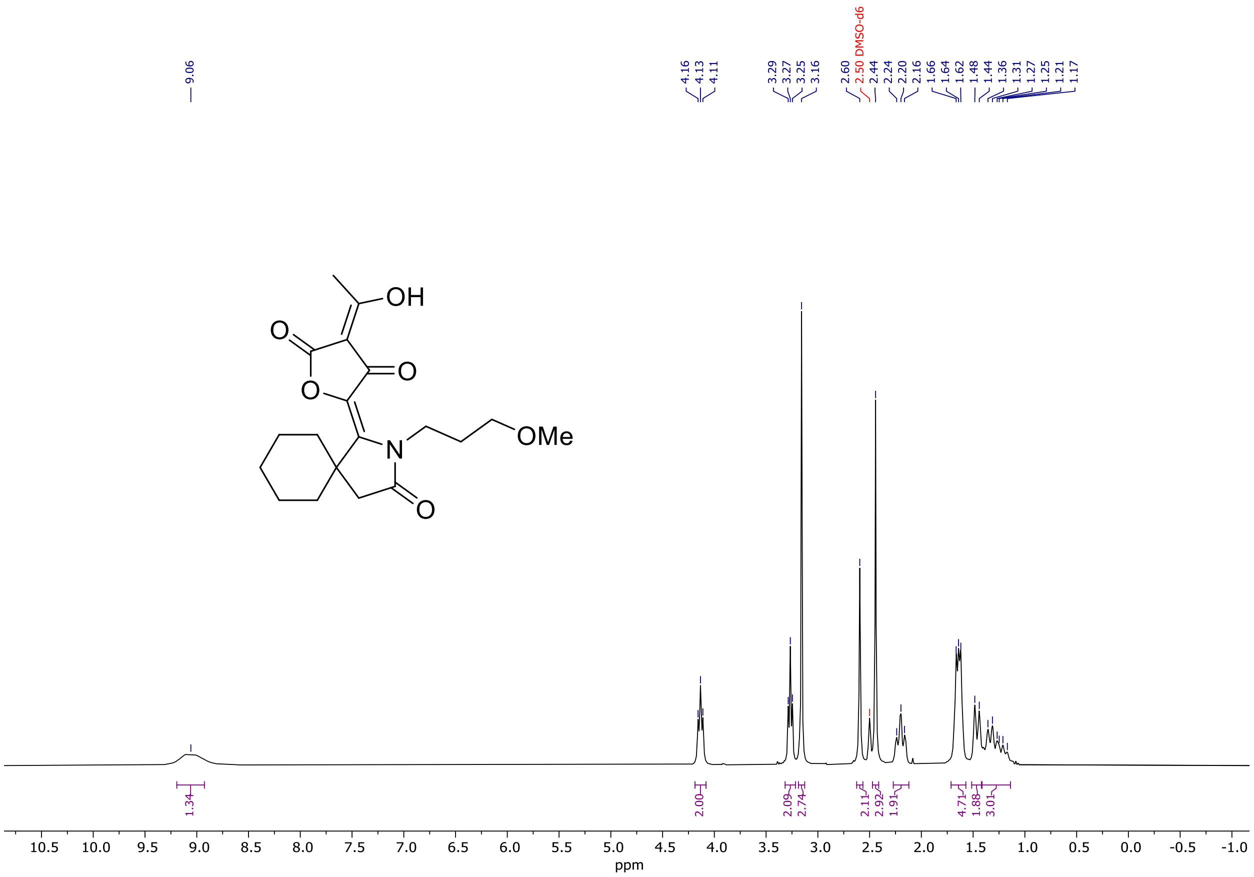

^{13}C {^{1}H} NMR spectrum (75 MHz) of **4m** in DMSO-d_6

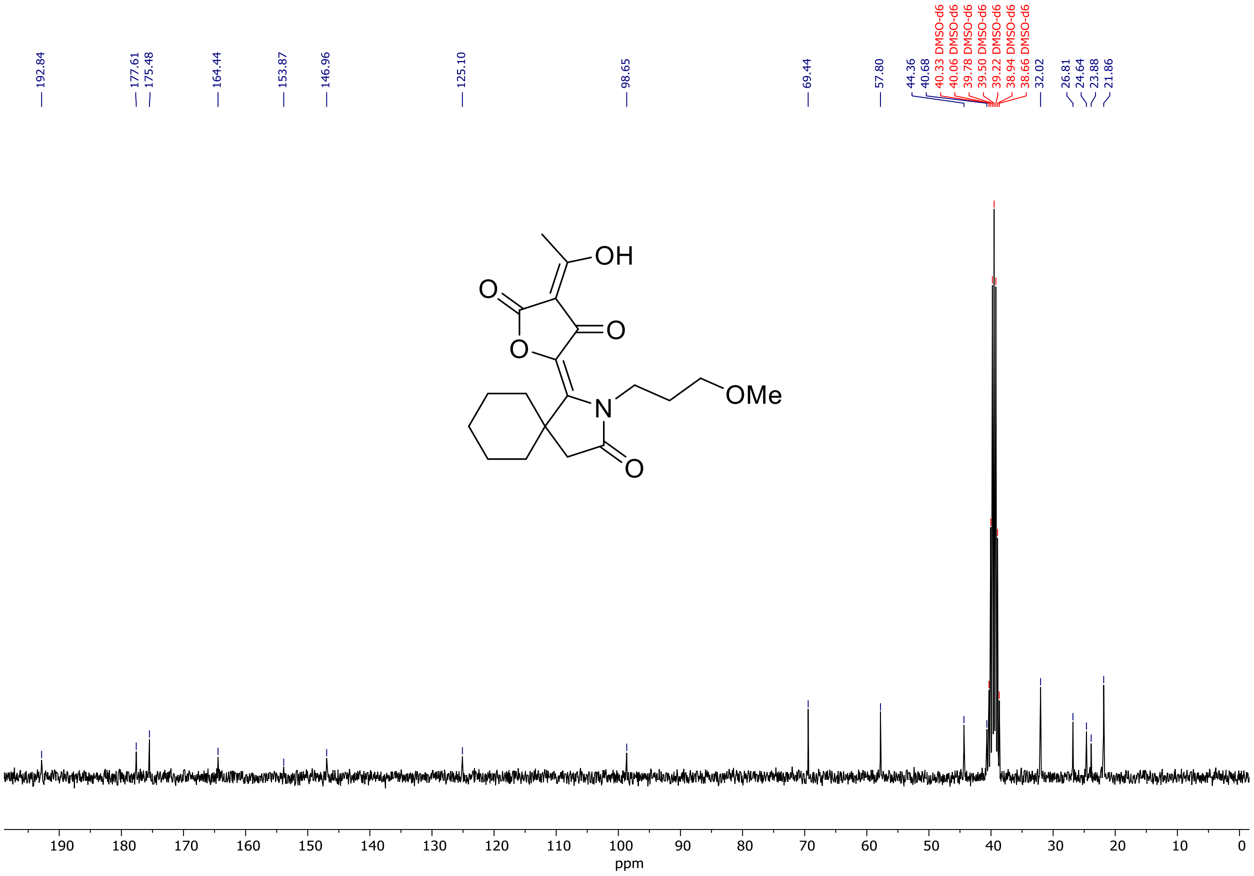

^{1}H NMR spectrum (300 MHz) of **4n** in DMSO-d_6

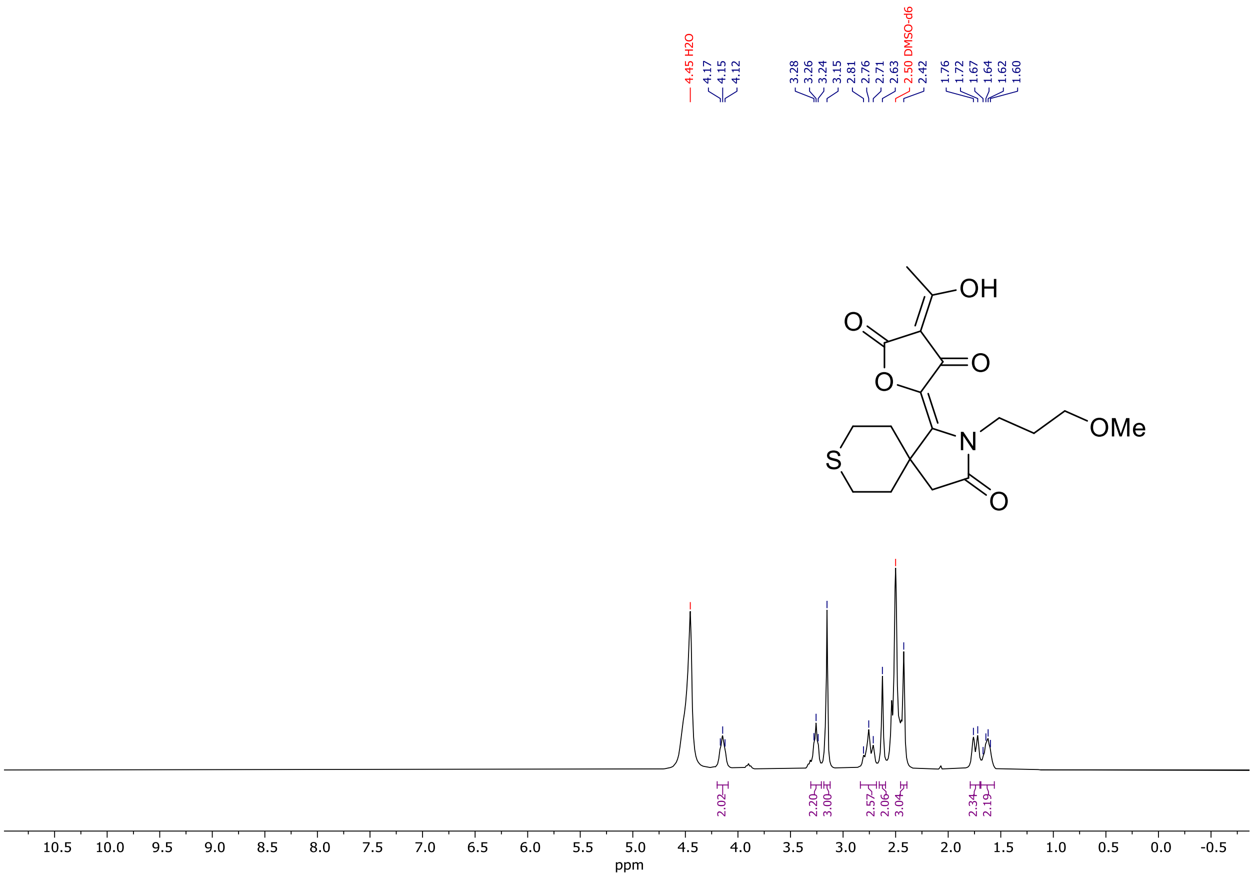

^{13}C {^{1}H} NMR spectrum (75 MHz) of **4n** in DMSO-d_6

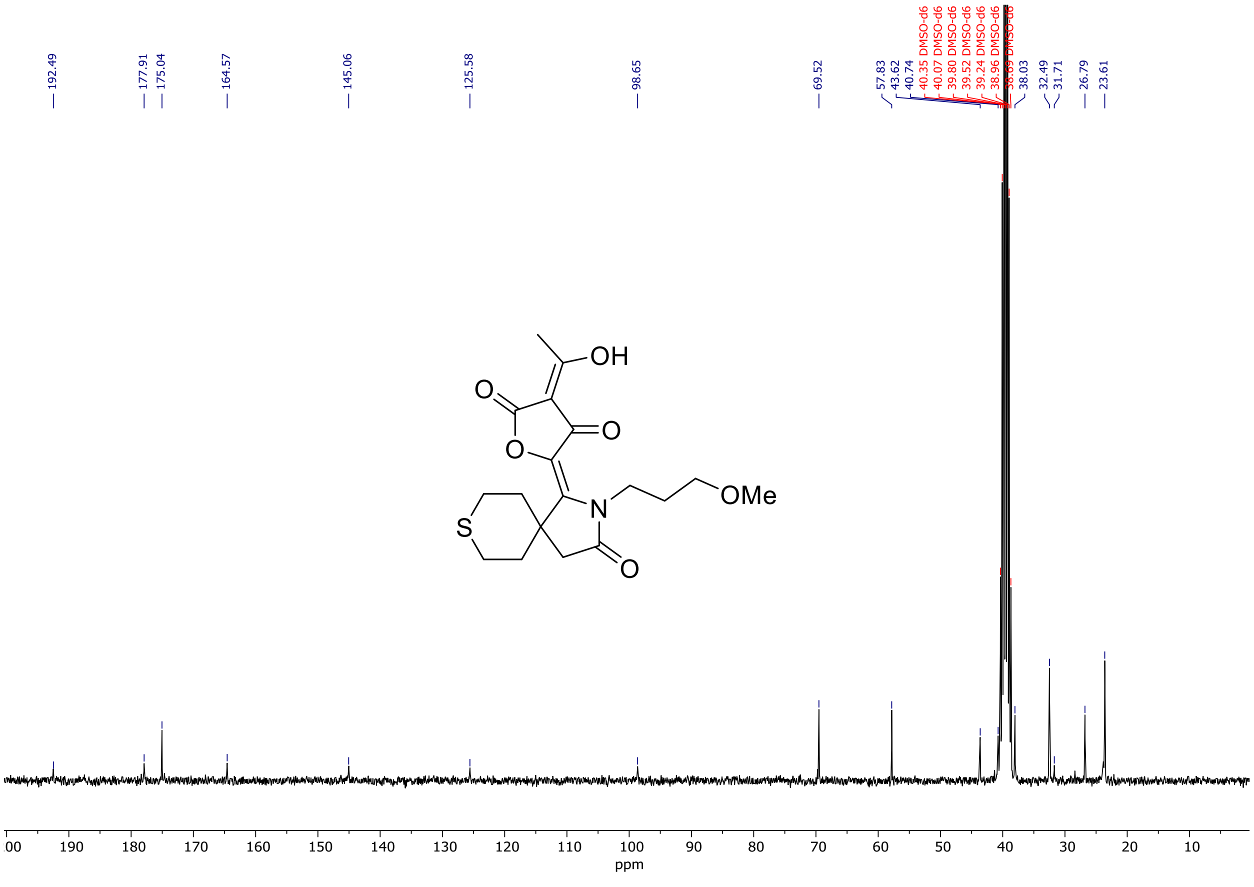

^{1}H NMR spectrum (300 MHz) of **4o** in DMSO-d_6

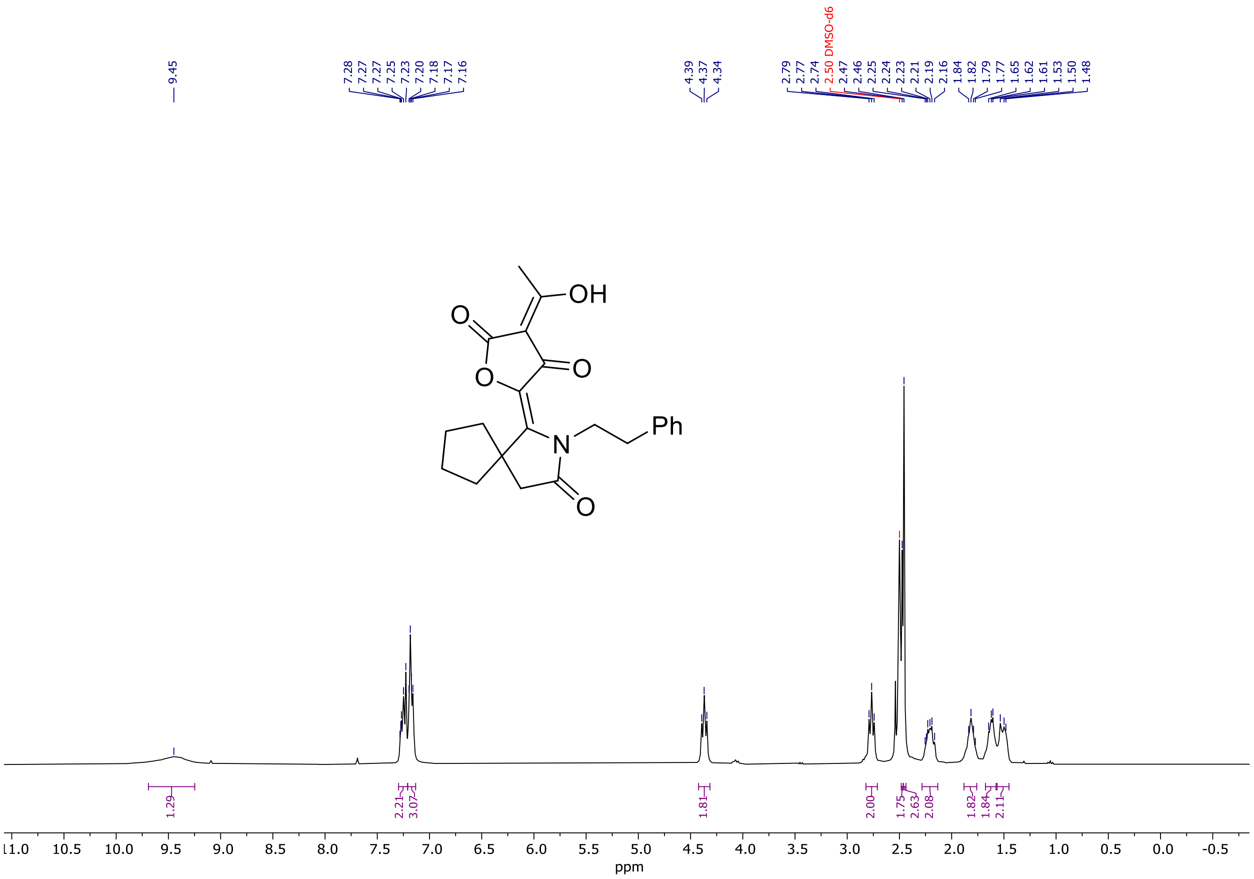

^{13}C {^{1}H} NMR spectrum (75 MHz) of **4o** in DMSO-d_6

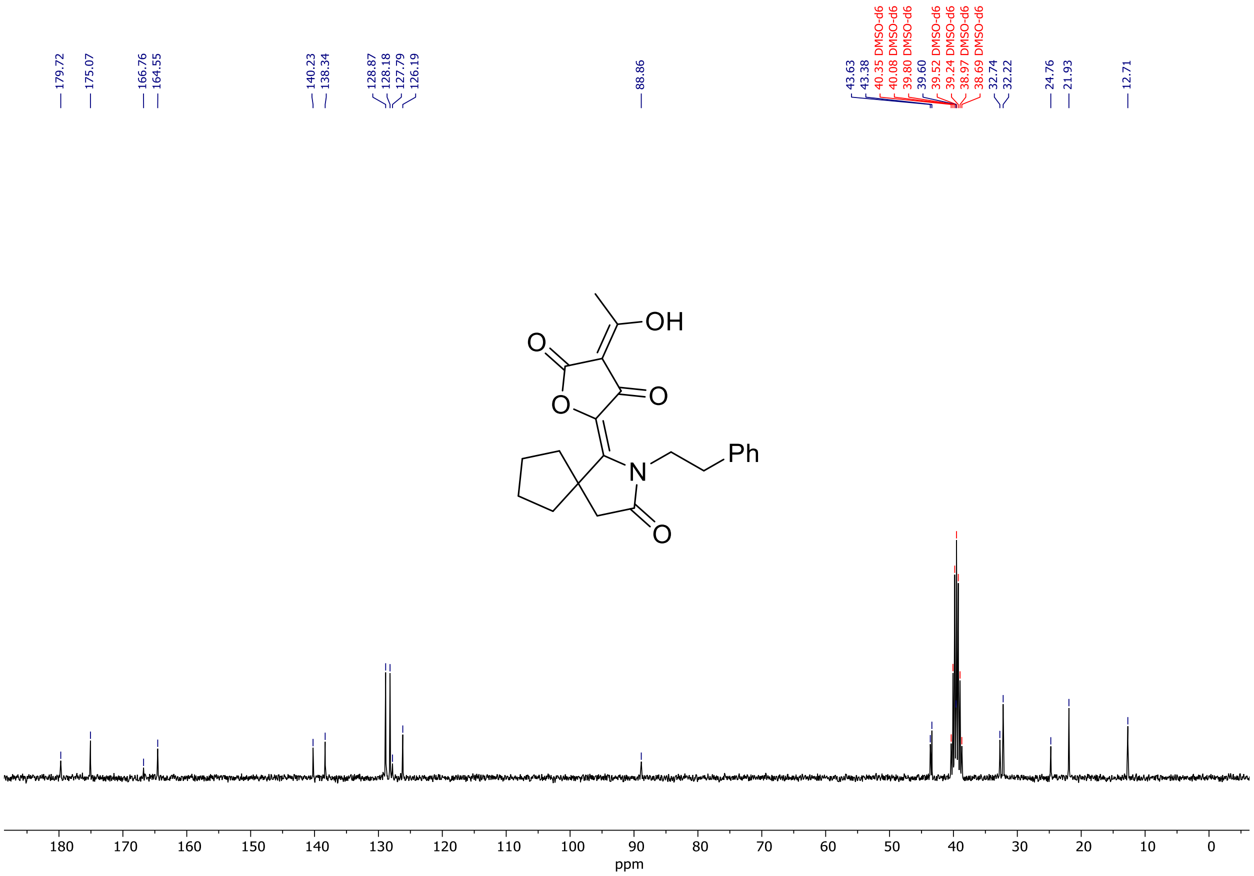

^{1}H NMR spectrum (300 MHz) of **4p** in DMSO-d_6

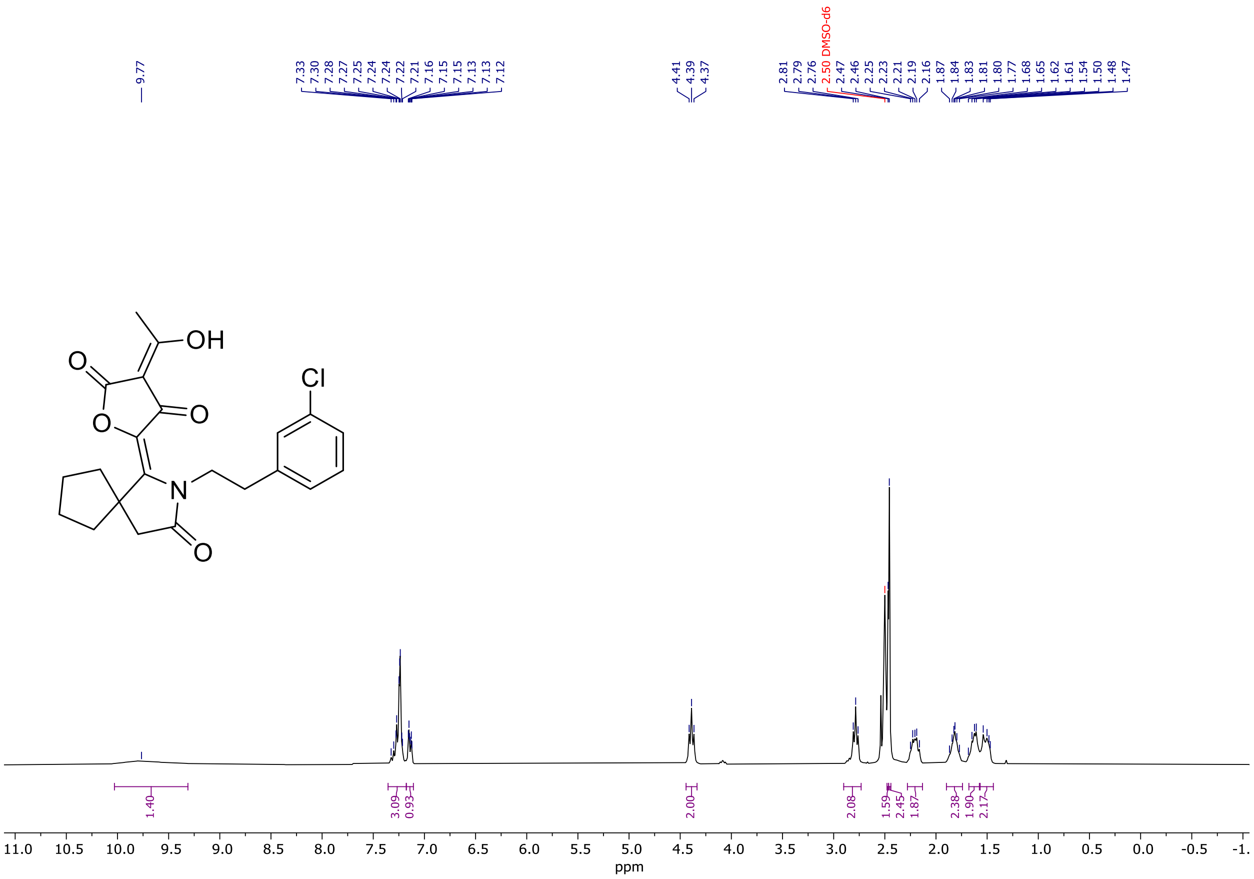

^{13}C {^{1}H} NMR spectrum (75 MHz) of **4p** in DMSO-d_6

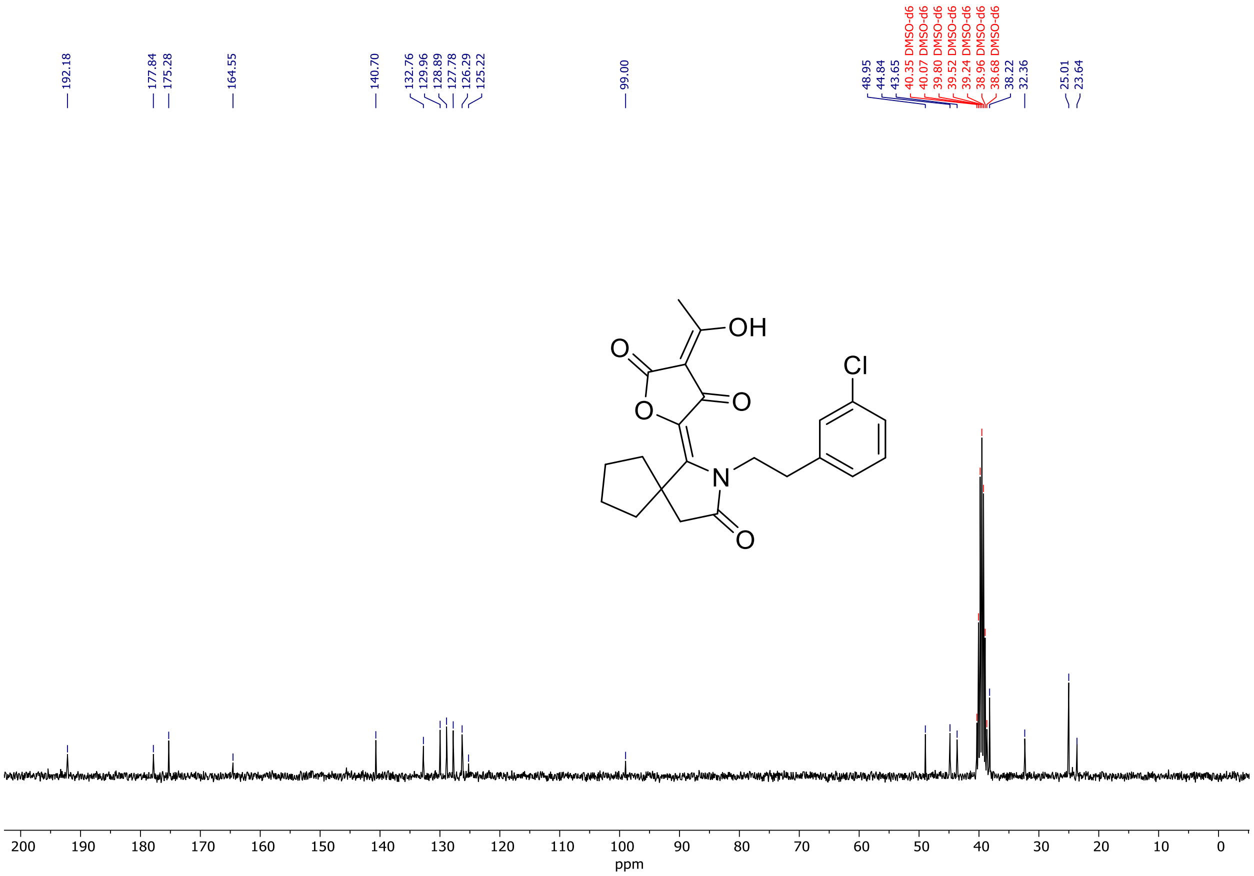

^{1}H NMR spectrum (300 MHz) of **4q** in DMSO-d_6

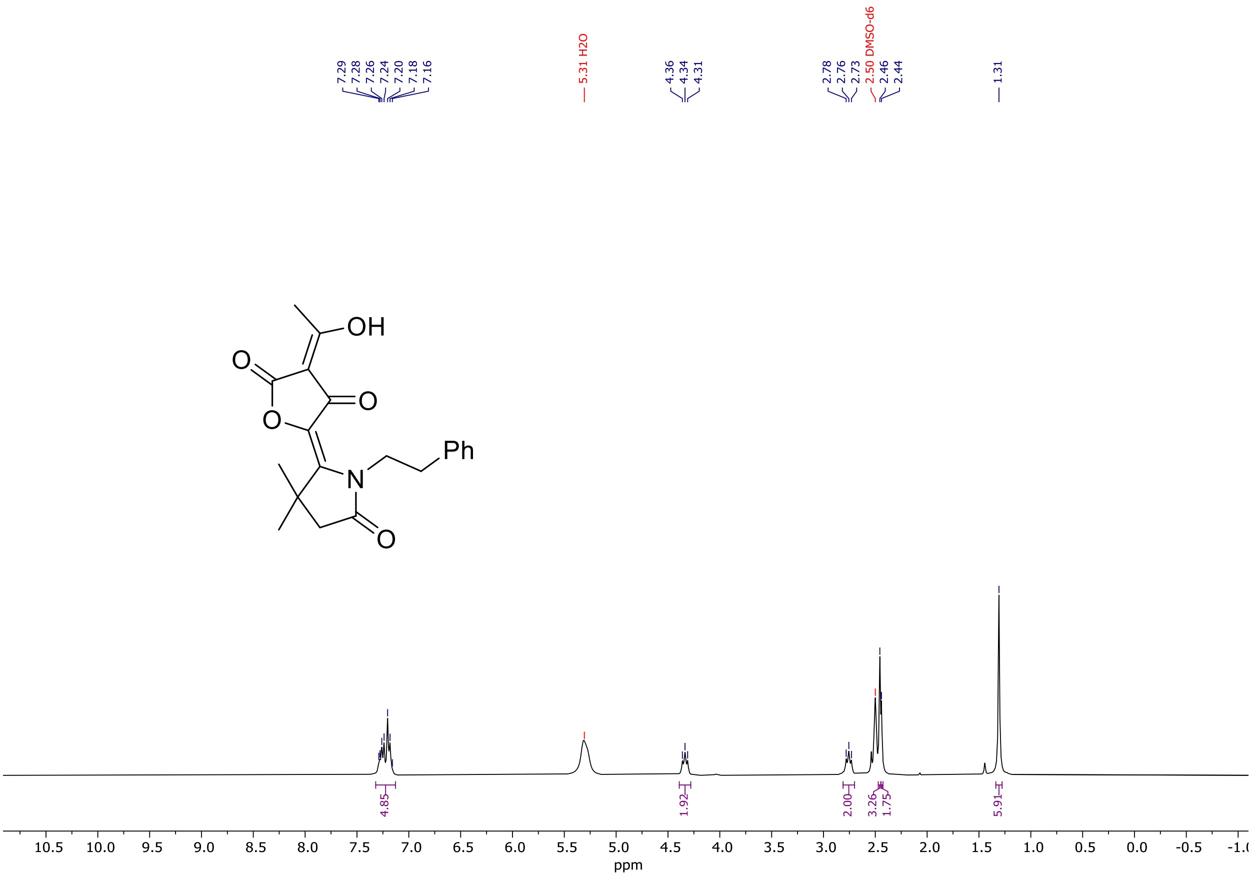

^{13}C {^{1}H} NMR spectrum (75 MHz) of **4q** in DMSO-d_6

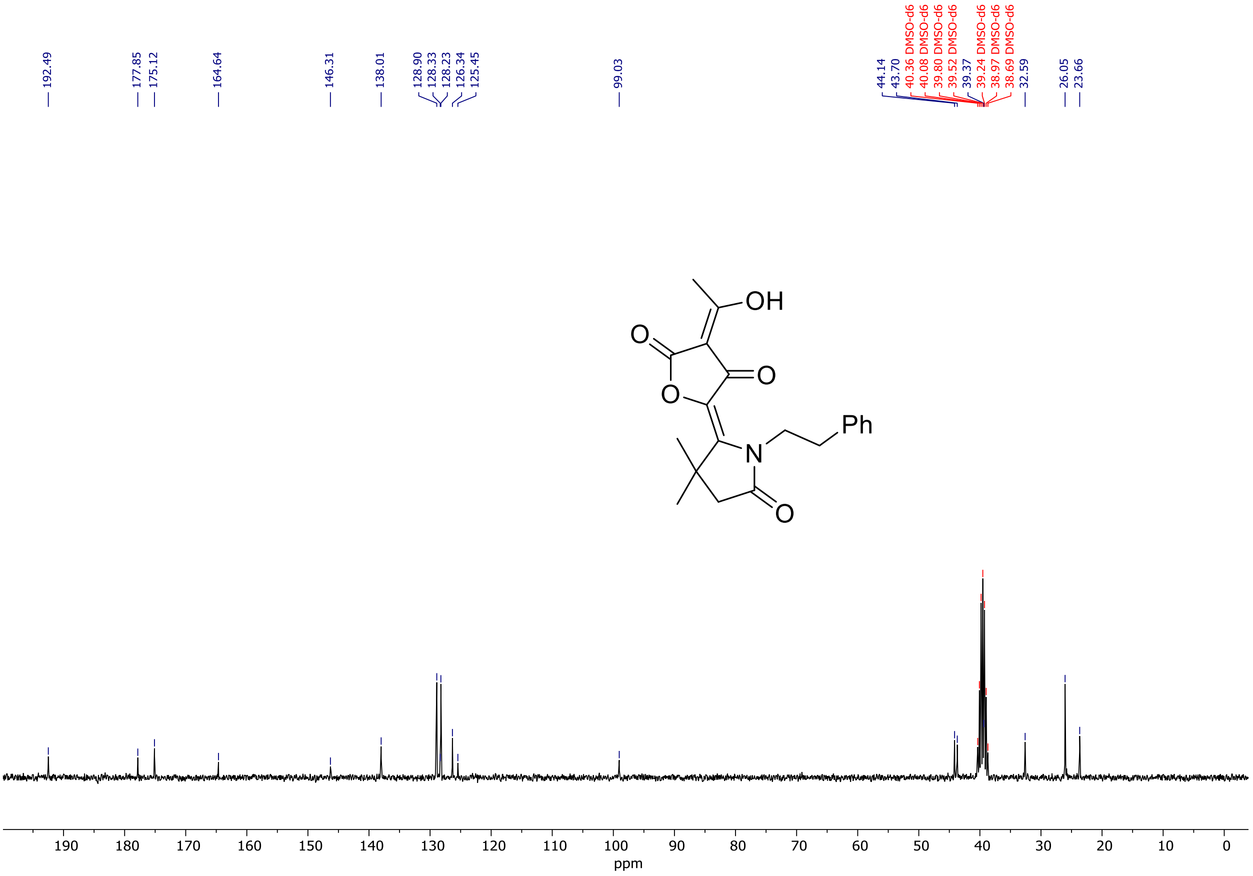

^{1}H NMR spectrum (300 MHz) of **4r** in DMSO-d_6

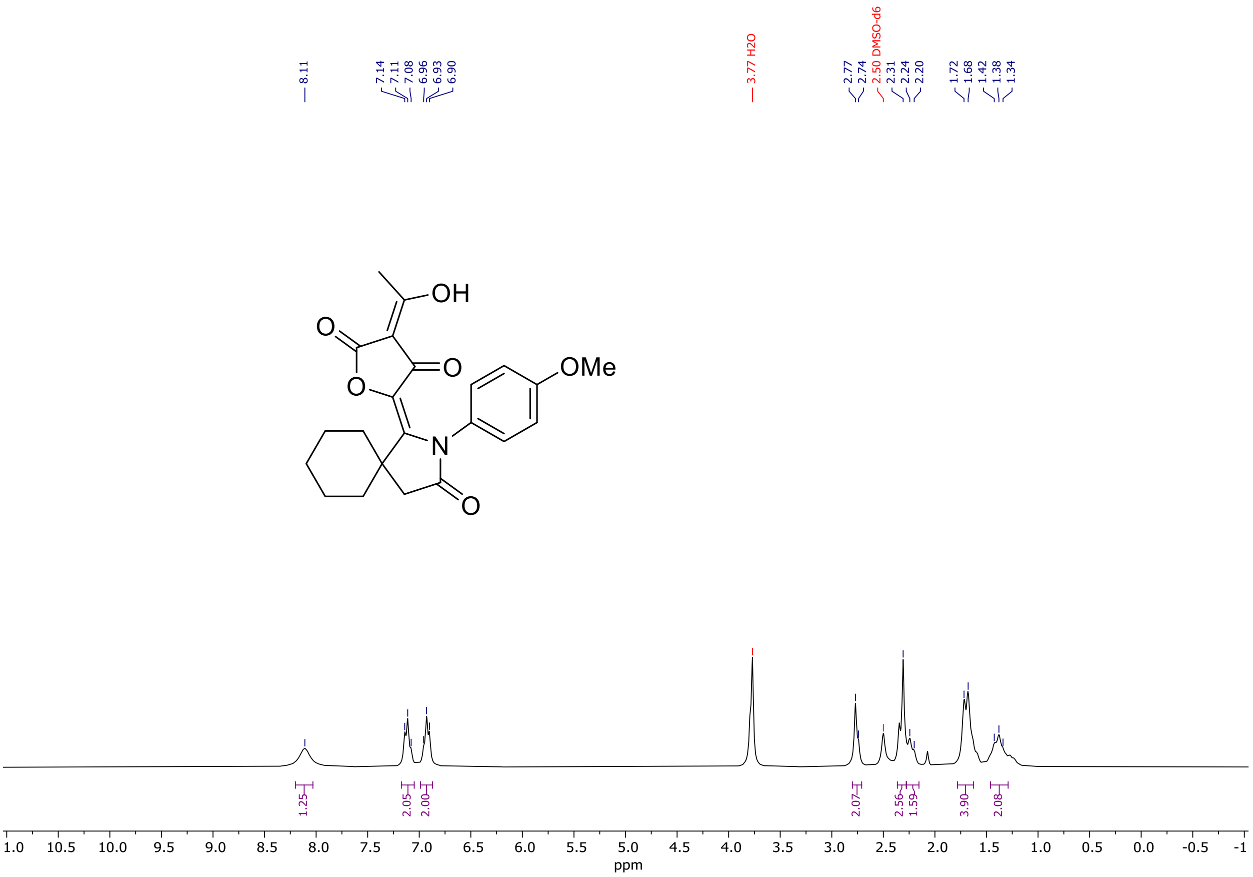

^{13}C {^{1}H} NMR spectrum (75 MHz) of **4r** in DMSO-d_6

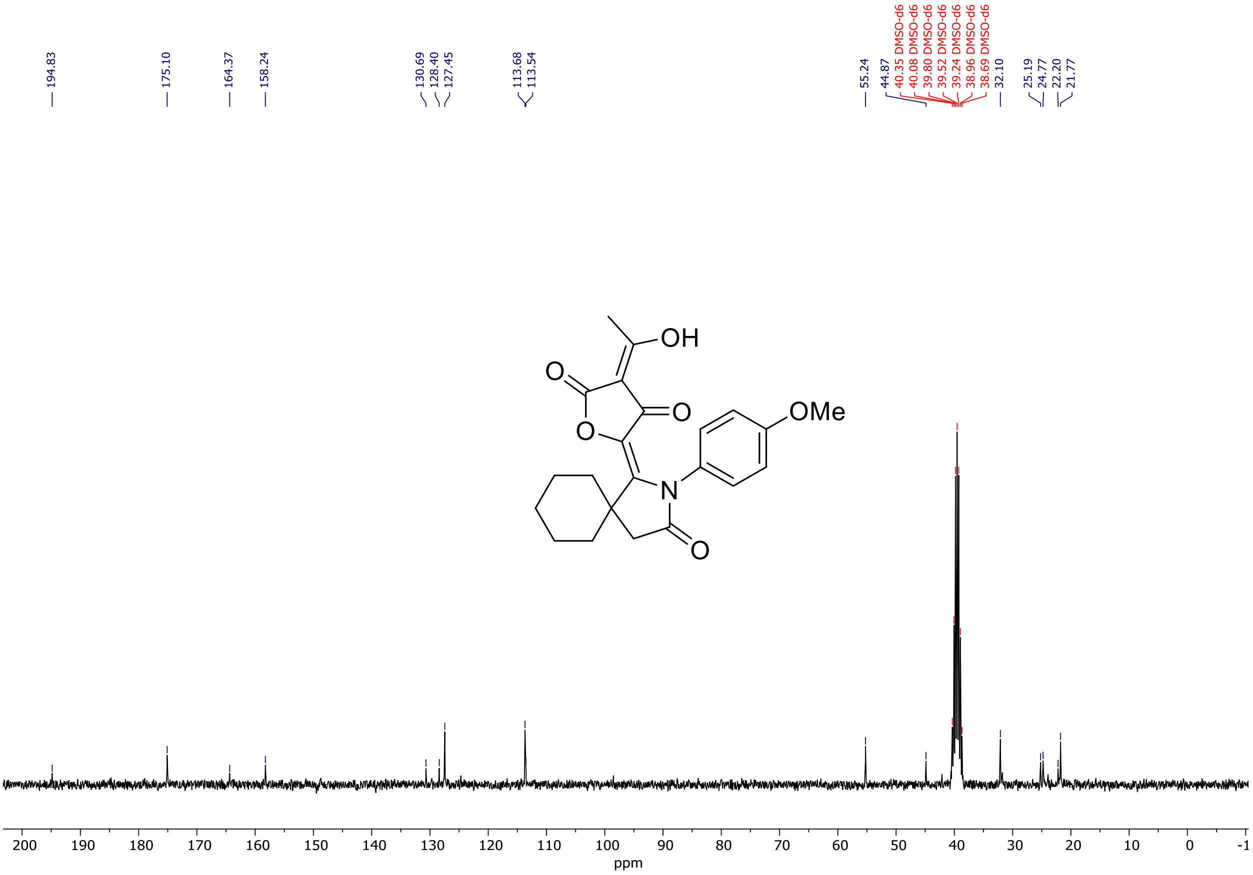

^{1}H NMR spectrum (300 MHz) of **4s** in DMSO-d_6

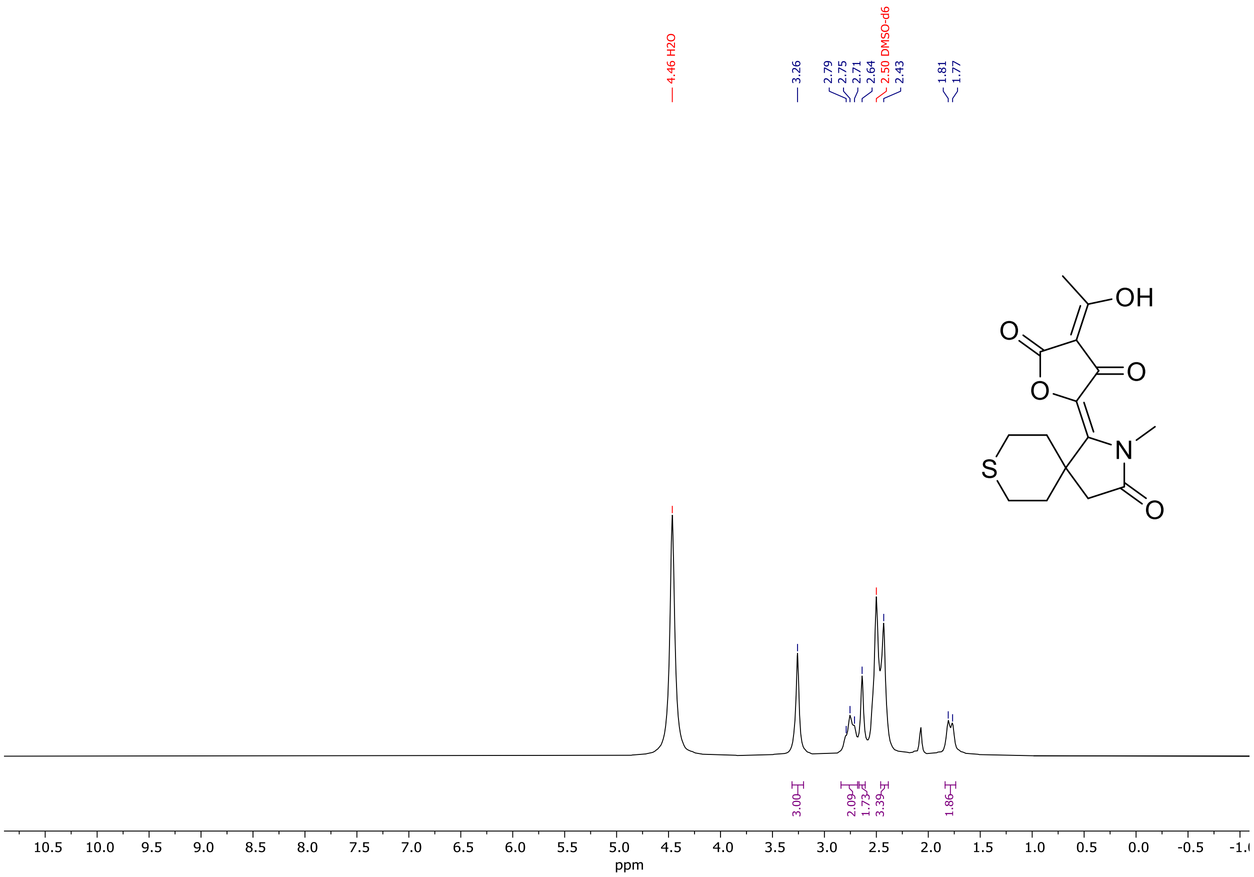

^{13}C {^{1}H} NMR spectrum (75 MHz) of **4s** in DMSO-d_6

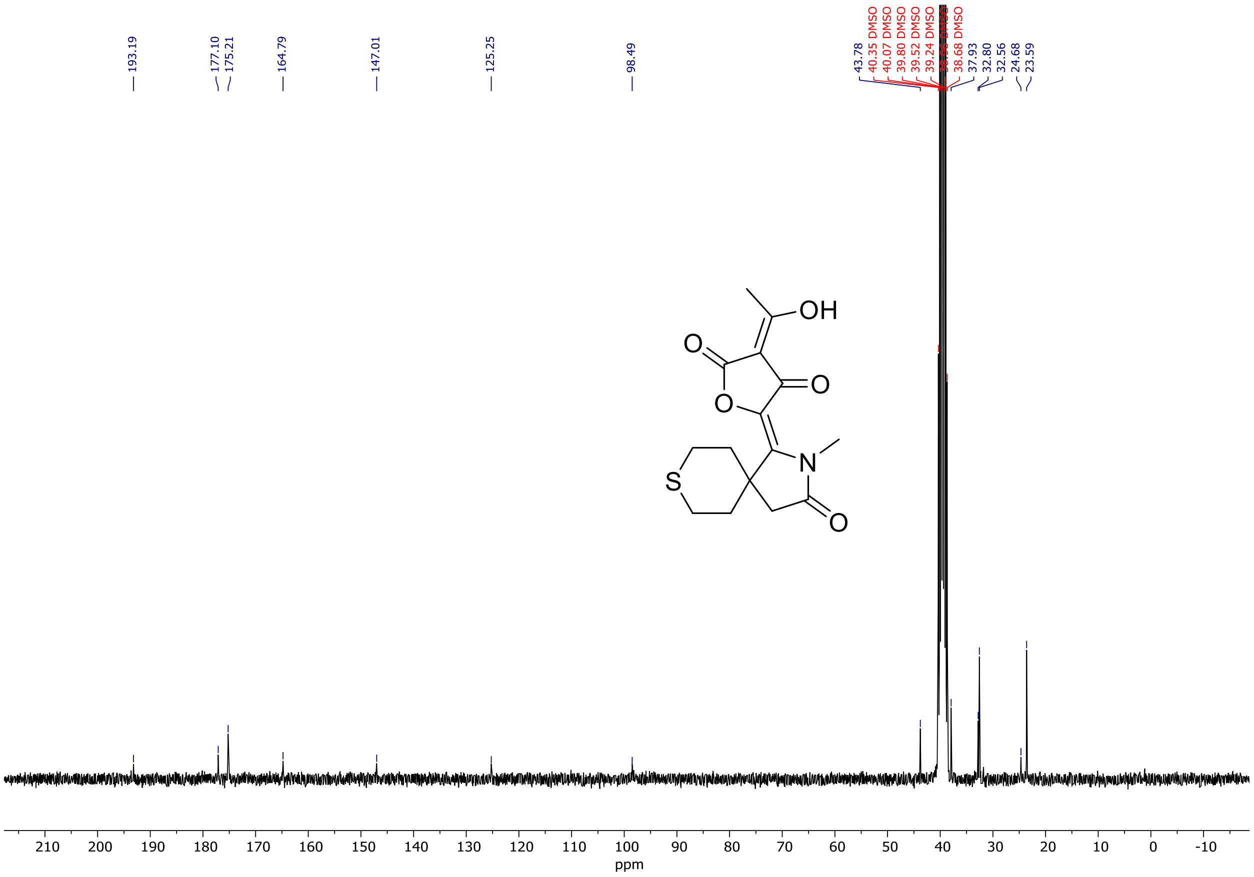

^{1}H NMR spectrum (300 MHz) of **4t** in DMSO-d_6

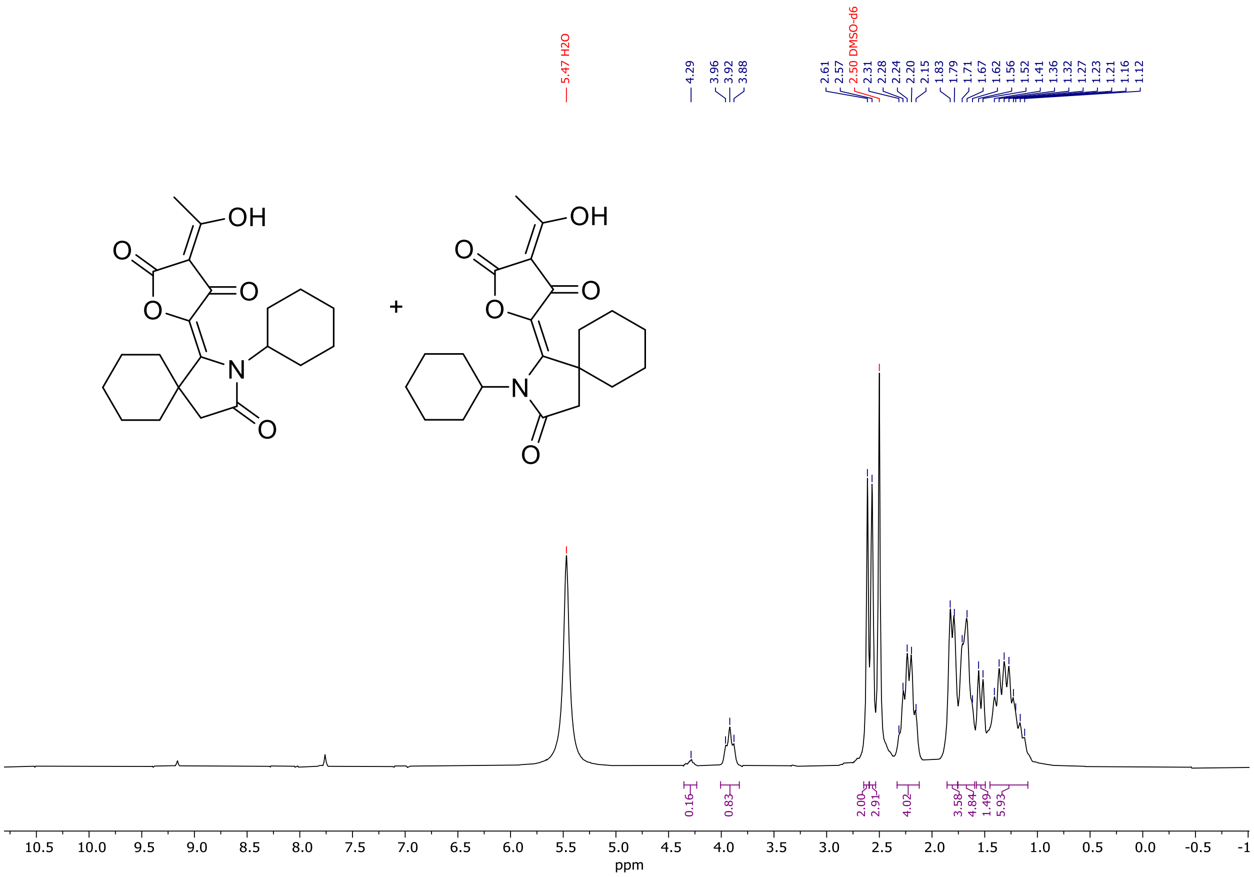

^{13}C {^{1}H} NMR spectrum (75 MHz) of **4t** in DMSO-d_6

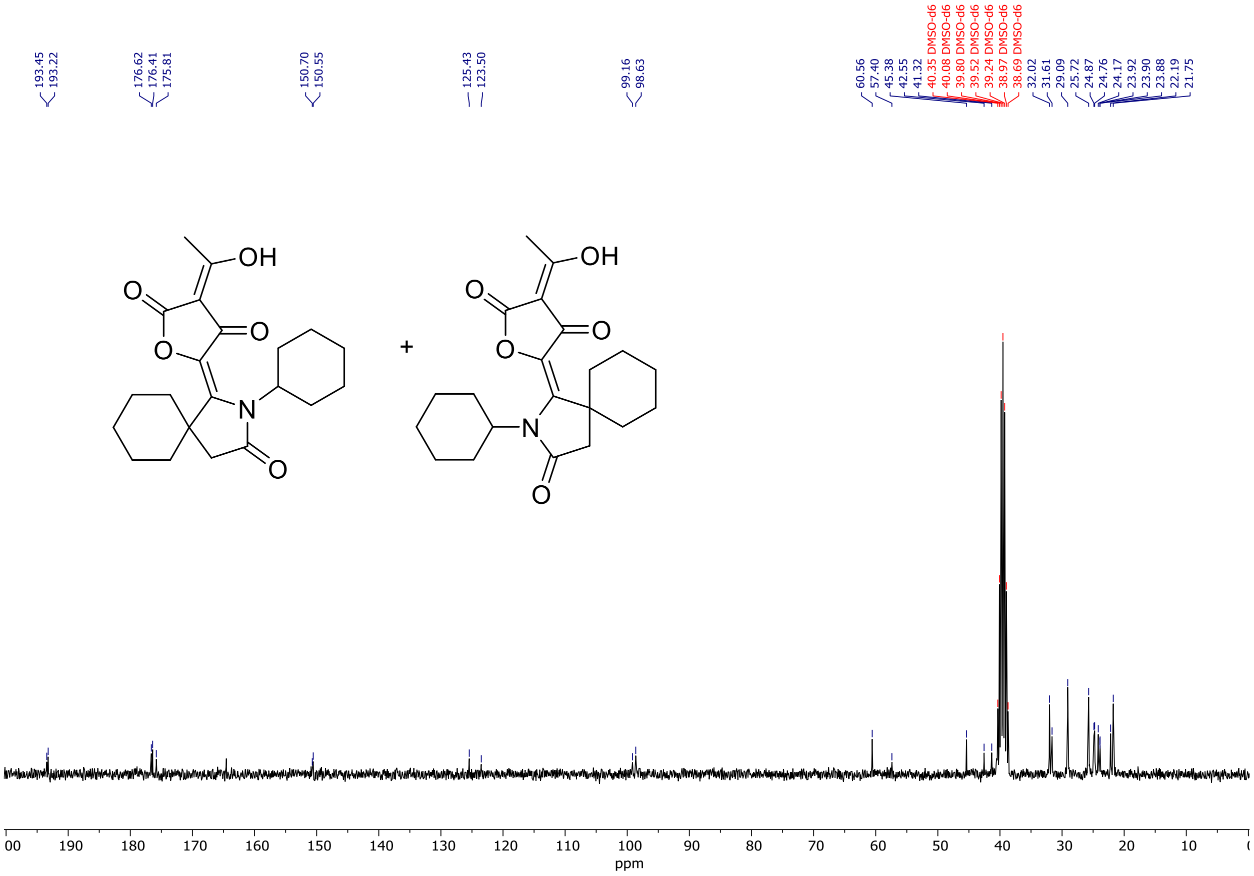

3. NMR ^{1}H and ^{13}C spectra for compounds 7 and 9

^{1}H NMR spectrum (300 MHz) of **7** in DMSO-d_6

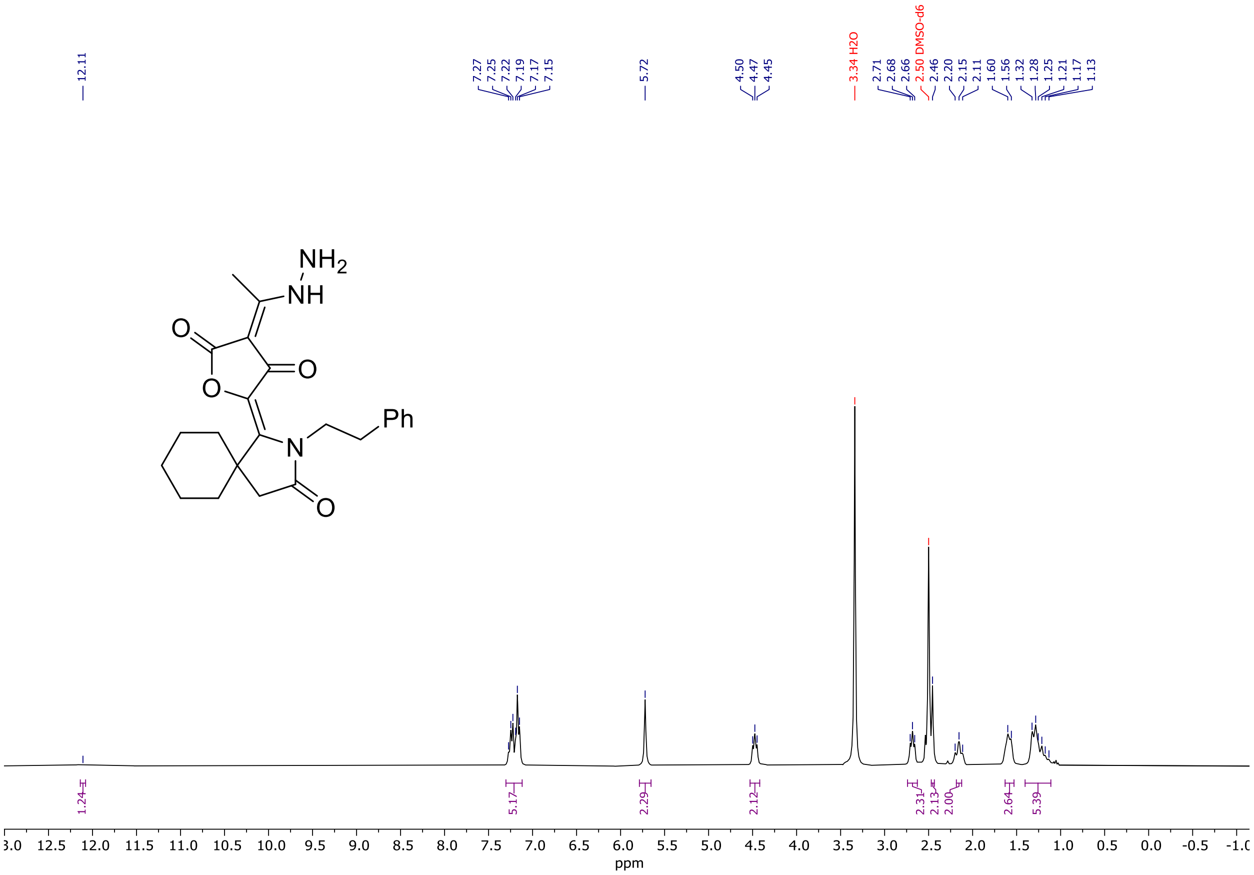

^{13}C {^{1}H} NMR spectrum (75 MHz) of **7** in DMSO-d_6

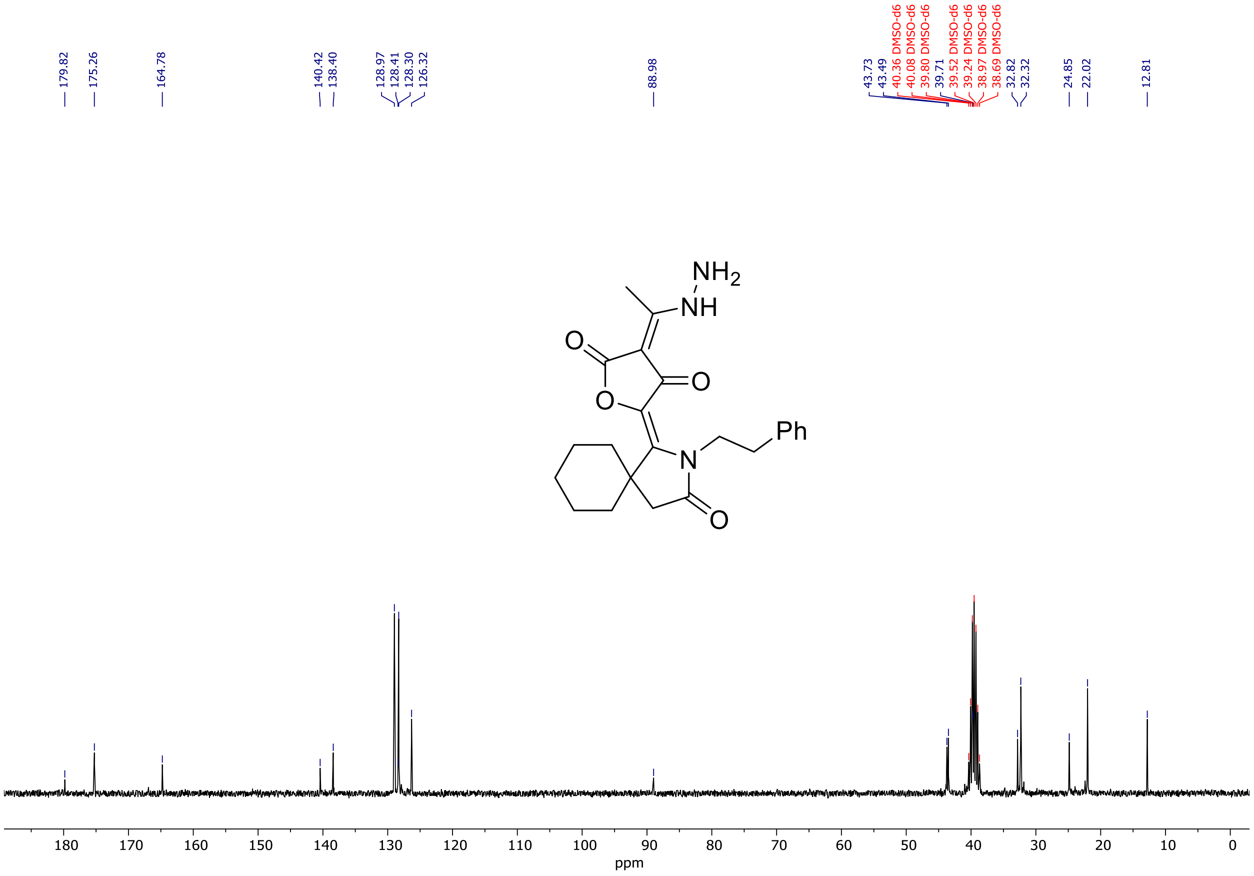

^{1}H NMR spectrum (300 MHz) of **9** in DMSO-d_6

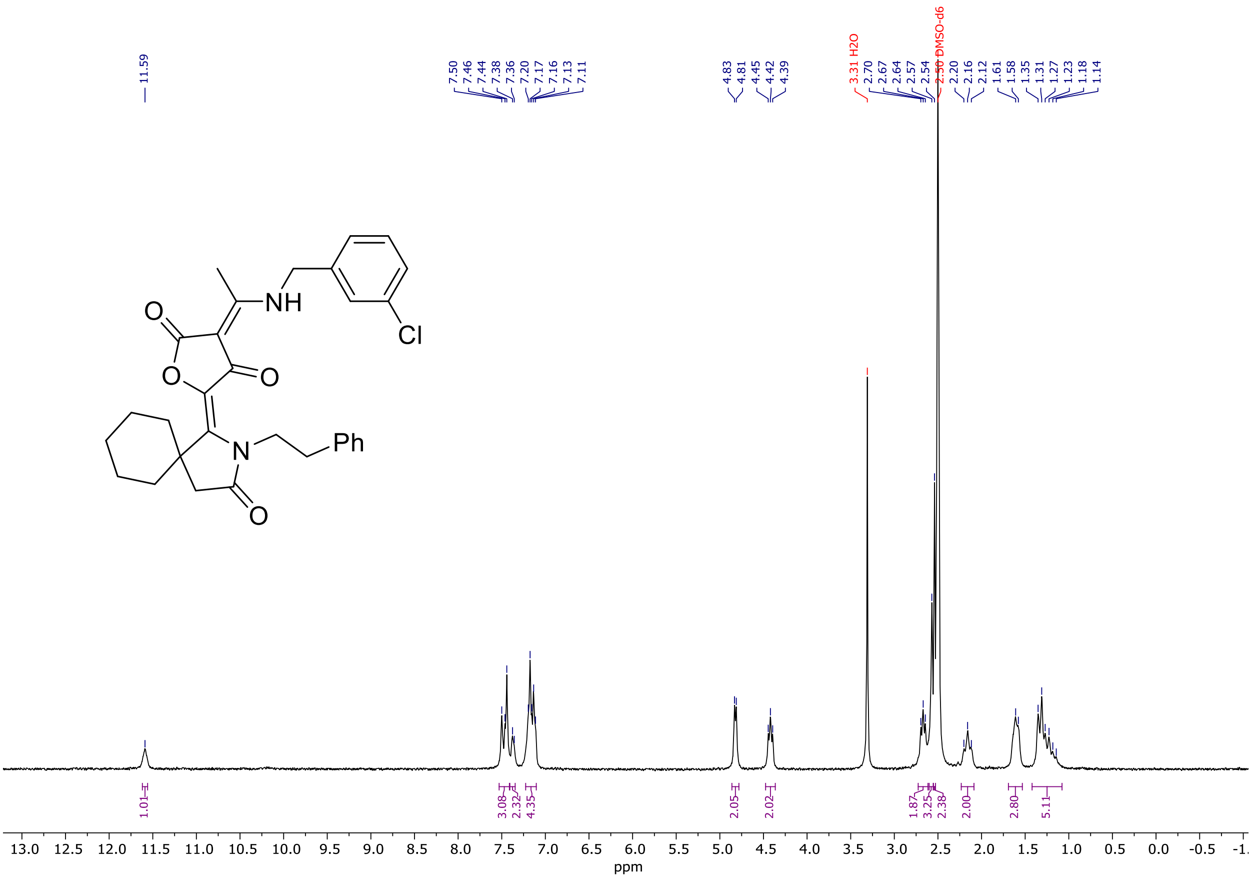

^{13}C {^{1}H} NMR spectrum (75 MHz) of **9** in DMSO-d_6

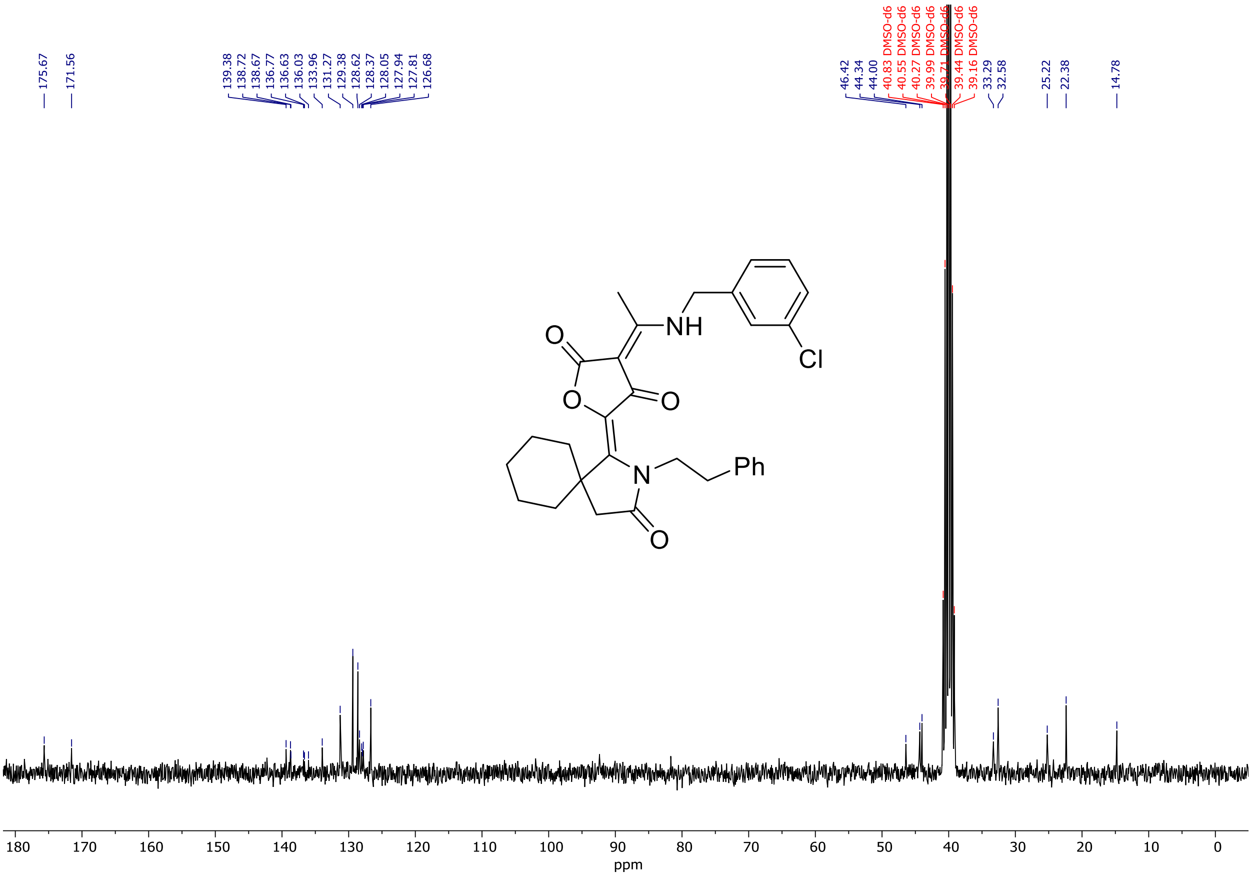

4. X-ray crystallographic data and refinement details

X-ray diffraction data were collected at 100K on a Rigaku Synergy S diffractometer equipped with a HyPix6000HE area-detector (kappa geometry, shutterless ω-scan technique), using monochromatized Cu K[illegible]-radiation. The intensity data were integrated and corrected for absorption and decay by the CrysAlisPro program[1]. The structure was solved by direct methods using SHELXT[2] and refined on F^2 using SHELXL-2018[3] in the OLEX2 program.[4] Positions of all atoms were found from the electron density-difference map. Atoms were refined with individual anisotropic (non-hydrogen atoms) or isotropic (hydrogen atoms) displacement parameters.

Acknowledgment

Crystal structure determination was performed in the Department of Structural Studies of Zelinsky Institute of Organic Chemistry, Moscow.

1. CrysAlisPro. Version 1.171.41. *Rigaku Oxford Diffraction*, **2021**.

2. Sheldrick, G. M. SHELXT - Integrated space-group and crystal-structure determination. *Acta Cryst.* **2015**, A71(1), 3-8. http://doi.org/10.1107/S2053273314026370

3. Sheldrick, G. M. Crystal structure refinement with SHELXL. *Acta Cryst.* **2015**, C71(1), 3-8. http://doi.org/10.1107/S2053229614024218

4. Dolomanov O.V.; Bourhis L.J.; Gildea R.J.; Howard J.A.K.; Puschmann H. OLEX2: a complete structure solution, refinement and analysis program. *J. Appl. Cryst.* **2009**, 42(2), 229-341. http://doi.org/10.1107/S0021889808042726

Crystallographic data for *(3E,5E)-3-(1-hydroxyethylidene)-5-(3-oxo-2-phenethyl-2-azaspiro[4.5]decan-1-ylidene)furan-2,4(3H,5H)-dione* **4a**

Table S1. Crystal data and structure refinement for 4a.

Identification code	2352876	
Empirical formula	$C_{23}H_{25}NO_5$	
Formula weight	395.44	
Temperature	100.0(3) K	
Wavelength	1.54184 Å	
Crystal system	Monoclinic	
Space group	P 1 21/c 1	
Unit cell dimensions	a = 11.39620(10) Å	α= 90°.
	b = 20.2036(2) Å	β= 102.1910(10)°.
	c = 8.96660(10) Å	γ = 90°.
Volume	2017.95(4) $Å^3$	
Z	4	
Density (calculated)	1.302 g/cm^3	
Absorption coefficient	0.749 mm^{-1}	
F(000)	840	
Crystal size	0.25 x 0.21 x 0.11 mm^3	
Theta range for data collection	3.968 to 78.110°.	
Index ranges	-14<=h<=14, -24<=k<=25, -11<=l<=11	
Reflections collected	26952	
Independent reflections	4316 [R(int) = 0.0285]	
Observed reflections	4078	
Completeness to theta = 67.684°	100.0 %	
Absorption correction	Semi-empirical from equivalents	
Max. and min. transmission	1.00000 and 0.75289	
Refinement method	Full-matrix least-squares on F^2	
Data / restraints / parameters	4316 / 0 / 267	
Goodness-of-fit on F^2	1.048	
Final R indices [I>2sigma(I)]	R1 = 0.0380, wR2 = 0.0957	
R indices (all data)	R1 = 0.0395, wR2 = 0.0967	
Largest diff. peak and hole	0.270 and -0.210 e.Å^{-3}	

Table S2. Atomic coordinates (x 10^4) and equivalent isotropic displacement parameters (Å^{2}x 10^3) for 4a. U(eq) is defined as one third of the trace of the orthogonalized U^{ij} tensor.

	x	y	z	U(eq)
O(1)	4948(1)	5071(1)	3248(1)	23(1)
O(2)	5746(1)	7793(1)	5321(1)	22(1)
O(3)	5847(1)	8864(1)	5963(1)	25(1)
O(4)	2272(1)	8763(1)	2826(1)	29(1)
O(5)	3031(1)	7576(1)	2591(1)	29(1)
N(1)	4660(1)	6175(1)	3696(1)	19(1)
C(1)	5339(1)	5597(1)	3792(1)	19(1)
C(2)	6582(1)	5746(1)	4657(1)	20(1)
C(3)	6551(1)	6461(1)	5249(1)	18(1)
C(4)	7619(1)	6863(1)	4923(1)	21(1)
C(5)	8811(1)	6610(1)	5883(1)	25(1)
C(6)	8805(1)	6627(1)	7588(2)	29(1)
C(7)	7753(1)	6230(1)	7934(1)	26(1)
C(8)	6552(1)	6464(1)	6977(1)	22(1)
C(9)	5321(1)	6708(1)	4380(1)	18(1)
C(10)	4954(1)	7350(1)	4363(1)	20(1)
C(11)	3922(1)	7740(1)	3602(1)	22(1)
C(12)	4122(1)	8399(1)	4205(1)	21(1)
C(13)	3282(1)	8897(1)	3802(1)	22(1)
C(14)	3414(1)	9584(1)	4368(1)	26(1)
C(15)	5280(1)	8422(1)	5240(1)	21(1)
C(16)	3369(1)	6144(1)	3046(1)	21(1)
C(17)	2623(1)	6203(1)	4276(1)	21(1)
C(18)	1315(1)	6093(1)	3553(1)	20(1)
C(19)	629(1)	6597(1)	2735(1)	24(1)
C(20)	-552(1)	6483(1)	1992(1)	26(1)
C(21)	-1067(1)	5865(1)	2056(1)	24(1)
C(22)	-397(1)	5359(1)	2867(1)	26(1)
C(23)	784(1)	5475(1)	3611(1)	24(1)

Table S3. Bond lengths [Å] and angles [°] for 4a.

O(1)-C(1)	1.2149(14)
O(2)-C(10)	1.4236(13)
O(2)-C(15)	1.3738(13)
O(3)-C(15)	1.2078(14)
O(4)-H(4)	1.051(16)
O(4)-C(13)	1.3188(15)
O(5)-C(11)	1.2549(14)
N(1)-C(1)	1.3929(14)
N(1)-C(9)	1.3818(14)
N(1)-C(16)	1.4650(14)
C(1)-C(2)	1.4948(15)
C(2)-H(2A)	0.9900
C(2)-H(2B)	0.9900
C(2)-C(3)	1.5426(15)
C(3)-C(4)	1.5418(15)
C(3)-C(8)	1.5487(15)
C(3)-C(9)	1.5360(15)
C(4)-H(4A)	0.9900
C(4)-H(4B)	0.9900
C(4)-C(5)	1.5338(16)
C(5)-H(5A)	0.9900
C(5)-H(5B)	0.9900
C(5)-C(6)	1.5309(18)
C(6)-H(6A)	0.9900
C(6)-H(6B)	0.9900
C(6)-C(7)	1.5281(18)
C(7)-H(7A)	0.9900
C(7)-H(7B)	0.9900
C(7)-C(8)	1.5289(16)
C(8)-H(8A)	0.9900
C(8)-H(8B)	0.9900
C(9)-C(10)	1.3614(16)
C(10)-C(11)	1.4595(16)
C(11)-C(12)	1.4369(16)
C(12)-C(13)	1.3823(16)
C(12)-C(15)	1.4452(16)
C(13)-C(14)	1.4737(16)
C(14)-H(14A)	0.9800
C(14)-H(14B)	0.9800
C(14)-H(14C)	0.9800
C(16)-H(16A)	0.9900
C(16)-H(16B)	0.9900
C(16)-C(17)	1.5331(15)
C(17)-H(17A)	0.9900
C(17)-H(17B)	0.9900
C(17)-C(18)	1.5106(15)
C(18)-C(19)	1.3943(16)
C(18)-C(23)	1.3939(16)
C(19)-H(19)	0.9500
C(19)-C(20)	1.3887(16)
C(20)-H(20)	0.9500

C(20)-C(21)	1.3870(17)
C(21)-H(21)	0.9500
C(21)-C(22)	1.3857(17)
C(22)-H(22)	0.9500
C(22)-C(23)	1.3901(16)
C(23)-H(23)	0.9500

C(15)-O(2)-C(10)	111.15(9)
C(13)-O(4)-H(4)	104.2(9)
C(1)-N(1)-C(16)	119.16(9)
C(9)-N(1)-C(1)	112.49(9)
C(9)-N(1)-C(16)	128.09(9)
O(1)-C(1)-N(1)	123.86(10)
O(1)-C(1)-C(2)	127.66(10)
N(1)-C(1)-C(2)	108.48(9)
C(1)-C(2)-H(2A)	110.5
C(1)-C(2)-H(2B)	110.5
C(1)-C(2)-C(3)	106.15(9)
H(2A)-C(2)-H(2B)	108.7
C(3)-C(2)-H(2A)	110.5
C(3)-C(2)-H(2B)	110.5
C(2)-C(3)-C(8)	110.69(9)
C(4)-C(3)-C(2)	110.55(9)
C(4)-C(3)-C(8)	110.71(9)
C(9)-C(3)-C(2)	102.43(8)
C(9)-C(3)-C(4)	113.87(9)
C(9)-C(3)-C(8)	108.29(9)
C(3)-C(4)-H(4A)	109.4
C(3)-C(4)-H(4B)	109.4
H(4A)-C(4)-H(4B)	108.0
C(5)-C(4)-C(3)	111.06(9)
C(5)-C(4)-H(4A)	109.4
C(5)-C(4)-H(4B)	109.4
C(4)-C(5)-H(5A)	109.4
C(4)-C(5)-H(5B)	109.4
H(5A)-C(5)-H(5B)	108.0
C(6)-C(5)-C(4)	111.28(10)
C(6)-C(5)-H(5A)	109.4
C(6)-C(5)-H(5B)	109.4
C(5)-C(6)-H(6A)	109.4
C(5)-C(6)-H(6B)	109.4
H(6A)-C(6)-H(6B)	108.0
C(7)-C(6)-C(5)	111.13(10)
C(7)-C(6)-H(6A)	109.4
C(7)-C(6)-H(6B)	109.4
C(6)-C(7)-H(7A)	109.3
C(6)-C(7)-H(7B)	109.3
C(6)-C(7)-C(8)	111.78(10)
H(7A)-C(7)-H(7B)	107.9
C(8)-C(7)-H(7A)	109.3
C(8)-C(7)-H(7B)	109.3
C(3)-C(8)-H(8A)	109.2
C(3)-C(8)-H(8B)	109.2

C(7)-C(8)-C(3)	111.84(9)
C(7)-C(8)-H(8A)	109.2
C(7)-C(8)-H(8B)	109.2
H(8A)-C(8)-H(8B)	107.9
N(1)-C(9)-C(3)	108.95(9)
C(10)-C(9)-N(1)	126.89(10)
C(10)-C(9)-C(3)	124.11(10)
O(2)-C(10)-C(11)	106.75(9)
C(9)-C(10)-O(2)	115.95(10)
C(9)-C(10)-C(11)	137.30(11)
O(5)-C(11)-C(10)	130.14(11)
O(5)-C(11)-C(12)	123.76(11)
C(12)-C(11)-C(10)	106.07(10)
C(11)-C(12)-C(15)	108.78(10)
C(13)-C(12)-C(11)	122.01(11)
C(13)-C(12)-C(15)	129.21(11)
O(4)-C(13)-C(12)	118.88(11)
O(4)-C(13)-C(14)	115.67(10)
C(12)-C(13)-C(14)	125.45(11)
C(13)-C(14)-H(14A)	109.5
C(13)-C(14)-H(14B)	109.5
C(13)-C(14)-H(14C)	109.5
H(14A)-C(14)-H(14B)	109.5
H(14A)-C(14)-H(14C)	109.5
H(14B)-C(14)-H(14C)	109.5
O(2)-C(15)-C(12)	107.15(9)
O(3)-C(15)-O(2)	119.91(10)
O(3)-C(15)-C(12)	132.94(11)
N(1)-C(16)-H(16A)	109.2
N(1)-C(16)-H(16B)	109.2
N(1)-C(16)-C(17)	111.95(9)
H(16A)-C(16)-H(16B)	107.9
C(17)-C(16)-H(16A)	109.2
C(17)-C(16)-H(16B)	109.2
C(16)-C(17)-H(17A)	109.9
C(16)-C(17)-H(17B)	109.9
H(17A)-C(17)-H(17B)	108.3
C(18)-C(17)-C(16)	109.06(9)
C(18)-C(17)-H(17A)	109.9
C(18)-C(17)-H(17B)	109.9
C(19)-C(18)-C(17)	120.80(10)
C(23)-C(18)-C(17)	120.78(10)
C(23)-C(18)-C(19)	118.31(11)
C(18)-C(19)-H(19)	119.7
C(20)-C(19)-C(18)	120.67(11)
C(20)-C(19)-H(19)	119.7
C(19)-C(20)-H(20)	119.8
C(21)-C(20)-C(19)	120.37(11)
C(21)-C(20)-H(20)	119.8
C(20)-C(21)-H(21)	120.2
C(22)-C(21)-C(20)	119.63(11)
C(22)-C(21)-H(21)	120.2
C(21)-C(22)-H(22)	120.1

C(21)-C(22)-C(23)	119.87(11)
C(23)-C(22)-H(22)	120.1
C(18)-C(23)-H(23)	119.4
C(22)-C(23)-C(18)	121.14(11)
C(22)-C(23)-H(23)	119.4

Table S4. Anisotropic displacement parameters ($Å^2 x 10^3$) for 4a. The anisotropic displacement factor exponent takes the form: $-2p^2[h^2 a^{*2}U^{11} + ... + 2 h k a^* b^* U^{12}]$

	U^{11}	U^{22}	U^{33}	U^{23}	U^{13}	U^{12}
O(1)	28(1)	18(1)	24(1)	-3(1)	7(1)	-4(1)
O(2)	21(1)	16(1)	27(1)	-4(1)	3(1)	1(1)
O(3)	26(1)	20(1)	30(1)	-5(1)	6(1)	-2(1)
O(4)	26(1)	27(1)	31(1)	4(1)	1(1)	3(1)
O(5)	26(1)	24(1)	32(1)	1(1)	-4(1)	-2(1)
N(1)	18(1)	18(1)	22(1)	-3(1)	5(1)	-1(1)
C(1)	22(1)	18(1)	19(1)	0(1)	8(1)	-1(1)
C(2)	20(1)	16(1)	25(1)	-1(1)	6(1)	0(1)
C(3)	18(1)	16(1)	21(1)	-1(1)	5(1)	0(1)
C(4)	20(1)	17(1)	26(1)	0(1)	7(1)	-1(1)
C(5)	19(1)	22(1)	33(1)	-2(1)	5(1)	0(1)
C(6)	23(1)	29(1)	30(1)	-4(1)	-1(1)	2(1)
C(7)	29(1)	27(1)	23(1)	1(1)	2(1)	3(1)
C(8)	23(1)	22(1)	21(1)	-1(1)	6(1)	1(1)
C(9)	19(1)	19(1)	18(1)	-1(1)	6(1)	-1(1)
C(10)	20(1)	19(1)	22(1)	-2(1)	4(1)	-2(1)
C(11)	22(1)	20(1)	24(1)	2(1)	5(1)	-1(1)
C(12)	22(1)	19(1)	23(1)	1(1)	7(1)	0(1)
C(13)	23(1)	22(1)	22(1)	4(1)	8(1)	1(1)
C(14)	30(1)	20(1)	30(1)	4(1)	9(1)	4(1)
C(15)	22(1)	18(1)	24(1)	0(1)	9(1)	1(1)
C(16)	19(1)	22(1)	20(1)	-3(1)	4(1)	-2(1)
C(17)	20(1)	24(1)	20(1)	-1(1)	4(1)	0(1)
C(18)	20(1)	22(1)	20(1)	-1(1)	6(1)	1(1)
C(19)	24(1)	20(1)	29(1)	2(1)	5(1)	-2(1)
C(20)	24(1)	23(1)	28(1)	4(1)	2(1)	3(1)
C(21)	19(1)	28(1)	25(1)	0(1)	4(1)	0(1)
C(22)	24(1)	22(1)	32(1)	2(1)	7(1)	-3(1)
C(23)	22(1)	21(1)	28(1)	4(1)	5(1)	3(1)

Table S5. Hydrogen coordinates (x 10^4) and isotropic displacement parameters (Å^2 x 10^3) for 4a.

	x	y	z	U(eq)
H(4)	2345(14)	8259(8)	2569(18)	30(4)
H(2A)	7164	5708	3982	24
H(2B)	6820	5434	5519	24
H(4A)	7636	6827	3826	25
H(4B)	7515	7335	5158	25
H(5A)	8950	6151	5578	30
H(5B)	9477	6889	5684	30
H(6A)	9568	6441	8173	35
H(6B)	8746	7092	7916	35
H(7A)	7740	6275	9030	32
H(7B)	7866	5756	7724	32
H(8A)	6385	6919	7292	26
H(8B)	5903	6172	7170	26
H(14A)	3611	9872	3576	39
H(14B)	4060	9605	5282	39
H(14C)	2660	9730	4620	39
H(16A)	3180	5720	2497	25
H(16B)	3148	6508	2299	25
H(17A)	2892	5868	5083	25
H(17B)	2732	6647	4750	25
H(19)	973	7023	2685	29
H(20)	-1009	6831	1437	31
H(21)	-1875	5788	1548	29
H(22)	-745	4934	2914	31
H(23)	1237	5127	4168	29

Table S6. Torsion angles [°] for 4a.

O(1)-C(1)-C(2)-C(3)	-173.26(11)
O(2)-C(10)-C(11)-O(5)	175.25(11)
O(2)-C(10)-C(11)-C(12)	-2.78(12)
O(5)-C(11)-C(12)-C(13)	5.69(18)
O(5)-C(11)-C(12)-C(15)	-174.88(11)
N(1)-C(1)-C(2)-C(3)	6.57(11)
N(1)-C(9)-C(10)-O(2)	172.04(10)
N(1)-C(9)-C(10)-C(11)	-7.9(2)
N(1)-C(16)-C(17)-C(18)	-173.93(9)
C(1)-N(1)-C(9)-C(3)	-8.85(12)
C(1)-N(1)-C(9)-C(10)	173.60(11)
C(1)-N(1)-C(16)-C(17)	106.01(11)
C(1)-C(2)-C(3)-C(4)	-132.62(9)
C(1)-C(2)-C(3)-C(8)	104.33(10)
C(1)-C(2)-C(3)-C(9)	-10.94(11)
C(2)-C(3)-C(4)-C(5)	-68.03(12)
C(2)-C(3)-C(8)-C(7)	68.98(12)
C(2)-C(3)-C(9)-N(1)	12.09(11)
C(2)-C(3)-C(9)-C(10)	-170.27(10)
C(3)-C(4)-C(5)-C(6)	-56.59(12)
C(3)-C(9)-C(10)-O(2)	-5.16(16)
C(3)-C(9)-C(10)-C(11)	174.85(12)
C(4)-C(3)-C(8)-C(7)	-53.97(12)
C(4)-C(3)-C(9)-N(1)	131.47(10)
C(4)-C(3)-C(9)-C(10)	-50.89(14)
C(4)-C(5)-C(6)-C(7)	56.30(13)
C(5)-C(6)-C(7)-C(8)	-55.10(13)
C(6)-C(7)-C(8)-C(3)	54.22(13)
C(8)-C(3)-C(4)-C(5)	55.00(12)
C(8)-C(3)-C(9)-N(1)	-104.91(10)
C(8)-C(3)-C(9)-C(10)	72.73(13)
C(9)-N(1)-C(1)-O(1)	-178.81(10)
C(9)-N(1)-C(1)-C(2)	1.35(12)
C(9)-N(1)-C(16)-C(17)	-67.72(14)
C(9)-C(3)-C(4)-C(5)	177.30(9)
C(9)-C(3)-C(8)-C(7)	-179.47(9)
C(9)-C(10)-C(11)-O(5)	-4.8(2)
C(9)-C(10)-C(11)-C(12)	177.21(13)
C(10)-O(2)-C(15)-O(3)	-178.68(10)
C(10)-O(2)-C(15)-C(12)	0.81(12)
C(10)-C(11)-C(12)-C(13)	-176.13(10)
C(10)-C(11)-C(12)-C(15)	3.31(12)
C(11)-C(12)-C(13)-O(4)	-0.56(17)
C(11)-C(12)-C(13)-C(14)	179.91(11)
C(11)-C(12)-C(15)-O(2)	-2.60(12)
C(11)-C(12)-C(15)-O(3)	176.79(12)
C(13)-C(12)-C(15)-O(2)	176.78(11)
C(13)-C(12)-C(15)-O(3)	-3.8(2)
C(15)-O(2)-C(10)-C(9)	-178.74(9)
C(15)-O(2)-C(10)-C(11)	1.25(12)
C(15)-C(12)-C(13)-O(4)	-179.88(11)

C(15)-C(12)-C(13)-C(14)	0.59(19)
C(16)-N(1)-C(1)-O(1)	6.53(16)
C(16)-N(1)-C(1)-C(2)	-173.31(9)
C(16)-N(1)-C(9)-C(3)	165.23(10)
C(16)-N(1)-C(9)-C(10)	-12.32(18)
C(16)-C(17)-C(18)-C(19)	-79.00(13)
C(16)-C(17)-C(18)-C(23)	97.24(12)
C(17)-C(18)-C(19)-C(20)	176.03(11)
C(17)-C(18)-C(23)-C(22)	-175.95(10)
C(18)-C(19)-C(20)-C(21)	0.12(18)
C(19)-C(18)-C(23)-C(22)	0.39(17)
C(19)-C(20)-C(21)-C(22)	-0.02(18)
C(20)-C(21)-C(22)-C(23)	0.11(18)
C(21)-C(22)-C(23)-C(18)	-0.30(18)
C(23)-C(18)-C(19)-C(20)	-0.30(17)

Table S7. Hydrogen bonds for 4a [Å and °].

D-H...A	d(D-H)	d(H...A)	d(D...A)	<(DHA)
O(4)-H(4)...O(5)	1.051(16)	1.583(16)	2.5720(13)	154.5(14)

Crystallographic data for *(3E,5E)-3-(1-hydrazineylethylidene)-5-(3-oxo-2-phenethyl-2-azaspiro[4.5]decan-1-ylidene)furan-2,4(3H,5H)-dione* **5**

Table S8. Crystal data and structure refinement for 5.

Identification code	2352878	
Empirical formula	$C_{23}H_{27}N_3O_4$	
Formula weight	409.47	
Temperature	100.0(3) K	
Wavelength	1.54184 Å	
Crystal system	Monoclinic	
Space group	Cc	
Unit cell dimensions	a = 16.38100(10) Å	α= 90°.
	b = 16.3471(2) Å	β= 93.8830(10)°.
	c = 7.58130(10) Å	γ = 90°.
Volume	2025.47(4) $Å^3$	
Z	4	
Density (calculated)	1.343 g/cm^3	
Absorption coefficient	0.754 mm^{-1}	
F(000)	872	
Crystal size	0.4 x 0.31 x 0.2 mm^3	
Theta range for data collection	3.825 to 78.115°.	
Index ranges	-20<=h<=20, -20<=k<=20, -9<=l<=7	
Reflections collected	13483	
Independent reflections	2980 [R(int) = 0.0286]	
Observed reflections	2971	
Completeness to theta = 67.684°	99.9 %	
Absorption correction	Semi-empirical from equivalents	
Max. and min. transmission	1.00000 and 0.88376	
Refinement method	Full-matrix least-squares on F^2	
Data / restraints / parameters	2980 / 2 / 284	
Goodness-of-fit on F^2	1.067	
Final R indices [I>2sigma(I)]	R1 = 0.0306, wR2 = 0.0779	
R indices (all data)	R1 = 0.0307, wR2 = 0.0780	
Absolute structure parameter	-0.03(14)	
Largest diff. peak and hole	0.199 and -0.281 $e.Å^{-3}$	

Table S9. Atomic coordinates (x 10^4) and equivalent isotropic displacement parameters ($Å^2$x 10^3) for 5. U(eq) is defined as one third of the trace of the orthogonalized U^{ij} tensor.

	x	y	z	U(eq)
O(1)	6208(1)	1217(1)	6995(2)	20(1)
O(2)	5416(1)	4674(1)	7457(2)	16(1)
O(3)	3795(1)	3395(1)	5796(2)	18(1)
O(4)	4924(1)	5951(1)	7459(2)	18(1)
N(1)	5599(1)	2471(1)	6584(2)	14(1)
N(2)	2752(1)	4665(1)	5142(2)	17(1)
N(3)	1987(1)	4962(1)	4438(3)	22(1)
C(1)	6161(1)	1946(1)	7373(2)	15(1)
C(2)	6701(1)	2415(1)	8694(3)	17(1)
C(3)	6524(1)	3326(1)	8279(2)	13(1)
C(4)	6529(1)	3828(1)	9986(2)	16(1)
C(5)	7390(1)	3836(1)	10929(3)	21(1)
C(6)	7999(1)	4209(1)	9722(3)	25(1)
C(7)	8011(1)	3742(1)	7981(3)	22(1)
C(8)	7153(1)	3677(1)	7042(3)	17(1)
C(9)	5689(1)	3272(1)	7222(2)	13(1)
C(10)	5150(1)	3886(1)	6943(3)	14(1)
C(11)	4281(1)	3957(1)	6250(2)	14(1)
C(12)	4101(1)	4809(1)	6308(3)	15(1)
C(13)	3345(1)	5162(1)	5703(3)	15(1)
C(14)	3201(1)	6064(1)	5665(3)	22(1)
C(15)	4801(1)	5240(1)	7083(2)	14(1)
C(16)	5119(1)	2192(1)	4986(2)	15(1)
C(17)	5654(1)	2187(1)	3411(3)	18(1)
C(18)	5220(1)	1754(1)	1858(2)	15(1)
C(19)	4714(1)	2175(1)	612(3)	18(1)
C(20)	4294(1)	1761(1)	-773(3)	21(1)
C(21)	4375(1)	918(1)	-925(3)	22(1)
C(22)	4883(1)	491(1)	298(3)	21(1)
C(23)	5302(1)	908(1)	1677(3)	18(1)

Table S10. Bond lengths [Å] and angles [°] for 5.

O(1)-C(1)	1.229(2)
O(2)-C(10)	1.407(2)
O(2)-C(15)	1.383(2)
O(3)-C(11)	1.248(2)
O(4)-C(15)	1.210(2)
N(1)-C(1)	1.366(2)
N(1)-C(9)	1.401(2)
N(1)-C(16)	1.471(2)
N(2)-H(2)	0.89(3)
N(2)-N(3)	1.415(2)
N(2)-C(13)	1.314(2)
N(3)-H(3A)	0.89(3)
N(3)-H(3B)	0.86(3)
C(1)-C(2)	1.501(2)
C(2)-H(2A)	0.9900
C(2)-H(2B)	0.9900
C(2)-C(3)	1.546(2)
C(3)-C(4)	1.532(3)
C(3)-C(8)	1.550(3)
C(3)-C(9)	1.541(2)
C(4)-H(4A)	0.9900
C(4)-H(4B)	0.9900
C(4)-C(5)	1.537(2)
C(5)-H(5A)	0.9900
C(5)-H(5B)	0.9900
C(5)-C(6)	1.526(3)
C(6)-H(6A)	0.9900
C(6)-H(6B)	0.9900
C(6)-C(7)	1.526(3)
C(7)-H(7A)	0.9900
C(7)-H(7B)	0.9900
C(7)-C(8)	1.535(2)
C(8)-H(8A)	0.9900
C(8)-H(8B)	0.9900
C(9)-C(10)	1.343(3)
C(10)-C(11)	1.487(2)
C(11)-C(12)	1.426(2)
C(12)-C(13)	1.414(2)
C(12)-C(15)	1.437(2)
C(13)-C(14)	1.493(2)
C(14)-H(14A)	0.9800
C(14)-H(14B)	0.9800
C(14)-H(14C)	0.9800
C(16)-H(16A)	0.9900
C(16)-H(16B)	0.9900
C(16)-C(17)	1.528(3)
C(17)-H(17A)	0.9900
C(17)-H(17B)	0.9900
C(17)-C(18)	1.509(2)
C(18)-C(19)	1.396(3)
C(18)-C(23)	1.397(2)

C(19)-H(19)	0.9500
C(19)-C(20)	1.392(3)
C(20)-H(20)	0.9500
C(20)-C(21)	1.391(3)
C(21)-H(21)	0.9500
C(21)-C(22)	1.392(3)
C(22)-H(22)	0.9500
C(22)-C(23)	1.389(3)
C(23)-H(23)	0.9500

C(15)-O(2)-C(10)	110.41(13)
C(1)-N(1)-C(9)	112.55(14)
C(1)-N(1)-C(16)	118.24(15)
C(9)-N(1)-C(16)	127.73(15)
N(3)-N(2)-H(2)	121.1(17)
C(13)-N(2)-H(2)	117.1(17)
C(13)-N(2)-N(3)	121.80(16)
N(2)-N(3)-H(3A)	105.3(17)
N(2)-N(3)-H(3B)	104.5(16)
H(3A)-N(3)-H(3B)	111(2)
O(1)-C(1)-N(1)	123.85(17)
O(1)-C(1)-C(2)	127.37(17)
N(1)-C(1)-C(2)	108.76(15)
C(1)-C(2)-H(2A)	110.7
C(1)-C(2)-H(2B)	110.7
C(1)-C(2)-C(3)	105.18(14)
H(2A)-C(2)-H(2B)	108.8
C(3)-C(2)-H(2A)	110.7
C(3)-C(2)-H(2B)	110.7
C(2)-C(3)-C(8)	110.89(15)
C(4)-C(3)-C(2)	110.74(15)
C(4)-C(3)-C(8)	110.34(15)
C(4)-C(3)-C(9)	115.03(15)
C(9)-C(3)-C(2)	101.47(13)
C(9)-C(3)-C(8)	108.06(14)
C(3)-C(4)-H(4A)	109.6
C(3)-C(4)-H(4B)	109.6
C(3)-C(4)-C(5)	110.45(15)
H(4A)-C(4)-H(4B)	108.1
C(5)-C(4)-H(4A)	109.6
C(5)-C(4)-H(4B)	109.6
C(4)-C(5)-H(5A)	109.7
C(4)-C(5)-H(5B)	109.7
H(5A)-C(5)-H(5B)	108.2
C(6)-C(5)-C(4)	109.95(16)
C(6)-C(5)-H(5A)	109.7
C(6)-C(5)-H(5B)	109.7
C(5)-C(6)-H(6A)	109.3
C(5)-C(6)-H(6B)	109.3
H(6A)-C(6)-H(6B)	108.0
C(7)-C(6)-C(5)	111.50(16)
C(7)-C(6)-H(6A)	109.3
C(7)-C(6)-H(6B)	109.3

C(6)-C(7)-H(7A)	109.3
C(6)-C(7)-H(7B)	109.3
C(6)-C(7)-C(8)	111.80(17)
H(7A)-C(7)-H(7B)	107.9
C(8)-C(7)-H(7A)	109.3
C(8)-C(7)-H(7B)	109.3
C(3)-C(8)-H(8A)	109.3
C(3)-C(8)-H(8B)	109.3
C(7)-C(8)-C(3)	111.73(15)
C(7)-C(8)-H(8A)	109.3
C(7)-C(8)-H(8B)	109.3
H(8A)-C(8)-H(8B)	107.9
N(1)-C(9)-C(3)	107.57(14)
C(10)-C(9)-N(1)	126.23(15)
C(10)-C(9)-C(3)	126.20(16)
O(2)-C(10)-C(11)	107.32(15)
C(9)-C(10)-O(2)	116.86(15)
C(9)-C(10)-C(11)	135.77(17)
O(3)-C(11)-C(10)	128.13(17)
O(3)-C(11)-C(12)	126.70(16)
C(12)-C(11)-C(10)	105.09(15)
C(11)-C(12)-C(15)	109.31(16)
C(13)-C(12)-C(11)	124.57(16)
C(13)-C(12)-C(15)	126.12(16)
N(2)-C(13)-C(12)	117.67(17)
N(2)-C(13)-C(14)	119.41(16)
C(12)-C(13)-C(14)	122.91(16)
C(13)-C(14)-H(14A)	109.5
C(13)-C(14)-H(14B)	109.5
C(13)-C(14)-H(14C)	109.5
H(14A)-C(14)-H(14B)	109.5
H(14A)-C(14)-H(14C)	109.5
H(14B)-C(14)-H(14C)	109.5
O(2)-C(15)-C(12)	107.77(15)
O(4)-C(15)-O(2)	119.18(16)
O(4)-C(15)-C(12)	133.04(17)
N(1)-C(16)-H(16A)	109.7
N(1)-C(16)-H(16B)	109.7
N(1)-C(16)-C(17)	110.04(14)
H(16A)-C(16)-H(16B)	108.2
C(17)-C(16)-H(16A)	109.7
C(17)-C(16)-H(16B)	109.7
C(16)-C(17)-H(17A)	109.6
C(16)-C(17)-H(17B)	109.6
H(17A)-C(17)-H(17B)	108.1
C(18)-C(17)-C(16)	110.36(15)
C(18)-C(17)-H(17A)	109.6
C(18)-C(17)-H(17B)	109.6
C(19)-C(18)-C(17)	121.55(16)
C(19)-C(18)-C(23)	118.55(17)
C(23)-C(18)-C(17)	119.86(17)
C(18)-C(19)-H(19)	119.6
C(20)-C(19)-C(18)	120.78(17)

C(20)-C(19)-H(19)	119.6
C(19)-C(20)-H(20)	120.0
C(21)-C(20)-C(19)	119.96(18)
C(21)-C(20)-H(20)	120.0
C(20)-C(21)-H(21)	120.1
C(20)-C(21)-C(22)	119.89(17)
C(22)-C(21)-H(21)	120.1
C(21)-C(22)-H(22)	120.1
C(23)-C(22)-C(21)	119.85(18)
C(23)-C(22)-H(22)	120.1
C(18)-C(23)-H(23)	119.5
C(22)-C(23)-C(18)	120.96(18)
C(22)-C(23)-H(23)	119.5

Symmetry transformations used to generate equivalent atoms:

Table S11. Anisotropic displacement parameters ($Å^2$ x 10^3) for 5. The anisotropic displacement factor exponent takes the form: $-2p^2[h^2 a^{*2}U^{11} + ... + 2 h k a^* b^* U^{12}]$

	U^{11}	U^{22}	U^{33}	U^{23}	U^{13}	U^{12}
O(1)	21(1)	14(1)	23(1)	1(1)	0(1)	2(1)
O(2)	13(1)	13(1)	21(1)	-1(1)	-2(1)	0(1)
O(3)	14(1)	16(1)	24(1)	-2(1)	-1(1)	-1(1)
O(4)	16(1)	15(1)	24(1)	-1(1)	1(1)	-1(1)
N(1)	14(1)	15(1)	14(1)	-2(1)	-1(1)	0(1)
N(2)	13(1)	18(1)	20(1)	-2(1)	-2(1)	3(1)
N(3)	13(1)	23(1)	28(1)	-8(1)	-6(1)	3(1)
C(1)	13(1)	18(1)	15(1)	2(1)	3(1)	1(1)
C(2)	15(1)	18(1)	17(1)	2(1)	-1(1)	2(1)
C(3)	11(1)	16(1)	13(1)	0(1)	0(1)	1(1)
C(4)	15(1)	20(1)	14(1)	-2(1)	-1(1)	1(1)
C(5)	19(1)	27(1)	15(1)	-3(1)	-4(1)	0(1)
C(6)	16(1)	31(1)	26(1)	-2(1)	-5(1)	-7(1)
C(7)	12(1)	33(1)	23(1)	1(1)	1(1)	-3(1)
C(8)	14(1)	24(1)	14(1)	2(1)	0(1)	-1(1)
C(9)	12(1)	16(1)	12(1)	1(1)	1(1)	-2(1)
C(10)	14(1)	14(1)	15(1)	0(1)	0(1)	-3(1)
C(11)	12(1)	18(1)	13(1)	0(1)	1(1)	1(1)
C(12)	14(1)	16(1)	16(1)	0(1)	1(1)	0(1)
C(13)	13(1)	19(1)	14(1)	-1(1)	3(1)	1(1)
C(14)	17(1)	16(1)	32(1)	-1(1)	-3(1)	2(1)
C(15)	13(1)	16(1)	15(1)	1(1)	3(1)	1(1)
C(16)	14(1)	15(1)	16(1)	-2(1)	-2(1)	0(1)
C(17)	18(1)	19(1)	16(1)	0(1)	-1(1)	-3(1)
C(18)	14(1)	19(1)	13(1)	0(1)	3(1)	-1(1)
C(19)	17(1)	18(1)	19(1)	2(1)	2(1)	0(1)
C(20)	19(1)	28(1)	16(1)	2(1)	-1(1)	-1(1)
C(21)	21(1)	29(1)	15(1)	-4(1)	3(1)	-9(1)
C(22)	23(1)	18(1)	21(1)	-4(1)	8(1)	-2(1)
C(23)	17(1)	18(1)	17(1)	2(1)	3(1)	1(1)

Table S12. Hydrogen coordinates (x 10^4) and isotropic displacement parameters (Å^{2}x 10^3) for 5.

	x	y	z	U(eq)
H(2)	2855(16)	4130(18)	5210(40)	21(6)
H(3A)	1715(16)	5101(18)	5380(40)	23(7)
H(3B)	1761(15)	4547(16)	3900(30)	12(5)
H(2A)	7285	2286	8563	20
H(2B)	6568	2281	9915	20
H(4A)	6136	3589	10778	20
H(4B)	6354	4396	9704	20
H(5A)	7384	4160	12032	25
H(5B)	7558	3271	11248	25
H(6A)	7850	4787	9476	30
H(6B)	8553	4200	10330	30
H(7A)	8231	3186	8215	27
H(7B)	8379	4026	7196	27
H(8A)	7176	3319	5993	21
H(8B)	6972	4226	6626	21
H(14A)	3236	6267	4456	33
H(14B)	3616	6336	6451	33
H(14C)	2656	6181	6062	33
H(16A)	4646	2561	4736	18
H(16B)	4907	1635	5180	18
H(17A)	5779	2756	3075	21
H(17B)	6177	1905	3745	21
H(19)	4656	2751	711	21
H(20)	3952	2055	-1615	25
H(21)	4083	633	-1862	26
H(22)	4945	-85	190	25
H(23)	5648	613	2509	21

Table S13. Torsion angles [°] for 5.

O(1)-C(1)-C(2)-C(3)	165.20(19)
O(2)-C(10)-C(11)-O(3)	-173.48(19)
O(2)-C(10)-C(11)-C(12)	3.3(2)
O(3)-C(11)-C(12)-C(13)	-5.7(3)
O(3)-C(11)-C(12)-C(15)	173.79(19)
N(1)-C(1)-C(2)-C(3)	-13.2(2)
N(1)-C(9)-C(10)-O(2)	-169.88(17)
N(1)-C(9)-C(10)-C(11)	12.9(4)
N(1)-C(16)-C(17)-C(18)	169.26(15)
N(3)-N(2)-C(13)-C(12)	-177.11(17)
N(3)-N(2)-C(13)-C(14)	2.4(3)
C(1)-N(1)-C(9)-C(3)	13.4(2)
C(1)-N(1)-C(9)-C(10)	-167.42(19)
C(1)-N(1)-C(16)-C(17)	-73.9(2)
C(1)-C(2)-C(3)-C(4)	142.21(15)
C(1)-C(2)-C(3)-C(8)	-94.94(17)
C(1)-C(2)-C(3)-C(9)	19.64(18)
C(2)-C(3)-C(4)-C(5)	65.47(19)
C(2)-C(3)-C(8)-C(7)	-68.9(2)
C(2)-C(3)-C(9)-N(1)	-20.21(18)
C(2)-C(3)-C(9)-C(10)	160.64(19)
C(3)-C(4)-C(5)-C(6)	59.6(2)
C(3)-C(9)-C(10)-O(2)	9.1(3)
C(3)-C(9)-C(10)-C(11)	-168.1(2)
C(4)-C(3)-C(8)-C(7)	54.2(2)
C(4)-C(3)-C(9)-N(1)	-139.78(16)
C(4)-C(3)-C(9)-C(10)	41.1(3)
C(4)-C(5)-C(6)-C(7)	-57.7(2)
C(5)-C(6)-C(7)-C(8)	54.5(2)
C(6)-C(7)-C(8)-C(3)	-52.6(2)
C(8)-C(3)-C(4)-C(5)	-57.69(19)
C(8)-C(3)-C(9)-N(1)	96.46(17)
C(8)-C(3)-C(9)-C(10)	-82.7(2)
C(9)-N(1)-C(1)-O(1)	-178.50(18)
C(9)-N(1)-C(1)-C(2)	0.0(2)
C(9)-N(1)-C(16)-C(17)	91.0(2)
C(9)-C(3)-C(4)-C(5)	179.76(15)
C(9)-C(3)-C(8)-C(7)	-179.28(16)
C(9)-C(10)-C(11)-O(3)	3.9(4)
C(9)-C(10)-C(11)-C(12)	-179.4(2)
C(10)-O(2)-C(15)-O(4)	179.21(18)
C(10)-O(2)-C(15)-C(12)	0.5(2)
C(10)-C(11)-C(12)-C(13)	177.46(18)
C(10)-C(11)-C(12)-C(15)	-3.0(2)
C(11)-C(12)-C(13)-N(2)	4.1(3)
C(11)-C(12)-C(13)-C(14)	-175.4(2)
C(11)-C(12)-C(15)-O(2)	1.7(2)
C(11)-C(12)-C(15)-O(4)	-176.8(2)
C(13)-C(12)-C(15)-O(2)	-178.79(17)
C(13)-C(12)-C(15)-O(4)	2.7(4)
C(15)-O(2)-C(10)-C(9)	179.70(17)

C(15)-O(2)-C(10)-C(11)	-2.4(2)
C(15)-C(12)-C(13)-N(2)	-175.38(19)
C(15)-C(12)-C(13)-C(14)	5.2(3)
C(16)-N(1)-C(1)-O(1)	-11.3(3)
C(16)-N(1)-C(1)-C(2)	167.19(16)
C(16)-N(1)-C(9)-C(3)	-152.26(16)
C(16)-N(1)-C(9)-C(10)	26.9(3)
C(16)-C(17)-C(18)-C(19)	89.9(2)
C(16)-C(17)-C(18)-C(23)	-88.1(2)
C(17)-C(18)-C(19)-C(20)	-177.48(17)
C(17)-C(18)-C(23)-C(22)	177.46(18)
C(18)-C(19)-C(20)-C(21)	0.1(3)
C(19)-C(18)-C(23)-C(22)	-0.6(3)
C(19)-C(20)-C(21)-C(22)	-0.8(3)
C(20)-C(21)-C(22)-C(23)	0.7(3)
C(21)-C(22)-C(23)-C(18)	0.0(3)
C(23)-C(18)-C(19)-C(20)	0.5(3)

Table S14. Hydrogen bonds for 5 [Å and °].

D-H...A	d(D-H)	d(H...A)	d(D...A)	<(DHA)
N(2)-H(2)...O(3)	0.89(3)	1.98(3)	2.713(2)	138(2)
N(3)-H(3A)...O(1)#1	0.89(3)	2.38(3)	3.152(2)	145(2)
N(3)-H(3B)...O(1)#2	0.86(3)	2.07(3)	2.908(2)	163(2)

Symmetry transformations used to generate equivalent atoms:
#1 x-1/2,y+1/2,z   #2 x-1/2,-y+1/2,z-1/2
