# Supplementary material for: Base-promoted cascade recyclization of allomaltol derivatives containing an amide fragment into substituted 3-(1-hydroxyethylidene)tetronic acids
Source: Beilstein J Org Chem. 2024 Oct 14;20:2585–91. doi: 10.3762/bjoc.20.217 (PMC11496715; doi:10.3762/bjoc.20.217)
Supplement: File 2 — Analytical data of all compounds 4, 7 and 9. [file Beilstein_J_Org_Chem-20-2585-s002.pdf]

BEILSTEIN JOURNAL OF ORGANIC CHEMISTRY

Supporting Information

for

Base-promoted cascade recyclization of allomaltol derivatives containing an amide fragment into substituted 3-(1-hydroxyethylidene)tetronic acids

Andrey N. Komogortsev, Constantine V. Milyutin and Boris V. Lichitsky

Beilstein J. Org. Chem. **2024,** *20,* 2585–2591. doi:10.3762/bjoc.20.217

Analytical data of all compounds 4, 7 and 9

License and Terms: This is a supporting information file under the terms of the Creative Commons Attribution License (https://creativecommons.org/licenses/by/4.0). Please note that the reuse, redistribution and reproduction in particular requires that the author(s) and source are credited and that individual graphics may be subject to special legal provisions.
The license is subject to the *Beilstein Journal of Organic Chemistry* terms and conditions: (https://www.beilstein-journals.org/bjoc/terms)

Table of content

page

Analytical data for compounds **4a–t** S2

Analytical data for compound **7** S8

Analytical data for compound **9** S9

Analytical data for compounds 4a–t

(3*E*,5*E*)-3-(1-hydroxyethylidene)-5-(3-oxo-2-phenethyl-2-azaspiro[4.5]decan-1-ylidene)furan-2,4(3*H*,5*H*)-dione (**4a**). Pale yellow powder; yield 62% (0.25 g); mp 121-123°C. ^{1}H NMR (300 MHz, DMSO-d_6) δ 7.31 – 7.13 (m, 5H), 4.34 (t, *J* = 7.5 Hz, 2H), 2.74 (t, *J* = 7.6 Hz, 2H), 2.56-2.48 (m, 2H in DMSO), 2.46 (s, 3H), 2.14 (t, *J* = 11.8 Hz, 2H), 1.64 – 1.54 (m, 3H), 1.41 – 1.09 (m, 5H). ^{13}C NMR (75 MHz, DMSO-d_6) δ 178.2, 175.5, 164.8, 138.1, 128.9, 128.3, 126.4, 98.7, 44.3, 44.0, 39.4, 32.6, 32.1, 24.7, 23.9, 21.9. HRMS (ESI-TOF) *m/z*: Calcld for $C_{23}H_{26}NO_5$ $[M+H]^+$ 396.1805; Found: 396.1801.

(3*E*,5*E*)-3-(1-hydroxyethylidene)-5-(3-oxo-2-phenethyl-8-oxa-2-azaspiro[4.5]decan-1-ylidene)furan-2,4(3*H*,5*H*)-dione (**4b**). White powder; yield 59% (0.23 g); mp 161-163°C. ^{1}H NMR (300 MHz, DMSO-d_6) δ 7.32 – 7.13 (m, 5H), 4.37 (t, *J* = 7.3 Hz, 2H), 3.84 – 3.73 (m, 2H), 3.39 (t, *J* = 11.9 Hz, 2H), 2.74 (t, *J* = 7.5 Hz, 2H), 2.66 (s, 2H), 2.55-2.48 (m, 2H in DMSO), 2.46 (s, 3H), 1.27 – 1.17 (m, 2H). ^{13}C NMR (75 MHz, DMSO-d_6) δ 192.0, 178.3, 175.0, 164.9, 143.5, 138.1, 128.9, 128.2, 126.3, 98.6, 63.5, 43.9, 41.7, 38.8, 32.6, 32.3, 24.0. HRMS (ESI-TOF) *m/z*: Calcld for $C_{22}H_{24}NO_6$ $[M+H]^+$ 398.1598; Found: 398.1610.

(3*E*,5*E*)-3-(1-hydroxyethylidene)-5-(3-oxo-2-phenethyl-8-thia-2-azaspiro[4.5]decan-1-ylidene)furan-2,4(3*H*,5*H*)-dione (**4c**). Pale yellow powder; yield 71% (0.29 g); mp 170-172°C. ^{1}H NMR (300 MHz, DMSO-d_6) δ 7.31 – 7.13 (m, 5H), 4.34 (t, *J* = 7.3 Hz, 2H), 2.81 – 2.64 (m, 5H), 2.58 – 2.55 (m, 2H), 2.47 – 2.40 (m, 6H), 1.66 – 1.56 (m, 2H). ^{13}C NMR (75 MHz, DMSO-d_6) δ 192.0, 178.4, 175.0, 164.7, 138.1, 128.9, 128.3, 126.3, 126.2, 98.7, 43.9, 43.5, 38.1, 32.5, 23.8, 23.6. HRMS (ESI-TOF) *m/z*: Calcld for $C_{22}H_{24}NO_5S$ $[M+H]^+$ 414.1370; Found: 414.1361.

(3*E*,5*E*)-5-(2-(4-fluorophenethyl)-3-oxo-2-azaspiro[4.5]decan-1-ylidene)-3-(1-hydroxyethylidene)furan-2,4(3*H*,5*H*)-dione (**4d**). White powder; yield 56% (0.23 g); mp

134-136°C. ^{1}H NMR (300 MHz, DMSO-d_6) δ 8.97 (br. s, 1H), 7.24 – 7.13 (m, 2H), 7.11 – 6.99 (m, 2H), 4.29 (t, *J* = 7.6 Hz, 2H), 2.71 (t, *J* = 7.6 Hz, 2H), 2.55-2.48 (m, 2H in DMSO), 2.44 (s, 3H), 2.13 (d, *J* = 11.6 Hz, 2H), 1.68 – 1.50 (m, 3H), 1.43 – 1.03 (m, 5H). ^{13}C NMR (75 MHz, DMSO-d_6) δ 192.2, 178.2, 175.4, 161.0 (d, J_{CF} = 242.0 Hz), 159.4, 145.9, 134.3, 134.3, 130.7 (d, J_{CF} = 8.0 Hz), 125.9, 114.9 (d, J_{CF} = 21.0 Hz), 98.7, 44.20, 44.0, 41.7, 32.1, 31.8, 24.7, 23.8, 21.9. HRMS (ESI-TOF) *m/z*: Calcld for $C_{23}H_{25}FNO_5$ $[M+H]^+$ 414.1711; Found: 414.1711.

(3*E*,5*E*)-5-(2-(4-chlorophenethyl)-3-oxo-8-oxa-2-azaspiro[4.5]decan-1-ylidene)-3-(1-hydroxyethylidene)furan-2,4(3*H*,5*H*)-dione (**4e**). White powder; yield 67% (0.29 g); mp 96-98°C. ^{1}H NMR (300 MHz, DMSO-d_6) δ 9.52 (br. s, 1H), 7.31 (d, *J* = 8.0 Hz, 2H), 7.21 (d, *J* = 8.0 Hz, 2H), 4.35 (t, *J* = 7.2 Hz, 2H), 3.85 – 3.74 (m, 2H), 3.38 (t, *J* = 12.1 Hz, 2H), 2.74 (t, *J* = 7.4 Hz, 2H), 2.67 (s, 2H), 2.45 (s, 3H), 2.07 (s, 2H), 1.25 (d, *J* = 12.9 Hz, 2H). ^{13}C NMR (75 MHz, DMSO-d_6) δ 192.0, 178.4, 175.1, 164.9, 143.7, 137.3, 131.1, 130.9, 128.2, 126.3, 98.7, 63.6, 43.9, 41.8, 38.8, 32.4, 32.1, 23.9. HRMS (ESI-TOF) *m/z*: Calcld for $C_{22}H_{23}ClNO_6$ $[M+H]^+$ 432.1208; Found: 432.1205.

(3*E*,5*E*)-5-(2-(3-chlorophenethyl)-3-oxo-2-azaspiro[4.5]decan-1-ylidene)-3-(1-hydroxyethylidene)furan-2,4(3*H*,5*H*)-dione (**4f**). Pale yellow powder; yield 63% (0.27 g); mp 133-135°C. ^{1}H NMR (300 MHz, DMSO-d_6) δ 7.35 – 7.19 (m, 3H), 7.17 – 7.09 (m, 1H), 4.34 (t, *J* = 7.3 Hz, 2H), 2.75 (t, *J* = 7.2 Hz, 2H), 2.56-2.48 (m, 2H in DMSO), 2.46 (s, 3H), 2.13 (t, *J* = 12.1 Hz, 2H), 1.64 – 1.54 (m, 3H), 1.42 – 1.06 (m, 5H). ^{13}C NMR (75 MHz, DMSO-d_6) δ 192.1, 178.2, 175.3, 164.6, 145.7, 140.7, 132.8, 130.0, 128.7, 127.7, 126.3, 126.0, 98.7, 44.2, 43.7, 32.2, 32.1, 24.6, 23.7, 21.9. HRMS (ESI-TOF) *m/z*: Calcld for $C_{23}H_{25}ClNO_5$ $[M+H]^+$ 430.1416; Found: 430.1434.

(3*E*,5*E*)-5-(2-(3-chlorophenethyl)-3-oxo-8-oxa-2-azaspiro[4.5]decan-1-ylidene)-3-(1-hydroxyethylidene)furan-2,4(3*H*,5*H*)-dione (**4g**). White powder; yield 61% (0.26 g); mp 220-222°C. ^{1}H NMR (300 MHz, DMSO-d_6) δ 11.81 (br. s, 1H), 7.31 – 7.20 (m, 3H), 7.18 – 7.11 (m, 1H), 4.38 (t, *J* = 7.3 Hz, 2H), 3.85 – 3.73 (m, 2H), 3.38 (t, *J* = 12.1 Hz, 2H), 2.76 (t, *J* = 7.3 Hz, 2H), 2.66 (s, 2H), 2.56-2.48 (m, 2H in DMSO), 2.46 (s, 3H), 1.24 (d, *J* = 13.0 Hz, 2H). ^{13}C NMR (75 MHz, DMSO-d_6) δ 191.8, 178.4, 175.0, 164.8, 143.5, 140.8, 132.8, 130.0, 128.9, 127.7, 126.3, 98.6, 63.5, 43.7, 41.7, 38.8, 32.3, 23.8. HRMS (ESI-TOF) *m/z*: Calcld for $C_{22}H_{23}ClNO_6$ $[M+H]^+$ 432.1208; Found: 432.1227.

(3*E*,5*E*)-5-(2-benzyl-3-oxo-2-azaspiro[4.5]decan-1-ylidene)-3-(1-hydroxyethylidene)furan-2,4(3*H*,5*H*)-dione (**4h**). White powder; yield 72% (0.27 g); mp 145-147°C. ^{1}H NMR (300 MHz, DMSO-d_6) δ 7.32 – 7.14 (m, 3H), 7.08 (d, *J* = 7.3 Hz, 2H), 5.41 (s, 2H), 2.68 (s, 2H), 2.39 (s, 3H), 2.17 – 2.02 (m, 2H), 1.66 – 1.56 (m, 3H), 1.47 – 1.33 (m, 3H), 1.33 – 1.25 (m, 1H), 1.24 – 1.09 (m, 1H). ^{13}C NMR (75 MHz, DMSO-d_6) δ 191.9, 178.21, 175.79, 164.59, 145.23, 136.61, 128.35, 127.19, 126.92, 126.36, 98.48, 45.56, 44.31, 31.86, 24.67, 23.62, 21.82. HRMS (ESI-TOF) *m/z*: $[M+H]^+$ Calcld for $C_{22}H_{24}NO_5$: 382.1649; Found: 382.1639.

(3*E*,5*E*)-5-(2-benzyl-3-oxo-8-thia-2-azaspiro[4.5]decan-1-ylidene)-3-(1-hydroxyethylidene)furan-2,4(3*H*,5*H*)-dione (**4i**). Pale yellow powder; yield 75% (0.30 g); mp 149-151°C. ^{1}H NMR (300 MHz, DMSO-d_6) δ 7.32 – 7.17 (m, 3H), 7.13 – 7.04 (m, 2H), 5.41 (s, 2H), 2.83 – 2.70 (m, 4H), 2.56 – 2.30 (m, 7H), 1.74 – 1.64 (m, 2H). ^{13}C NMR (75 MHz, DMSO-d_6) δ 191.6, 178.5, 175.3, 164.8, 143.3, 136.4, 128.4, 127.2, 127.0, 126.8, 126.6, 98.4, 45.6, 43.5, 37.9, 32.4, 23.7, 23.6. HRMS (ESI-TOF) *m/z*: Calcld for $C_{21}H_{22}NO_5S$ $[M+H]^+$ 400.1213; Found: 400.1230.

(3*E*,5*E*)-3-(1-hydroxyethylidene)-5-(2-(2-methoxybenzyl)-3-oxo-2-azaspiro[4.5]decan-1-ylidene)furan-2,4(3*H*,5*H*)-dione (**4j**). Yellow powder; yield 70% (0.29 g); mp 164-166°C. ^{1}H NMR (300 MHz, DMSO-d_6) δ 7.25 – 7.14 (m, 1H), 6.97 – 6.87 (m, 2H), 6.86 – 6.75 (m, 1H), 5.26 (s, 2H), 3.72 (s, 3H), 2.65 (s, 2H), 2.39 (s, 3H), 2.21 – 2.03 (m, 2H), 1.63 (d, *J* = 12.1 Hz, 3H), 1.46 (d, *J* = 13.0 Hz, 2H), 1.39 – 1.25 (m, 2H), 1.25 – 1.12 (m, 1H). ^{13}C NMR (75 MHz, DMSO-d_6) δ 192.6, 177.5, 175.6, 164.5, 156.6, 146.8, 128.4, 127.4, 125.8, 124.1, 119.9, 110.6, 98.4, 55.1, 44.4, 43.0, 39.1, 32.1, 24.7, 23.8, 21.9. HRMS (ESI-TOF) *m/z*: Calcld for $C_{23}H_{26}NO_6$ $[M+H]^+$ 412.1755; Found: 412.1776.

(3*E*,5*E*)-3-(1-hydroxyethylidene)-5-(2-(3-methoxybenzyl)-3-oxo-2-azaspiro[4.5]decan-1-ylidene)furan-2,4(3*H*,5*H*)-dione (**4k**). Yellow powder; yield 67% (0.28 g); mp 87-89°C. ^{1}H NMR (300 MHz, DMSO-d_6) δ 7.17 (t, *J* = 7.8 Hz, 1H), 6.80 – 6.72 (m, 1H), 6.68 – 6.59 (m, 2H), 5.39 (s, 2H), 3.66 (s, 3H), 2.68 (s, 2H), 2.38 (s, 3H), 2.18 – 2.03 (m, 2H), 1.67 – 1.56 (m, 3H), 1.48 – 1.24 (m, 4H), 1.21 – 1.10 (m, 1H). ^{13}C NMR (75 MHz, DMSO-d_6) δ 191.7, 178.3, 175.7, 165.0, 159.1, 138.3, 129.5, 126.7, 119.0, 112.7, 112.5, 98.3, 54.9, 45.4, 44.2, 31.9, 24.7, 24.1, 21.8. HRMS (ESI-TOF) *m/z*: Calcld for $C_{23}H_{26}NO_6$ $[M+H]^+$ 412.1755; Found: 412.1755.

(3*E*,5*E*)-3-(1-hydroxyethylidene)-5-(2-(4-methylbenzyl)-3-oxo-2-azaspiro[4.5]decan-1-ylidene)furan-2,4(3*H*,5*H*)-dione (**4l**). White powder; yield 68% (0.27 g); mp 97-99°C. ^{1}H NMR (300 MHz, DMSO-d_6) δ 7.04 (d, *J* = 7.6 Hz, 2H), 6.96 (d, *J* = 7.8 Hz, 2H), 5.37 (s, 2H), 2.63 (s, 2H), 2.35 (s, 3H), 2.21 (s, 3H), 2.06 (t, *J* = 12.3 Hz, 2H), 1.66 – 1.53 (m, 4H), 1.41 – 1.26 (m, 4H). ^{13}C NMR (75 MHz, DMSO-d_6) δ 191.6, 178.6, 175.8, 136.3, 133.8, 128.9, 127.2, 126.7, 119.3, 98.1, 45.0, 44.0, 31.9, 25.0, 24.7, 22.3, 21.8, 20.6. HRMS (ESI-TOF) *m/z*: Calcld for $C_{23}H_{26}NO_5$ $[M+H]^+$ 396.1805; Found: 396.1824.

(3*E*,5*E*)-3-(1-hydroxyethylidene)-5-(2-(3-methoxypropyl)-3-oxo-2-azaspiro[4.5]decan-1-ylidene)furan-2,4(3*H*,5*H*)-dione (**4m**). Pale yellow powder; yield 54% (0.19 g); mp 106-108°C. ^{1}H NMR (300 MHz, DMSO-d_6) δ 9.06 (br. s, 1H), 4.13 (t, *J* = 6.9 Hz, 2H), 3.27 (t, *J* = 6.1 Hz, 2H), 3.16 (s, 3H), 2.60 (s, 2H), 2.44 (s, 3H), 2.27 – 2.12 (m, 2H), 1.71 – 1.57 (m, 5H), 1.46 (d, *J* = 13.0 Hz, 2H), 1.41 – 1.14 (m, 3H). ^{13}C NMR (75 MHz, DMSO-d_6) δ 192.8, 177.6, 175.5, 164.4, 153.9, 147.0, 125.1, 98.7, 69.4, 57.8, 44.4, 40.7, 32.0, 26.8, 24.6, 23.9, 21.9. HRMS (ESI-TOF) *m/z*: Calcld for $C_{19}H_{26}NO_6$ $[M+H]^+$ 364.1755; Found: 364.1775.

(3*E*,5*E*)-3-(1-hydroxyethylidene)-5-(2-(3-methoxypropyl)-3-oxo-8-thia-2-azaspiro[4.5]decan-1-ylidene)furan-2,4(3*H*,5*H*)-dione (**4n**). Yellow powder; yield 58% (0.22 g); mp 151-153°C. ^{1}H NMR (300 MHz, DMSO-d_6) δ 4.15 (t, *J* = 6.7 Hz, 2H), 3.26 (t, *J* = 6.1 Hz, 2H), 3.15 (s, 3H), 2.76 (t, *J* = 13.9 Hz, 2H), 2.63 (s, 2H), 2.42 (s, 3H), 1.74 (d, *J* = 12.3 Hz, 2H), 1.70 – 1.56 (m, 2H). ^{13}C NMR (75 MHz, DMSO-d_6) δ 192.5, 177.9, 175.0, 164.6, 145.1, 125.6, 98.7, 69.5, 57.8, 43.6, 40.7, 38.0, 32.5, 31.7, 26.8, 23.6. HRMS (ESI-TOF) *m/z*: Calcld for $C_{18}H_{24}NO_6S$ $[M+H]^+$ 382.1319; Found: 382.1333.

(3*E*,5*E*)-3-(1-hydroxyethylidene)-5-(3-oxo-2-phenethyl-2-azaspiro[4.4]nonan-1-ylidene)furan-2,4(3*H*,5*H*)-dione (**4o**). Pale yellow powder; yield 62% (0.24 g); mp 103-105°C. ^{1}H NMR (300 MHz, DMSO-d_6) δ 9.45 (br. s, 1H), 7.30 – 7.21 (m, 2H), 7.21 – 7.13 (m, 3H), 4.37 (t, *J* = 7.3 Hz, 2H), 2.77 (t, *J* = 7.4 Hz, 2H), 2.47 (s, 2H), 2.46 (s, 3H), 2.28 – 2.13 (m, 2H), 1.88 – 1.76 (m, 2H), 1.68 – 1.58 (m, 2H), 1.56 – 1.45 (m, 2H). ^{13}C NMR (75 MHz, DMSO-d_6) δ 179.7, 175.1, 166.8, 164.6, 140.2, 138.3, 128.9, 128.2, 127.8, 126.2, 88.9, 43.6, 43.4, 39.6, 32.7, 32.2, 24.8, 21.9, 12.7. HRMS (ESI-TOF) *m/z*: Calcld for $C_{22}H_{24}NO_5$ $[M+H]^+$ 382.1649; Found: 382.1672.

(3*E*,5*E*)-5-(2-(3-chlorophenethyl)-3-oxo-2-azaspiro[4.4]nonan-1-ylidene)-3-(1-hydroxyethylidene)furan-2,4(3*H*,5*H*)-dione (**4p**). Pale yellow powder; yield 67% (0.28 g); mp 110-112°C. ^{1}H NMR (300 MHz, DMSO-d_6) δ 9.77 (br. s, 1H), 7.36 – 7.18 (m, 3H), 7.18 – 7.11 (m, 1H), 4.39 (t, J = 7.1 Hz, 2H), 2.79 (t, J = 7.1 Hz, 2H), 2.47 (s, 2H), 2.46 (s, 3H), 2.28 – 2.13 (m, 2H), 1.90 – 1.74 (m, 2H), 1.68 – 1.58 (m, 2H), 1.57 – 1.44 (m, 2H). ^{13}C NMR (75 MHz, DMSO-d_6) δ 192.2, 177.8, 175.3, 164.6, 140.7, 132.8, 130.0, 128.9, 127.8, 126.3, 125.2, 99.0, 49.0, 44.8, 43.7, 32.4, 25.0, 23.6. HRMS (ESI-TOF) *m/z*: Calcld for $C_{22}H_{23}ClNO_5$ $[M+H]^+$ 416.1259; Found: 416.1271.

(3*E*,5*E*)-5-(3,3-dimethyl-5-oxo-1-phenethylpyrrolidin-2-ylidene)-3-(1-hydroxyethylidene)furan-2,4(3*H*,5*H*)-dione (**4q**). White powder; yield 59% (0.21 g); mp 171-173°C. ^{1}H NMR (300 MHz, DMSO-d_6) δ 7.32 – 7.13 (m, 5H), 4.34 (t, J = 7.6 Hz, 2H), 2.76 (t, J = 7.7 Hz, 2H), 2.46 (s, 3H), 2.44 (s, 2H), 1.34 – 1.28 (m, 6H). ^{13}C NMR (75 MHz, DMSO-d_6) δ 192.5, 177.9, 175.1, 164.6, 146.3, 138.0, 128.9, 128.3, 128.2, 126.3, 125.5, 99.0, 44.1, 43.7, 39.4, 32.6, 26.1, 23.7. HRMS (ESI-TOF) *m/z*: Calcld for $C_{20}H_{22}NO_5$ $[M+H]^+$ 356.1492; Found: 356.1487.

(3*E*,5*E*)-3-(1-hydroxyethylidene)-5-(2-(4-methoxyphenyl)-3-oxo-2-azaspiro[4.5]decan-1-ylidene)furan-2,4(3*H*,5*H*)-dione (**4r**). Yellow powder; yield 37% (0.15 g); mp 190-192°C. ^{1}H NMR (300 MHz, DMSO-d_6) δ 8.11 (br. s, 1H), 7.17 – 7.05 (m, 2H), 6.99 – 6.87 (m, 2H), 3.85-3.67 (m, 3H in H_2O), 2.80 – 2.71 (m, 2H), 2.31 (s, 3H), 2.27 – 2.15 (m, 2H), 1.70 (d, J = 12.0 Hz, 4H), 1.46 – 1.29 (m, 2H). ^{13}C NMR (75 MHz, DMSO-d_6) δ 194.8, 175.1, 164.4, 158.2, 130.7, 128.4, 127.5, 113.7, 113.5, 55.2, 44.9, 32.1, 25.19, 24.8, 22.2, 21.8. HRMS (ESI-TOF) *m/z*: Calcld for $C_{22}H_{24}NO_6$ $[M+H]^+$ 398.1598; Found: 398.1611.

(3*E*,5*E*)-3-(1-hydroxyethylidene)-5-(2-methyl-3-oxo-8-thia-2-azaspiro[4.5]decan-1-ylidene)furan-2,4(3*H*,5*H*)-dione (**4s**). Pale brown powder; yield 62% (0.20 g); mp 207-

209°C. ^{1}H NMR (300 MHz, DMSO-d_6) δ 3.26 (s, 3H), 2.75 (t, *J* = 12.9 Hz, 2H), 2.64 (s, 2H), 2.58-2.35 (m, 4H), 2.43 (s, 3H), 1.79 (d, *J* = 12.6 Hz, 2H). ^{13}C NMR (75 MHz, DMSO-d_6) δ 193.2, 177.1, 175.2, 164.8, 147.0, 125.3, 98.5, 43.8, 37.9, 32.8, 32.6, 24.7, 23.6. HRMS (ESI-TOF) *m/z*: Calcld for $C_{15}H_{18}NO_5S$ $[M+H]^+$ 324.0900; Found: 324.0901.

(3E,5E)-5-(2-cyclohexyl-3-oxo-2-azaspiro[4.5]decan-1-ylidene)-3-(1-hydroxyethylidene)furan-2,4(3H,5H)-dione and *(3E,5Z)-5-(2-cyclohexyl-3-oxo-2-azaspiro[4.5]decan-1-ylidene)-3-(1-hydroxyethylidene)furan-2,4(3H,5H)-dione* (mixture of E/Z isomers 5:1) (**4t**). White powder; yield 51% (0.19 g); mp 191-193°C. ^{1}H NMR (300 MHz, DMSO-d_6) δ 4.35 – 4.23 (m, 0.2H), 3.92 (t, *J* = 12.0 Hz, 0.8H), 2.61 (s, 2H), 2.57 (s, 3H), 2.33 – 2.12 (m, 4H), 1.86 – 1.76 (m, 4H), 1.75 – 1.59 (m, 5H), 1.58 – 1.49 (m, 1H), 1.45 – 1.09 (m, 6H). ^{13}C NMR (75 MHz, DMSO-d_6) δ 193.5, 193.2, 176.6, 176.4, 175.8, 150.7, 150.6, 125.4, 123.5, 99.2, 98.6, 60.6, 57.4, 45.4, 42.6, 41.3, 32.0, 31.6, 29.1, 25.7, 24.9, 24.8, 24.2, 23.9, 23.9, 23.9, 22.2, 21.8. HRMS (ESI-TOF) *m/z*: Calcld for $C_{21}H_{29}NO_5$ $[M+H]^+$ 374.1962; Found: 374.1969.

Analytical data for compound 7

(3*E*,5*E*)-3-(1-hydrazineylethylidene)-5-(3-oxo-2-phenethyl-2-azaspiro[4.5]decan-1-ylidene)furan-2,4(3*H*,5*H*)-dione (**7**). White powder; yield 71% (0.29 g); mp 199-201°C. ^{1}H NMR (300 MHz, DMSO-d_6) δ 12.11 (br. s, 1H), 7.30 – 7.12 (m, 5H), 5.72 (s, 2H), 4.47 (t, *J* = 7.5 Hz, 2H), 2.68 (t, *J* = 7.7 Hz, 2H), 2.56-2.48 (m, 2H in DMSO), 2.46 (s, 3H), 2.15 (t, *J* = 12.9 Hz, 2H), 1.63 – 1.53 (m, 2H), 1.40 – 1.11 (m, 5H). ^{13}C NMR (75 MHz, DMSO-d_6) δ 179.8, 175.3, 164.8, 140.4, 138.4, 129.0, 128.4, 128.3, 126.3, 88.98, 43.7, 43.5, 39.7, 32.8, 32.3, 24.9, 22.0, 12.8. HRMS (ESI-TOF) *m/z*: Calcld for $C_{23}H_{28}N_3O_4$ $[M+H]^+$ 410.2074; Found: 410.2092.

Analytical data for compound 9

(3*E*,5*E*)-3-(1-((3-chlorobenzyl)amino)ethylidene)-5-(3-oxo-2-phenethyl-2-azaspiro[4.5]decan-1-ylidene)furan-2,4(3*H*,5*H*)-dione (**9**). White powder; yield 65% (0.34 g); mp 158-160°C. ^{1}H NMR (300 MHz, DMSO-d_6) δ 11.59 (s, 1H), 7.53 – 7.41 (m, 3H), 7.41 – 7.35 (m, 2H), 7.23 – 7.10 (m, 4H), 4.82 (d, *J* = 6.1 Hz, 2H), 4.42 (t, *J* = 7.6 Hz, 2H), 2.67 (t, *J* = 7.8 Hz, 2H), 2.57 (s, 3H), 2.54 (s, 2H), 2.16 (t, *J* = 12.9 Hz, 2H), 1.69 – 1.53 (m, 3H), 1.42 – 1.08 (m, 5H). ^{13}C NMR (75 MHz, DMSO-d_6) δ 175.7, 171.6, 139.4, 138.7, 138.7, 136.8, 136.6, 136.0, 134.0, 131.3, 129.4, 128.6, 128.4, 128.1, 127.9, 127.8, 126.7, 46.4, 44.3, 44.0, 33.3, 32.6, 25.2, 22.4, 14.8. HRMS (ESI-TOF) *m/z*: Calcld for $C_{30}H_{32}ClN_2O_4$ [M+H]$^+$ 519.2045; Found: 519.2051.
